# Enteric Microbiota–Gut–Brain Axis from the Perspective of Nuclear Receptors

**DOI:** 10.3390/ijms19082210

**Published:** 2018-07-28

**Authors:** Kalina Duszka, Walter Wahli

**Affiliations:** 1 Department of Nutritional Sciences, University of Vienna, Althanstrasse 14, 1090 Vienna, Austria; kalina.duszka@univie.ac.at; 2 Lee Kong Chian School of Medicine, Nanyang Technological University, 11 Mandalay Road, Singapore 308232, Singapore; 3 Center for Integrative Genomics, University of Lausanne, Génopode, CH-1015 Lausanne, Switzerland

**Keywords:** nuclear receptors, microbiota, metabolism, xenobiotics

## Abstract

Nuclear receptors (NRs) play a key role in regulating virtually all body functions, thus maintaining a healthy operating body with all its complex systems. Recently, gut microbiota emerged as major factor contributing to the health of the whole organism. Enteric bacteria have multiple ways to influence their host and several of them involve communication with the brain. Mounting evidence of cooperation between gut flora and NRs is already available. However, the full potential of the microbiota interconnection with NRs remains to be uncovered. Herewith, we present the current state of knowledge on the multifaceted roles of NRs in the enteric microbiota–gut–brain axis.

## 1. Introduction

Nuclear receptors (NRs) and microbiota are two factors that together have a great impact on all body functions. With the cloning of the first NRs in the 1980s, a great era of NR research begun. The progressive discovery of the 48, 49 and 47 NRs in humans, mice and rats, respectively, and their essential role in development, metabolism, homeostasis by directing virtually each single process in these organisms, powered abundance of prospective research in this new field [1]. The prosperity of NR research partially overlapped with increasing interest in the gut microbiota and its recognized major regulatory function. An extraordinary profusion of microbiota research at the beginning of 21st century changed our perception of gut bacteria from symbiotic organisms to forming a functional “organ” modulating the host’s physiological and psychological health. Within the last years, research in the field of microbiota gradually progressed from studies based on correlations between microbiota composition and diseases (e.g., autism, obesity, metabolic and autoimmune diseases) to tracking molecular pathways of interactions between specific bacterial strains, their metabolites and the host. What we have learned so far is that gut microbiota is characterized by its high variability in composition between different hosts, which is induced by multiple factors including childbirth delivery, gender, life style, diet, age, the individual’s immune system, immunization, pharmacological interventions, antibiotic treatments, contacts with other carriers of bacteria, length of exposure to factors affecting bacterial composition, and geographical location [2,3,4,5]. Moreover, metabolites produced by the gut flora itself also influence microbial communities in the gut, modulating their composition, maintaining their optimal population size and protecting them from colonization by pathogens through the production of bacteriocins [6]. Recently, it has been shown that the human gut microbiota composition is influenced more by environmental factors than by the host’s genome. In fact, over 20% of inter-person microbiota variability is associated with factors related to diet, drugs and anthropometric characteristics [7]. Based on the current state of research, we can conclude that: (1) the gut microbiota is a complex and dynamic part of the body; (2) it is impossible to define a single and proper healthy gut bacteria composition that would fit all due to highly individual external and innate factors shaping distinct gut microbiota. Therefore, trying to compare and evaluate the determinants of microbiota composition is a complex task. Moreover, studies in germ-free (GF) mice have shown that the gut bacteria as an “organ” is not indispensable to live and thrive, at least in a laboratory environment. However, the regulatory function of the microbiota with specific roles in gut, brain and whole organism development and maintenance definitely represents an evolutionary benefit. During the long natural history of the symbiosis between host and microbiota, resident gut bacteria developed multiple ways of communicating with their host including with its central nervous system (CNS), which resulted in the development of a key microbiota–gut–brain axis. The aim of this article is to review existing evidence of the involvement of NRs in the interaction between host and gut microbiota, particularly in the context of the gut–brain axis.

## 2. Gut–Brain Axis

By now, the interaction between the gut microbiota and the brain is an evidence-based fact. Data suggest that the gut microbiota can affect physiological, behavioral and cognitive functions of the brain [8,9,10,11,12]. Bidirectional signaling in the enteric microbiota–gut–brain axis is regulated at neural, hormonal, and immunological levels and includes the CNS, neuroendocrine and neuroimmune systems, the enteric and autonomic (sympathetic and parasympathetic branches) nervous system, and intestinal microbiota factors. Visceral signals are transmitted directly to the CNS via afferent fibers or indirectly by the blood circulation. Response signals from the CNS are mediated via efferent fibers to the smooth muscles of the gastrointestinal (GI) tract, thereby influencing its motility and secretory functions, which modify the environment in which the bacteria reside [12,13,14,15]. Host mental state, especially under stress, has long-term effects on gut flora [16,17,18]. Stress increases gut permeability and modulates growth of both non-pathogenic and pathogenic bacteria via the effects of dopamine, adrenaline and noradrenaline produced by the host [19,20,21,22,23,24,25,26]. Importantly, adrenaline and noradrenaline also modulate the expression of bacterial virulence genes [26,27].

As mentioned above, gut–brain signaling works both ways; thus, signals sent by resident bacteria affect the whole body. The GI flora secrete an abundance of microbe-associated molecular patterns (MAMPs) and bioactive metabolites such as bacteriocins, secondary bile acids (BA), choline and short chain fatty acids (SCFAs). SCFAs are derived from the fermentation of polysaccharides and influence the colon epithelium locally, but also enter the blood circulation and modulate the host’s incretin production and energy balance [28]. By regulating the levels of satiety and hunger hormones, including ghrelin [29,30], leptin [29,31,32,33], insulin [34], somatostatin [35], peptide YY (PYY) [30] and glucagon-like protein-1 (GLP-1) [36,37], bacteria affect the host’s gut motility, nutrients absorption, glucose tolerance, food cravings and hunger. Stunningly, gut flora may impact the levels of hunger hormones by influencing the production of autoantibodies against peptide hormones involved in appetite control [38]. Bacterial colonization of the intestine has a major role in post-natal development and maturation of the endocrine, immune and nervous systems and affects the host’s physiological and psychological health [8,39,40,41,42,43,44,45]. Enteric microbiota also regulates levels of sex hormones; transferring the gut microbiota of male mice to females causes an elevation in testosterone levels and induces metabolomic changes in recipient females [46]. Commensal flora itself is capable of synthesizing and releasing many neurotransmitters and neuromodulators, or inducing enteroendocrine cells to synthetize and release neuropeptides, thereby affecting the host’s behavior and stress levels. Bacteria play a critical role in the production of free adrenaline in the gut lumen [47] and *Escherichia*, *Bacillus* and *Saccharomyces* spp. can generate noradrenaline [48,49]. Furthermore, the microbiota affects the hippocampus and amygdala levels of brain-derived neurotrophic factor (BDNF), a key neurotrophin involved in neuronal growth and survival [42]. *Lactobacillus* and *Bifidobacterium* species can synthetize γ-aminobutyric acid (GABA), while *Lactobacillus rhamnosus* (*JB-1*) induces region-dependent alterations in GABA(B1b) and GABA(Aα2) receptors in various parts of the brain [45,48,49]. Importantly, vagotomized mice do not display the neurochemical and behavioral effects of *L. rhamnosus* (*JB-1*), thus implicating the vagus nerve in the direct communication between bacteria and the brain [45]. Alterations in central GABA receptor expression are implicated in the pathogenesis of anxiety and depression, which are highly comorbid with functional bowel disorders. Moreover, *Bacillus* can produce dopamine, *Lactobacillus* can generate acetylcholine, and *Candida*, *Streptococcus*, *Escherichia* and *Enterococcus* spp. synthetize serotonin (5-HT) [48,49]. 5-HT is a key regulator of GI motility and secretion but also a modulator of depression and anxiety-like behavior. Approximately 95% of 5-HT in the body is compartmentalized in the gut, predominantly in enterochromaffin cells of the mucosa and in nerve terminals of the enteric nervous system. The presence of specific strains of bacteria or the GF status in mice modulate 5-HT production by the enterochromaffin cells, which affects circulating levels of 5-HT and tryptophan, a 5-HT precursor [50,51,52,53]. Interestingly, the delivery of the probiotics *Bifidobacterium infantis* 35624, which results in an elevation in plasma tryptophan, has been suggested as a promising anti-depressive therapy [53]. Thus, the continuous dialog between the GI flora and brain—regulating parts of postnatal development, metabolism and daily body functioning—is essential for a healthy brain and gut.

## 3. Nuclear Receptors

NRs together form a superfamily of proteins that function as transcription factors. In principle, their activation requires the binding of ligands, which results in increased affinity towards NR-response elements in the regulatory region of target genes leading to changes in their expression activity. Multiple NRs are expressed in the GI tract, and several microbe-produced metabolites act as ligands of NRs. Thereby, the activity of NRs may be affected by nutrient- and microbiota-derived ligands. Gut bacteria secrete metabolites including indole derivatives, hormones and secondary BAs, which act as natural ligands for the host’s NRs [54]. Thus, microbial metabolites influence the host by regulating gene expression that modulates biological effects. This interactive system allows a direct influence of the microbiota on the host’s physiology and, therefore, it can have control over the health or disease status of the host.

### 3.1. PPARs

**Figure 1:**
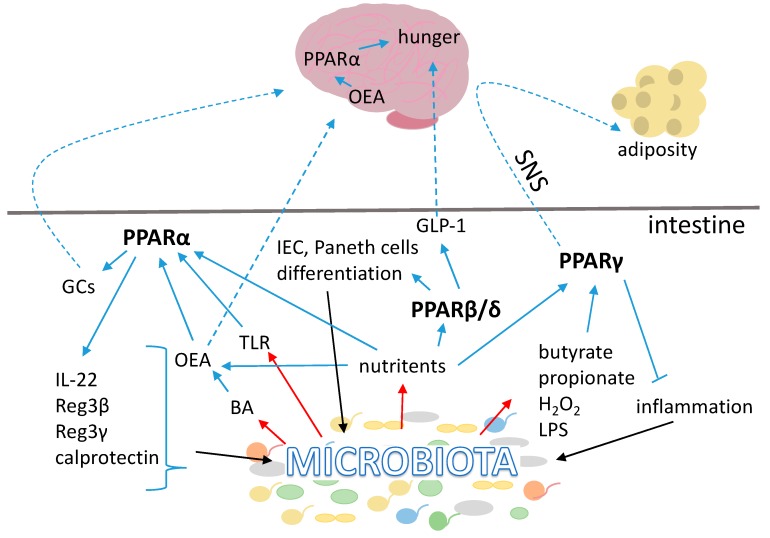
PPARs in gut–brain signaling. PPARs are activated by nutrient derivatives as well as bacteria-derived molecules and their signaling. PPARα serves as a receptor for oleoylethanolamide (OEA) and mediates the impact of OEA on hunger and satiety. PPARα also impacts the central nervous system (CNS) by taking part in microbiota-mediated synthesis of glucocorticoids (GCs). PPARβ/δ mediates satiety signals from gut to the CNS by stimulating the synthesis of glucagon-like protein-1 (GLP-1). Intestinal PPARγ affects adiposity via the sympathetic nervous system (SNS). Red arrows symbolize direct signals from enteric bacteria, black arrows indicate consequences of nuclear receptor (NR) signaling on the bacteria and blue arrows illustrate interactions between the pictured factors. Blue solid lines correspond to interactions within intestine or the brain while blue dotted lines mark signaling between the organs.

Peroxisome proliferator activated receptors (PPARs) are a sub-family of ligand-activated nuclear receptors, which consists of three isotypes; PPARα (NR1C1), PPARβ/δ (NR1C2) and PPARγ (NR1C3), each produced by a separate gene [55]. PPARs are collectively involved in the control of energy metabolism, inflammatory and immune responses. Natural ligands of PPARs include fatty acids (FA), eicosanoids and phospholipids [56,57]. Each PPAR isotype shows an individual pattern of expression in the GI tract and exerts different functions [58]. However, all three of them are known for their anti-inflammatory properties in the small intestine and colon. All PPARs mediate microbiota effects and take part in the gut–brain axis signaling; however, the way they accomplish this function is distinct (Figure 1). If their relation with the microbiota is mainly in the context of their anti-inflammatory properties, signaling to the brain is mostly related to metabolism. These two functions are highly interconnected in the GI tract as shown by our recent caloric restriction study in mice [59].

#### 3.1.1. Peroxisome Proliferator Activated Receptor α

PPARα was initially identified as the molecular target of xenobiotics inducing peroxisome proliferation in rodents [60]. PPARα is ubiquitously expressed and is particularly abundant in organs with a high demand for catabolism of fatty acids [58]. The greatest expression of this isotype is in liver and brown adipose tissue (BAT); the stomach and duodenum also show substantial levels of PPARα. The expression of PPARα decreases along the GI tract with lowest levels in the colon [58].

PPARα coordinates several aspects of metabolism by modulating the expression of genes involved in peroxisomal and mitochondrial β-oxidation, FA transport, FA catabolism, ketogenesis and gluconeogenesis [56]. PPARα is a nutritional status sensor and allows adaptation of the rates of FA catabolism, lipogenesis and ketone body synthesis in response to nutritional status, particularly during fasting [61,62,63]. During the fed state, PPARα synchronizes pathways of de novo lipid synthesis to supply FAs for storage. In starvation, when the organism shifts to the mobilization of stored FAs, PPARα switches its activity to promote cellular FA uptake and β-oxidation. Moreover, PPARα stimulates the expression of rate-limiting enzymes of ketogenesis in the liver [64,65]. Importantly, the role in ketone body synthesis is dependent on the stimulation of PPARα expression in the liver by commensal gut microbiota [66]. Thus, microbiota-dependent PPARα activity modulates proper metabolic adjustment to nutrient availability. Given these facts, it is not surprising that PPARα has been identified as a major factor in the adjustment of metabolism during caloric restriction and daily rhythms (rest/activity and fasting/feeding) [59,67,68,69,70] 

PPARα is a major contributor to functional circadian rhythm by directly regulating transcription of the important circadian genes *Bmal1* and *Rev-erbα* [68,71], as well as modulating PER2 activity by direct protein–protein interactions [67]. Additionally, *PPARα* itself is a target gene of BMAL1 and CLOCK [68,72]. As a result, numerous genes involved in lipid and cholesterol metabolism, as well as energy homeostasis, which are regulated by PPARα, display daily fluctuations in mouse liver [73,74]. It is noteworthy that PPARα, by mediating signals received from the microbiota via toll-like receptors (TLR), contributes to both the circadian expression of genes in the intestine and intestinal corticosterone production [75]. Thus, PPARα mediates signals from the GI flora, which impact the host’s physiology.

An additional link between PPARα and microbiota has been proposed recently [76]. PPARα was identified as an important factor in the inflammatory response of the intestine to commensal microbiota. According to the model proposed, PPARα regulates the expression of interleukin 22 (IL-22) and the antimicrobial peptides Reg3β, Reg3γ, and calprotectin. In mice deficient in PPARα, commensal dysbiosis in the gut occurs and results in an increased expression of inflammatory cytokines and enhanced susceptibility to intestinal inflammation [76]. These findings comply with previous reports associating intestinal PPARα with anti-inflammatory activity in the GI tract by preventing neutrophil infiltration and protecting the intestine from colitis-induced permeability [77,78,79,80].

Even more interestingly, intestinal PPARα is involved in inducing satiety signals in the brain. Oleoylethanolamide (OEA), an endogenous ligand of PPARα, is an endocannabinoid produced by enterocytes in response to fat consumption [81]. Biosynthesis of OEA in the intestine requires sympathetic innervation [82] and is modulated by BAs [83]. Accordingly, hepatic expression of PPARα is stimulated by the farnesoid X receptor (FXR), which with the support of microbiota, controls BA metabolism (for details see the further parts of this review below) [84]. Administration of OEA has an anorectic effect by acting peripherally, prolonging eating latency or reducing meal size, depending on the nutritional state, and leads to body weight reduction [81,85,86,87]. This effect is mediated by PPARα activation in the proximal small intestine [81,88,89]. Surprisingly, intraperitoneal administration of OEA acutely decreases energy expenditure, as well as ambulatory and spontaneous locomotor activity [90]. It regulates lipid metabolism by activating PPARα to stimulate lipolysis, and decreases neutral lipid content in hepatocytes as well as serum cholesterol and triglyceride levels [91]. OEA in intestinal enterocytes engages afferent sensory fibers of the vagal nerve leading to increased expression of c-fos in the nucleus solitary tract (NST) and the paraventricular nucleus (PVN) of the brainstem and hypothalamus, respectively [91], which stimulates oxytocin secretion and promotes satiety [92]. Since enterocytes in the small intestine are the first cells responding to dietary fat intake by increasing OEA production, OEA was suggested to serve as a gut-derived satiety factor [81].

#### 3.1.2. Peroxisome Proliferator Activated Receptor β/δ

PPARβ/δ is ubiquitously expressed; nevertheless, its expression in the GI tract is very high compared with other tissues. PPARβ/δ is abundant in the gut from the duodenum to the ileum, with lesser but still detectable expression in the colon [58]. Pparβ/δ is constitutively expressed in the intestine, but inflammatory signals further stimulate its expression [93]. Intestinal PPARβ/δ induces terminal differentiation of epithelial cells in the intestine and colon and is required for the differentiation of Paneth cells [94,95,96]. Thus, PPARβ/δ is indirectly involved in secretion of antimicrobial peptides. PPARβ/δ protects against dextran sulphate sodium (DSS)-induced colitis [97]; however, its role in colon cancer has been controversial and conflicting results suggest that PPARβ/δ can either promote or attenuate this disease [98].

When activated, intestinal PPARβ/δ promotes fatty acid oxidation in adipose tissue and skeletal muscle, it improves dyslipidemia and stimulates overall energy expenditure thus protecting against diet-induced obesity and insulin resistance [99]. It regulates plasma HDLc levels, influences expression of genes involved in lipoprotein metabolism and stimulates postprandial GLP-1 production in enteroendocrine L-cells, resulting in preservation of pancreas β-cell morphology and function thereby increasing systemic insulin sensitivity [100]. However, the detailed mechanisms behind these beneficial properties remain to be explored further. Thus, besides enhancing the satiety signal GLP-1, no other link between intestinal PPARβ/δ and the CNS has been identified to date.

#### 3.1.3. Peroxisome Proliferator Activated Receptor γ 

PPARγ is mostly known for its insulin sensitizing properties and its role as a master regulator of adipogenesis [101]. It modulates multiple processes, including cell proliferation, differentiation, glucose and lipid metabolism, and inflammation [101,102,103,104,105]. PPARγ is highly expressed in adipocytes and in the gut, and at lower levels in the pancreas, liver, kidney, and immune cells. In the GI tract, it is present at a relatively high level in the proximal parts of the small intestine and gradually decreases towards its distal parts. However, it is highly expressed in the proximal colon [58,106,107,108]. PPARγ expression and activity are induced in the gut by multiple nutrients, most importantly by fatty acids and their metabolites but also by glutamine, curcumin, capsaicin, ginsenosides and vitamin E, all of which have been reported to exhibit anti-inflammatory properties [109]. Importantly, bacterial metabolites and bacterial by-products, such as butyrate [110,111], propionate [112] and H_2_O_2_ [113] also stimulate expression or activity of PPARγ. Presence of specific bacterial strains, such as *Enterococcus faecalis*, *Roseburia hominis*, *Roseburia intestinalis*, *Fusobacterium naviforme* and *Streptococcus salivarius* influence the phosphorylation of PPARγ and thereby its transcriptional activity [112,114,115]. Moreover, it has been shown that the microbiota affects liver circadian rhythm by modulating the activity of PPARγ expressed in liver [116]. Thus, there is a dynamic balance involving reciprocal interactions between PPARγ and the gut microbiota, whereby PPARγ can both be activated by bacteria and regulate the intestinal microbiota composition.

PPARγ has been identified as a promising therapeutic target in fighting colon cancer as it reduces colorectal tumor development by decreasing cell proliferation [117,118,119], increasing cell differentiation [117,120], inducing apoptosis [117,118,121,122,123], and inhibiting angiogenesis [124]. PPARγ agonists mitigate inflammatory bowel disease (IBD) symptoms, reduce inflammation, and are effective in multiple models of ulcerative colitis as well as in Crohn’s disease [125,126,127,128,129,130,131,132,133,134,135,136,137,138]. PPARγ acts as anti-inflammatory mediator by regulating multiple signaling pathways, including those related to p53 [139], Bcl2 [121,123], c-Myc, [140], Cox-2 [123,141,142,143], Apc/B-catenin [144,145] and NF-κB [121,143]. Notably, PPARγ is responsible for the selective killing of bacteria associated with IBD and maintenance of innate antimicrobial immunity in the colon [146]. Hence, bacteria-mediated stimulation of gene activity by PPARγ appears to have a protective role in the prevention of gut inflammation and regulation of immune tolerance. Recently, we described a novel role of intestinal PPARγ in long chain FA processing in the intestinal epithelium [147]. This is an original finding given the fact that, so far, intestinal PPARγ has primarily been characterized in the context of inflammation and cancerogenesis. Importantly, using an intestinal epithelium-specific PPARγ knockout mouse model, we found a signaling mechanism between PPARγ expressed in intestinal epithelium and the brain [148]. Intestinal PPARγ in mice submitted to caloric restriction or a diet low in sucrose regulates body adiposity by signaling via the sympathetic nerve system. Thus, activation of intestinal PPARγ by bacterial metabolites or nutrients can impact adipose tissue via the nervous system.

### 3.2. Farnesoid X Receptor (FXR)

BAs produced by the liver are stored in the gall bladder and secreted into the small intestine where they contribute to the emulsification and solubilization of fats. Thus, they aid in the digestion and absorption of lipids. The expression profile of genes involved in BA synthesis, conjugation, and reabsorption is altered by the resident gut microbiota [149]. Certain secreted BAs undergo biotransformation by the gut microbiota from primary to secondary BAs [150], and this process is accompanied by a feedback mechanism of BAs on the microbiota composition [151,152]. Moreover, secondary BAs, but also some of the primary BAs, can act as ligands for the nuclear receptor FXR (NR1H4), which is expressed in enterocytes throughout the small intestine and colon [153]. BA activation of FXR leads to subsequent alteration of the BA pool size via two FXR-dependent feedback mechanisms of hepatic BA synthesis [154]. FXR regulates the transcription levels of critical genes in BA synthesis, homeostasis, transport and metabolism [155] but also affects general lipid and glucose metabolism [156], as well as hepatic autophagy [157,158]. Mice lacking FXR expression in the intestine withstand diet-induced obesity (DIO), insulin resistance and non-alcoholic fatty liver disease, and several studies associated FXR with sensitivity to metabolic diseases [149,159,160,161,162]. FXR knockout may be mimicked by a treatment with tempol (4-hydroxy-2,2,6,6-tetramethyl-piperidine-*N*-oxyl) a stable nitroxide which is a membrane-permeable, and metal-independent superoxide dismutase (SOD) mimetic. Tempol decreased the *Lactobacillus* population in gut, accompanied by reduced activity of bile salt hydrolase (BSH) normally exhibited by the bacteria. As a result, a potent antagonist of FXR, tauro-β-muricholic acid (T-β-MCA) accumulated in the intestine, demonstrating that the enteric flora can modulate the activity of FXR [159]. Moreover, the gut microbiota has been shown to promote DIO and stimulate adiposity via activation of the intestinal BA/FXR axis, which contributes to alteration of the ceramide/SREBP1C/CIDEA pathway in the liver and disrupts lipid homeostasis [149,160]. Conversely, FXR may contribute to increased adiposity by modulating the composition of the gut microbiota [149]. Thus, the tie between FXR and microbiota is tight and complex and, therefore, difficult to dissect. However, it is sufficiently documented that targeting bacteria or BA composition can result in changes of FXR activity and body weight.

A link was identified between the BAs in the gut and cystic breast tissue in humans [163,164]. The bile salt lithocholate originating from the intestine was found in aspirates of cyst fluid from the breasts of women with fibrocystic disease at much higher concentrations than in the serum [163]. Additionally, BAs such as deoxycholic acid (DCA) have been shown to stimulate both the growth and metastasis of breast cancer cells through FXR expressed in the breast cancer tissue [165], suggesting that BAs may play a role in breast tumor carcinogenesis.

Among other genes, FXR binds a response element in the regulatory region of *Fgf15* (a rodent ortholog to human *Fgf19*) and directly regulates its transcription. FGF15 is produced in the distal small intestine, and is secreted into the portal circulation to function as a postprandial hormone [166,167]. Its levels rise following a meal with a lag comparable to that of insulin. In contrast to other enterokines (e.g., GLP-1, GIP), FGF15 does not affect insulin secretion but rather mimics insulin impact [167,168]. The peak of FGF15 occurs 90–120 min after the postprandial release of BAs, precedes the repression of BA synthesis [167], and causes the gallbladder to refill with bile [169]. Secreted FGF15 circulates to the liver, where it acts on two receptors: FGF-receptor 4 (FGFR4) and β-Klotho (KLB). This results in the activation of the Ras–ERK–p90RSK pathway, which affects the expression of genes, such as *c-Fos*, *JunB*, and *c-Jun* [168,170]. FGF15 inhibits BA synthesis in the liver by repressing the transcription of the cholesterol 7α-hydroxylase gene (*Cyp7a1*), which encodes the first and rate-limiting enzyme in the classic BA synthesis pathway [171]. FGF15 also stimulates hepatic protein and glycogen synthesis [172], contributes to the regulation of systemic lipid and glucose metabolism [173] and represses gluconeogenesis through a mechanism involving the dephosphorylation and inactivation of the transcription factor CREB [174]. Importantly, according to the current state of knowledge FGF15 represents a key factor in the FXR–gut–brain axis connection. FGF15 secreted from the intestine leads to activation of its receptors in hypothalamic AGRP/NPY neurons leading to reduction of signaling by these neurons. This regulation promotes glucose tolerance presumably mediated by the autonomic nervous system [175]. Thus, FGF15 can modulate metabolism by acting directly on the brain, which is supported by the observation according to which intra-cerebro-ventricular injection of FGF15 reduces 24 h food intake and body weight, and acutely improves glucose tolerance [176]. In *ob/ob* mice, FGF15 administered centrally increases glucose disposal via an insulin-independent mechanism [177]. Remarkably, FGF15-triggered nerve signaling is required for longer-term weight loss and glycemic effects while its activity in the liver and adipose tissue is not [178]. Notably, expression of human FGF19-receptors 1 and 4 in the hypothalamus of obese rats is reduced by 60% relative to lean animals [176]. It has been shown that intestinal PPARα affects FXR-FGF15 activity and BA synthesis [179] and BA feedback inhibits PPARα activity [180]. Thus, we can speculate that gut–brain communication involves the microbiota and at least two NR networks, in other words a microbiota–PPARα–FXR–FGF15–gut–brain axis.

### 3.3. Vitamin D Receptor

Synthesis of vitamin D is initialized when UVB light triggers photochemical, non-enzymatic conversion of 7-dehydrocholesterol to previtamin D_3_ in keratinocytes of the skin. Consecutively previtamin D_3_ undergoes thermal isomerization to vitamin D_3_ (cholecalciferol). Following circulation in the bloodstream, vitamin D_3_ is taken up by liver and modified to the prohormone calcifediol (25-hydroxyvitamin D_3_, 25(OH)D_3_). Additionally, vitamin D_2_ or ergocalciferol, which can be provided by nutrition, including sources like fish, egg and mushrooms, serves as another precursor that may be modified to calcifediol. Further conversion of calcifediol into calcitriol (1,25-dihydroxyvitamin D, 1,25(OH)_2_D) is executed by 1α-hydroxylase (CYP27B1) and takes place mostly in the kidneys. Nonetheless, 1α-hydroxylase is also expressed in other tissues including colon, breast tissue and immune cells [181,182,183,184,185]. Levels of vitamin D in the blood show a strong relationship with infection rates, bone health, cancer prophylaxis and mortality [186,187,188,189,190]. Calcifediol and calcitriol serve as ligands for the vitamin D receptor (VDR, NR1I1), the second being a more potent activator [191]. Additionally, the secondary bile acid lithocholic acid, curcumin, γ-tocotrienol, and derivatives of essential fatty acids also act as ligands of VDR [192,193]. Upon activation by ligands, VDR activates or represses expression of its target genes. VDR is particularly important for the homeostasis of minerals by regulating the intestinal uptake of calcium, iron, magnesium, zinc, copper and selenium. VDR also regulates calcium reabsorption in kidneys and promotes bone calcification. Notably, VDR also facilitates intestinal absorption of toxic elements like lead, aluminum, cadmium, cobalt and strontium [194]. VDR is expressed throughout the body with high levels in the intestine, colon, thyroid gland and kidney. Its expression in the brain remains to be clarified as there are contradictory reports on this subject [195,196,197]. Importantly, vitamin D can barely diffuse through the blood brain barrier (BBB) [198] however, 1α-hydroxylase is expressed in the CNS [199,200] suggesting a role in the brain.

VDR plays an important role in the immune response, mediates signals from the microbiota and translating them into appropriate inflammatory responses [201,202,203]. VDR influences the expression of defensins, the antimicrobial function of Paneth cells and colonization of the GI tract by bacteria [204,205,206]. Consequently, the microbiota of VDR knockout mice differs when compared to wild type mice [207]. Intestine epithelium-specific VDR knockout mice are also more susceptible to DSS-induced colitis [206]. The TLR pathway when activated by pathogen-associated membrane patterns (PAMPs) of *Mycobacterium tuberculosis* stimulates VDR expression. In turn, VDR is necessary for the generation of the antimicrobial peptides cathelicidins by monocytes-macrophages and the killing of intracellular *Mycobacterium tuberculosis* [208,209]. Moreover, the probiotic *Lactobacillus rhamnosus* strain GG and *Lactobacillus plantarum* trigger the expression and transcriptional activity of VDR and thereby protect from *Salmonella*-induced colitis in a VDR-dependent way [210]. Reciprocally, microbiota regulates local production of vitamin D_3_ in the colon by signaling through the TLR pathway [211].

VDR is a vital factor in brain development [212], neurotransmission [213], neuroprotection [214,215] and immunomodulation [216,217]. Vitamin D deficiency has been linked with occurrence of autism [218,219], schizophrenia [220,221,222], multiple sclerosis [223,224], Alzheimer’s and Parkinson’s disease [225]. However, the putative mechanisms linking VDR to neurological diseases remain to be unveiled. Moreover, a direct role of VDR in the gut–brain axis has not been established so far.

### 3.4. Xenobiotic Receptors

#### 3.4.1. Aryl Hydrocarbon Receptor

Aryl hydrocarbon receptor (AHR) is not per se a member of the NR superfamily, although it shares many characteristics with family members. AHR is a ligand-activated receptor expressed in many cell types, including intestinal epithelial cells (IEC). AHR is mostly recognized as a molecule activated by the xenobiotic 2,3,7,8-tetrachlorodibenzo-*p*-dioxin (TCDD, also known as dioxin) [226,227]. However, the list of its agonists is much longer and contains various groups of molecules including diet-derived compounds (e.g., flavonoids, carotenoids, stilbenes) [228,229,230], metabolites of tryptophan produced by the host, such as kynurenine (KYN) [231] or kynurenic acid (KYNA) [232], and by the microbiota (indoles) [233,234]. Also, phenazine produced by *Pseudomonas aeruginosa*, and naphthoquinone phthiocol from *Mycobacterium tuberculosis* bind to AHR, which triggers cytokine and chemokine production [235]. Moreover, the bacterial metabolites SCFAs modulate AHR activity, however not directly as ligands, but they stabilize and facilitate AHR actions [236]. Intestinal AHR affects the differentiation and function of T cells, macrophages, and dendritic cells [237,238,239,240,241,242,243,244]. AHR expressed in CD11c^+^ cells modulates the development of intestinal epithelium and intestinal immunity [245] thus, in turn it may modulate gut microbiota composition. Expression of AHR is attenuated in GF mice and AHR seems to mediate the impact of microbiota on the host’s metabolic parameters. Hence, AHR has been suggested to act as a mediator in the communication between the host and its gut microbiota [246]. The most evident interaction between microbiota and AHR comes from a study on the caspase recruitment domain family member 9 (CARD9). CARD9 promotes recovery from colitis by stimulating interleukin (IL)-22 production. Hence, *Card9*^−/−^ mice are more susceptible to colitis. Interestingly, the *Card9*^−/−^ mice-specific microbiota fails to metabolize tryptophan into compounds acting as AHR ligands. Transfer of these bacteria to wild-type GF recipients increases their susceptibility to colitis. Intestinal inflammation in these transfer recipients is attenuated after inoculation of the mice with three *Lactobacillus* strains capable of metabolizing tryptophan or by treatment with an AHR agonist [247]. Thereby, host expression of genes like *Card9* affects the microbiota pool and this regulation feeds back on the host’s health-status.

In the liver, PPARα and AHR affect the expression of a similar set of genes including *Cd36*, *Hmgcs2* [246,248], *Cyp2B*, *Cyp3A*, *Cyp2C11* [249] and *Fgf21* [65,250,251], indicating that there is an interplay between these two factors, which has been investigated [249,252,253,254]. FGF21 has been identified as the mediator of the metabolic benefit of AHR [251]. In turn, the impact of FGF21 on metabolism is partly mediated by PPARγ [255] creating a feed-forward loop as PPARγ also induces FGF21 expression [256]. FGF21 stimulates the expression of genes involved in lipid oxidation, ketogenesis and gluconeogenesis [65,257,258]. FGF21 improves insulin and leptin sensitivity and lowers blood glucose levels by activating glucose uptake in adipocytes. Its administration results in reduced body weight due to enhanced energy expenditure, fat oxidation and lipid excretion, while reducing hepatic *de novo* lipogenesis and hepatosteatosis [251,259,260]. Similarly to FGF15, the role of FGF21 in metabolism and body weight regulation seems to require mostly its central, rather than peripheral signaling [178,261,262]. Along these lines, microbiota-stimulated AHR may regulate metabolism via FGF21-mediated signaling to the CNS. Interestingly, in response to sugar consumption, hepatic FGF21 is released and attenuates sugar-seeking behavior by targeting the hypothalamic PVN [263,264]. Activation of FGF21 upon sugar uptake is mediated by hepatic PPARα and carbohydrate-responsive element-binding protein (ChREBP) [263]. Beside affecting metabolism, FGF21 regulates hydration by suppressing alcohol intake in favor of water consumption and this activity is mediated in part by SIM1-positive neurons in the hypothalamus [265].

AHR is widely expressed in the CNS [266,267] and plays there a multitude of roles in cell differentiation and maintenance. AHR-1, a *Caenorhabditis elegans* orthologue of AHR, is responsible for the development, orientation, and axonal migration of AHR-1-expressing neurons [268]. AHR-1 impacts neural cell fate determination in *C. elegans*, particularly GABAergic neurons [269]. Dioxin-activated AHR plays a role in CNS development in zebrafish [270] and mice [271,272]. However, expression of a constitutively active AHR in mice retards the development of interneurons in the olfactory bulb [273]. Furthermore, both deletion or activation of AHR alters adult hippocampal neurogenesis and impairs hippocampal-dependent contextual memory [274], suggesting a need for a balanced AHR activity. AHR has been shown to affect several aspects of behavior and neurodevelopmental illness in different study models [275,276]. AHR has been shown to decrease blood brain barrier (BBB) permeability [277,278], while BBB permeability is increased in GF mice [279]. However, the mechanism behind is not clear as AHR is capable of activating occludin and claudin 1 and 4 [280], but on the other hand it inhibits connexin 43 [281], which is essential for BBB integrity [282]. This is probably another example of the dual role of AHR, which again underlines the importance of a balanced activation of AHR. Moreover, AHR mediates anti-inflammatory effects on dendritic cells [283]. Ligand-activated microglial AHR exerts bi-directional effects on the regulation of LPS-induced neuroinflammation as it can promote or inhibit inflammation [284,285]. Notably, in patients with multiple sclerosis circulating levels of AHR agonists are decreased [286].

The effects of AHR expressed in CNS can be modulated by the microbiota [286]. Astrocytes are the most abundant glial cell population in the CNS, participating in diverse functions including control of the BBB, regulation of metabolism, neuronal transmission, CNS development and inflammation [287]. In experimental autoimmune encephalomyelitis (EAE), type 1 interferon (IFN-I) signaling in astrocytes reduces disease scores and inflammation via AHR and the suppressor of cytokine signaling 2 (SOCS2). EAE scores in mice increase following ampicillin treatment, whereas dietary supplementation with the microbial metabolites of tryptophan (indoxyl-3-sulfate, indole-3-propionic acid and indole-3-aldehyde)—which as mentioned above are AHR ligands—or delivery of the bacterial enzyme tryptophanase reduces CNS inflammation in the antibiotic treated mice. Thus, IFN-Is produced in the CNS functions in combination with bacterial metabolites of dietary tryptophan to activate AHR signaling in astrocytes and suppress CNS inflammation [286].

#### 3.4.2. Pregnane X Receptor

Pregnane X receptor (PXR, NR1I2) also known as the steroid and xenobiotic sensing nuclear receptor (*SXR*) is a nuclear transcription factor often referred as the master regulator of xenobiotic defense. PXR is involved in energy [288,289] as well as xenobiotic metabolism and it affects expression of phase I and phase II enzymes as well as phase III transporters [290]. PXR is expressed in barrier and excretory tissues, including intestine, liver, kidney and several regions of the brain [290,291,292] and it is synthetized as various splice variants in different organs [292,293,294]. In the liver, the function of PXR overlaps with that of FXR by regulating expression of a similar set of CYP proteins involved in both detoxification and BA metabolism. Additionally, other PXR target genes that are involved in xenobiotic metabolism also participate in BA metabolism [295]. In primary human hepatocytes, among other factors PXR regulates AHR and its target genes *Cyp1A1* and *Cyp1A2* [296]. Similarly to AHR, PXR is activated by microbial-specific metabolites of dietary tryptophan, and the indole derivative, indole 3-propionate (IPA) [297], which is produced by *Clostridium sporogenes* [52]. The ligand binding pocket of PXR has a relatively large volume capable of accommodating a variety of compounds. Moreover, PXR has the capability to expand its binding pocket to allow for larger compounds thus, various ligands are capable of its activation [298].

Secondary BAs, lithocholic acid (LCA) and its 3-keto metabolite (3-keto LCA), formed in the intestine by bacteria are ligands of mouse and human PXR [295,299]. LCA is a particularly toxic BA that causes cholestasis, a disease characterized by the impairment of bile flow and the accumulation of BA and biliary toxins in the liver and serum [300]. Moreover, pregnenolone 16α-carbonitrile (PCN), a ligand of PXR, blocks the hepatotoxicity and mortality caused by LCA exposure in rats [299,301]. Therefore, PXR, among other ligands, serves as a receptor of BA and upon activation coordinates the xenobiotic response in the detoxification of BAs.

Single nucleotide polymorphisms in *Pxr* are associated with increased susceptibility for developing IBD, Crohn’s disease and ulcerative colitis [302]. While PXR-deficient mice exhibit “leaky” intestine symptoms [297], ligand-activated PXR prevents leaky gut and maintains the intestinal epithelial barrier by stimulating the synthesis of junctional proteins, antagonizing TNF-α and TLR4, inhibiting the cytokine-induced myosin light chain kinase (MLCK), and activating the c-Jun N-terminal kinase½ (JNK1/2) [291,297,303,304]. Rifaximin, an antimicrobial agent used in the treatment of IBD, mediates its effects by increasing the expression of PXR. It has been shown to abrogate the DNA binding of NF-κB caused by LPS, to reduce mRNA levels of IL-8, RANTES, MIP-3α and TNFα [305]. Chrysin, a naturally occurring flavonoid with anti-inflammation activity, prevents DSS-induced colitis, an effect largely mediated by PXR [306]. Furthermore, the above mentioned PXR agonist, PCN, attenuates the intestinal barrier dysfunction observed after DSS administration in mice [304].

Absence of PXR is associated with anxiety-like behavior and recognition memory impairment in adult mice [307]. PXR knockout (KO) mice show decreased expression of tight junction protein ZO1 in the cortex and cortical microvessels [307]. In another study, activation of hPXR in mice in vivo tightened the BBB, reducing the delivery and central effect of administrated drugs. This effect is mediated by PXR-stimulated upregulation of P-glycoprotein, an ATP-driven drug export pump at the BBB [308]. P-glycoprotein is the most prominent drug efflux transporter at the BBB and plays an essential role in barrier function. Hence, most likely, similarly to AHR, microbiota derived-ligands regulate PXR activity in CNS, thus affecting the BBB.

#### 3.4.3. Constitutive Androstane Nuclear Receptor (CAR)

CAR (NR1I3) or constitutive androstane nuclear receptor, alike AHR and PXR, serves as a xenosenor. CAR expression level is affected by various factors. It is induced on the level of expression or activity by glucocorticoids (GC) [309], AHR [310], peroxisome proliferator-activated receptor γ coactivator-1α (PGC-1α) [311] and repressed by factors like epidermal growth factor (EGF) [312], TLR-2 signaling [313] and IL-1β [314]. Beside xenobiotics, CAR can be regulated by many endobiotics, including many steroids (androstanes, estrogens, and progestins) [315,316]. Importantly, expression of CAR in the intestine and in the liver depends on the presence of the microbiota [313,317]. CAR was originally characterized as a transcriptional regulator whose activation is stimulated by phenobarbital (PB) to stimulate the expression of *CYP*2*B* genes [318,319]. By now, the list of both the activators and target genes of CAR has significantly expanded and shows a high overlap with the corresponding list for PXR. These two NRs are often described together as both are expressed in organs like the small intestine, colon and liver. They both react to chemical stresses and are crucial to initial ligand recognition followed by subsequent jump-starting of xenobiotic metabolism. They work together to ensure homeostasis of cholesterol, BAs, sterols, lipids, heme and other endogenous hydrophobic molecules. Furthermore, the different splice variants of CAR, similarly to those of PXR, result in a number of deleterious or altered effects on gene transactivation, coregulator recruitment, as well as ligand binding [296,320,321,322,323]. Although CAR is less promiscuous than PXR, the smaller pocket size of CAR creates a more stable ligand-bound complex than the one of PXR, resulting in better packing of the AF-2 helix in the active conformation [324]. However, CAR is unique, relative to other NRs, in its ability to maintain a constitutive activity in the absence of a bound ligand. This ability is also referred to as an indirect activation, the exact mechanism of which remains to be elucidated [325,326]. In its inactive form, CAR, just like PXR, is bound to heat shock protein 90 (Hsp90) in the cytoplasm [327]. Upon activation and heterodimerization with the retinoid X receptor (RXR) it stimulates target gene expression in the cell nucleus [328]. A notable feature recently highlighted for CAR is that upon ligand binding it can also undergo translocation to cell membrane, which could imply additional non-genomic actions [329].

Notably, because CAR and PXR can be activated by the same ligands, upregulation of overlapping sets of genes allows for coordinated clearance and detoxification of harmful compounds [296,330]. Importantly, some drugs inhibiting CAR are agonists of PXR, thus allowing detoxification to take place even in the absence of one of the sensors. Together, the two NRs regulate expression of phase I enzymes of xenobiotics metabolism including CYP3As [331,332], CYP2Bs [319,332,333,334], and CYP2Cs [332,335,336]. They are capable of regulating approximately 90% of all phase II metabolic enzymes as both are involved in the transcriptional control of UDP-glycosyltransferases (UGTs), sulfotransferases (SULTs), glutathione-*S*-transferases (GSTs), acetyltransferases [324,337] as well as the drug transporters OATP1 and MDR1 involved in phase III detoxification [332].

CAR has also been linked to energy metabolism as its activation results in inhibition of lipogenesis, FA synthesis, and gluconeogenesis, as well as the increase of energy expenditure in BAT [338]. Long-term CAR activation improves glucose homeostasis and insulin sensitivity [339]. These effects are achieved by suppressing the expression of phosphoenolpyruvate carboxykinase (PEPCK), glucose-6-phosphatase (G6Pase) [339,340], and sterol regulatory element-binding protein 1c (SREBP1c) [341]. A very similar phenotype has been associated with PXR activation [288,289,342]. Moreover, fasting and caloric restriction induce CAR expression, which in turn coordinates an adaptive response by slowing down energy expenditure. Consequently, CAR KO animals are unable to accomplish the metabolic adjustment and lose more weight during caloric restriction interventions [343].

In the intestine, *CAR* gene expression is reduced in intestinal mucosal biopsies obtained from patients suffering from Crohn’s disease or ulcerative colitis with active mild or moderate inflammation, as well as in tissues isolated from colitic mice. Selective activation of CAR enhances wound healing in IEC monolayers in culture, an effect driven by enhanced cell migration. Finally, inhibition of CAR delays mucosal healing after the induction of experimental colitis, while its pharmacological activation accelerates recovery [344]. Thus, by affecting colonic health, CAR alters the natural milieu of the microbiota.

Notably, as shown by using null mice, CAR affects structural factors in the brain and the behavior of mice. These CAR-null animals show impairment in recognition memory and increased anxiety-like behavior, which is accompanied by neuronal and structural defects in the hippocampus and cortical area. Moreover, expression of the tight junction protein ZO-1 is reduced in cortical and hippocampal microvessels of the animals. Accordingly, peripheral injection of the neurotoxin kainic acid provokes a rapid onset of convulsions in CAR-null mice as compared to WT mice, indicating increased vascular permeability and susceptibility of these mice to neurotoxins [345].

In conclusion, gut flora modulates CAR activity, and CAR feeds back by reducing and promoting gut inflammation and healing, respectively. There is no evidence of direct gut–brain communication involving CAR. However, the CAR–microbiota interaction in gut, CAR’s role in the brain, and the importance of other xenobiotic receptors in gut–brain signaling provide a basis to speculate on a potential CAR-mediated signaling between the GI tract and CNS.

**Figure 2:**
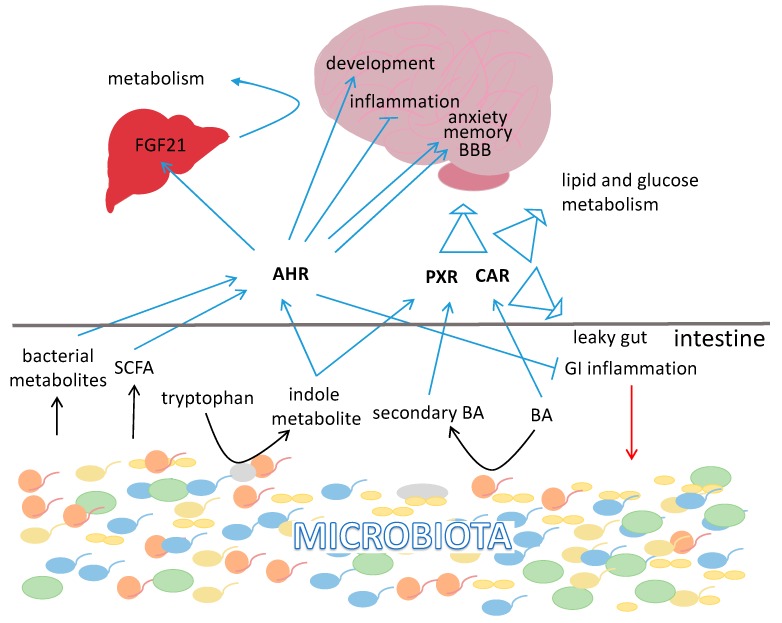
Xenobiotic receptors in the gut-brain axis. Bacterial metabolites, mainly indole and bile acids (BA) serve as ligands for xenobiotic receptors. These NRs play multifold roles in the brain by affecting the CNS development, permeability of the blood-brain barrier (BBB), memory and anxiety. Broad blue arrows illustrate the action of both PXR (pregnane X receptor) and CAR (constitutive androstane nuclear receptor), narrow blue arrows the actions of single factors, black arrows indicate bacteria-derived signals and the red arrow the impact of GI inflammation on the microbiota.

It seems that all of the xenobiotic receptors–AHR, PXR and CAR—interact with microbiota, and are required for the proper functioning of the brain (Figure 2) [346,347]. Importantly, these NRs are interconnected and show high level of coordination, and thus future research may show that they embody an essential pathway in gut–brain communication.

### 3.5. Steroid Receptors

#### 3.5.1. Glucocorticoid Receptor

The main steroids of the adrenal cortex are mineralocorticoids (MCs), glucocorticoids (GCs in humans mainly cortisol and in rodents mainly corticosterone) and the adrenal androgens. GCs are synthesized and released as a result of stress, but also in a daily oscillating manner [348]. The circadian release of GC can be maintained regardless of sleep pattern, light stimulation and rhythm of caloric intake [349,350]. Coordinated release of GCs, mineralocorticoids and catecholamines by the adrenal glands in response to stress is governed by the hypothalamic–pituitary–adrenal (HPA) axis, also referred to as the “stress axis”. Signals such as daylight promote the release of corticotrophin-releasing hormone (CRH) from the hypothalamus. CRH then stimulates the production of the adrenocorticotropic hormone (ACTH) in the pituitary, which in turn, initiates release of GC from the adrenal cortex to blood. As GC levels rise, a negative feedback loop is engaged that reduces both CRH and ACTH expression and secretion. This feedback mechanism regulates the magnitude and duration of GC release [351].

Although endogenous GCs are predominantly produced by the adrenal glands, there is evidence for extraadrenal GCs synthesis in the skin [352], thymus [353,354], vascular wall [355], lungs [356], brain [357] as well as IEC [358]. More specifically, liver receptor homologue-1 (LRH-1) in the cells of the crypt region of the intestinal epithelial layer promotes the expression of steroidogenic enzymes and the synthesis of corticosterone [358]. Additionally, immune stress and T cell activation are also stimulators of intestinal expression of the steroidogenic enzymes required for GC synthesis [359]. Importantly, the microbiota contributes to the production of corticosterone in the intestine [75] and influences the intracellular activity of GCs [360].

GCs are essential primary stress response hormones necessary for regulating numerous physiological processes in order to maintain homeostasis, and they reduce inflammation. Overall, their major effect on glucose homeostasis is to secure plasma glucose levels for brain during stress [361]. However, GCs also mediate multiple other physiological processes, including metabolic homeostasis, skeletal growth, cardiovascular function, reproduction, and cognition [362,363,364]. Synthetic derivatives of these hormones serve in the clinics as a standard treatment for inflammatory diseases, autoimmune disorders and hematologic cancers. Depending on the concentration of GCs, different receptor responses are triggered. Basal plasma levels of GCs are mediated via the mineralocorticoid receptors (MRs, NR3C2) (see below), while elevated, stress or circadian rhythm-triggered GCs activate the glucocorticoid receptors (GRs, NR3C1) [365,366]. The GR is expressed in almost every cell in the body and in multiple brain regions including the cerebral cortex, olfactory cortex, hippocampal formation, amygdala, septal region, dorsal thalamus, hypothalamus, trapezoid body, cerebellar cortex, locus coeruleus and dorsal raphe nucleus [367,368]. GR is encoded in a single gene, however, there are multiple GR protein isoforms. Alternative splicing of the 3′-end of the GR pre-mRNA produces GRα, GRβ and GRγ [369,370,371]. Moreover, alternative translation initiation from the single GRα mRNA transcript produces an additional eight diverse GRα proteins [372]. This substantial group of functionally distinct receptor subtypes undergoes various posttranslational modifications that further diversifies their signaling properties [373,374,375]. In the cytoplasm, GC-activated GR is released from a multiprotein complex composed of HSPs and immunophilins of the FK506 family [376,377] then translocates to the cell nucleus and modulates gene transcription via the following three mechanisms: GC receptor response element (GRE)-mediated direct transactivation; inverted repeat GREs (IR nGREs)-mediated direct transrepression; and tethered indirect transrepression [378,379,380,381,382,383,384]. The capacity of the GR to function as a transcriptional activator or repressor is determined by several polymorphisms in the GR gene [385]. GR can also modulate mRNA splicing [386] and mRNA stability [387] as well as microRNA expression and processing [388]. MR and GR can create homodimers or heterodimers acquiring new transcriptional properties [389,390]. Moreover, GR exhibits nongenomic signaling by direct protein–protein interactions that result in rapid cellular responses occurring within a few seconds to minutes, which involve the signaling cascade of various kinases and do not require changes in gene expression [380,391,392,393]. The various GR forms and their signaling modes generate pleiotropic effects in distinct tissues [394,395].

GCs and circadian rhythm factors are reciprocally regulated. The central circadian clock in the suprachiasmatic nucleus (SCN) of the hypothalamus entrained by light received by the retina orchestrates diurnal rhythmic oscillation of circulating GCs by influencing the activity of the HPA axis [396,397]. Additionally, the circadian rhythm core transcription factor heterodimer Clock-Bmal1 acetylates GR in peripheral tissues and represses its transcriptional activity in a circadian pattern that is opposite to the GR activation pattern by the HPA axis [398]. In return, the HPA axis, through the GR, drives circadian and ultradian bursts of transcriptional activity of the circadian rhythm regulators (Per 1, DBP) and adjusts the rhythmicity of the peripheral clocks in response to stressors [396,399]. Thus, the circadian rhythm of the peripheral clocks may become phase-shifted by GCs. Disruption of GR expression periodicity is associated with disease states characterized by GC resistance or sensitivity [400]. Moreover, cortisol-driven circadian and ultradian patterns of transcriptional activity of the *Clock* and *Per* genes is disrupted in major depressive disorders, bipolar disorder, and stress-related GI and immune disorders [401]. Furthermore, intestinal processes including nutrient absorption, cell proliferation, intestinal motility, metabolic activities as well as intestinal GC production are rhythmically regulated in a circadian manner [75,402]. Clock genes are expressed along the GI tract, their mRNA levels increase from the duodenum onwards and show highest levels in the colon. Similar to other organs, the clock genes in the jejunum and colon show a circadian rhythm of expression [403,404,405]. Moreover, the intestinal microbiota exhibits endogenous circadian rhythmicity that is partly synchronized with the host’s dietary rhythm [406,407,408]. Remarkably, gut microbiota-derived MAMPs, acting via TLR, regulate circadian clock genes and PPARα and thereby modulate the production of intestinal corticosterone [75]. Moreover, microbial cycles disturbed by a high-fat diet results in disruption of the central and liver clocks [407].

GR regulates multiple systems including the nervous, cardiovascular, musculoskeletal, immune, respiratory, reproductive, adipose and hepatic systems. It adjusts blood glucose levels by stimulating hepatic gluconeogenesis via direct induction of the *PEPCK* gene [409]. It suppresses bone formation by a number of mechanisms, including reduction of osteoblast differentiation [410], induction of osteoblast apoptosis [411], and stimulation of bone resorbing osteoclasts [412]. In adipocytes, it increases adipogenesis, alters adipokine production, reduces glucose metabolism and lipogenesis under basal or fasted conditions, while it stimulates lipogenesis in the fed state [413]. GCs extinguish inflammation and this is in part executed by GR’s ability to repress pro-inflammatory gene expression [414]. Numerous transcriptional targets of GR inhibit cytokine release, and GR itself has the capacity to transrepress NF-κB activity [382,383,415] making GR activation a powerful way to resolve inflammation. For that reason, dexamethasone (DEX), a synthetic ligand of GR, has been routinely used as an anti-inflammatory, immunosuppressive agent in the treatment of IBD [416]. Chronic exposure to stress and frequent GC release results in various adverse side effects such as osteoporosis, central adiposity, diabetes, hypertension, dyslipidemia, GI diseases and neurodegeneration [417,418,419,420]. Stress is also one of the most significant risk factors for development of irritable bowel syndrome [421,422,423]. Furthermore, a deficient or blunted HPA axis is commonly observed in the clinic in a wide range of autoimmune and inflammatory diseases [424,425,426].

In the intestine, GCs impact nutrient absorption by modulating expression of digesting enzymes [427], and enhance glucose uptake by stimulating gene expression of glucose transporters [428,429]. In coordination with the microbiota, they regulate gut ontogeny [427], play a role in the differentiation of the IEC, regulate the expression of tight junction proteins and thus, maintain the intestinal epithelial barrier [430,431]. GR is capable of antagonizing TNF-α-induced increase in MLCK protein expression, a key process mediating the TNF-α increase in intestinal tight junction permeability during inflammation [430]. Thus, GCs released during stress may have a gastroprotective action [432]. It has been shown that creating a lesion in the hypothalamic PVN or administration of antiserum of ACTH inhibits increases in corticosterone and induces gastric erosions provoked by cold-restraint as well as water and immersion-restraint stress; corticosterone replacement was able to prevent these malfunctions [432].

In the CNS, GR is implicated in both short- and long-term adaptations in response to stressors and participates in brain structural and physiological changes after stress exposure [433,434,435]. GR regulates the expression of genes related to neuronal and glial metabolism, neuronal plasticity and neurotransmission, and mediates gap junction intercellular communication in neural progenitor cells [436,437,438]. Stress-activated GR in microglia triggers changes in cell morphology and increases the production and release of inflammatory cytokines, switching from brain surveillance function to eliciting unfavorable effects on neural function and behavior [439,440,441]. Chronic stress and prolonged GC exposure have profound effects on emotion and cognition. It leads to maladaptive neuronal and glial plasticity, changes brain microglia morphology resulting in neuropsychiatric disorders such as affective, behavioral, and cognitive syndromes including depression, mood imbalance, increased fear conditioning and anxiety [433,441,442,443,444,445]. Adult rats raised by interactive mothers with high licking and grooming behavior, compared animals fed and groomed by less interactive females, show increased hippocampal GR expression, reduced HPA activation and less fear behaviors in response to acute stress [446,447,448]. Over-activity of the HPA axis is characterized by elevated levels of cortisol and disruption of GCs’ negative-feedback and is commonly associated with depression and post-traumatic stress disorder (PTSD) [449,450]. Methylation of the GR gene promoter, which impacts circulating cortisol levels, is negatively associated with severity of PTSD and correlates with specific responses to stress [451]. Moreover, individuals with major mental illness, who often exhibit hypercortisolemia, may have downregulated levels of GR mRNA. For instance, GR mRNA levels are reduced in parts of the cortex of patients suffering from depression, bipolar disorder, and schizophrenia. Furthermore, schizophrenia and bipolar disorder are associated with low GR mRNA levels in parts of the hippocampus [452].

Several studies indicate an impact of stress, GCs and GR on the gut microbiota. Different types of psychological stressors including chronic social defeat, restraint conditions, crowding, heat stress, and acoustic stress can alter the composition of the GI flora [453,454,455,456,457,458]. Stress induces gut permeability enabling bacteria and bacterial antigens to cross the epithelial barrier, which can activate a mucosal immune response with impact on the microbiota composition [19,20,21]. Importantly, in lean female mice, stress exposure modifies the microbiota composition to resemble that of obese mice [459]. Maternal separation of rat pups elicits alteration of the fecal microbiota, intestinal dysbiosis, and alters the HPA axis and colonic cholinergic neural regulation [18,456]. Early life stress increases visceral sensation, plasma corticosterone and systemic immune response in the stressed animals after an LPS challenge [18]. Exposure to DEX both acutely (10 days) or chronically (over 4 weeks) in mice leads to substantial shifts in gut microbiota accompanied by a significant downregulation of colonic *mucin 2* gene expression. Importantly, DEX treatment affects mucin expression only in specific-pathogen-free (SPF) but not GF mice. Thus, DEX alone is not sufficient to regulate mucin synthesis in the absence of gut microbes [460]. Moreover, a single high-dose injection of DEX increased the number of ileal anaerobic bacteria, while low-dose GC resulted in an increase in coliform bacteria [461]. These alterations in gut bacteria regulate the inflammatory state in a genetically susceptible mouse model of colitis, demonstrating a gut microbe-mediated mechanism by which GCs exert their anti-inflammatory effects in the colon. Thus, GC signaling partly mediates its effects through gut microbes.

Reciprocally, the gut microbiota also mediates stress responses, which is supported by the fact that an altered gut microbiota can lead to substantial shifts in the HPA axis response to stress [462]. Along a similar line of thought, GF mice present both a reduced anxiety phenotype and disturbed hypothalamic–pituitary–adrenal (HPA) axis [8,41,42,43]. However, in GF mice a mild restraint stress induces an excessive HPA response and higher plasma ACTH and corticosterone levels compared to the SPF controls, which can be partially reversed by colonizing the GF mice with the flora from SPF animals or with only *Bifidobacterium infantis* [43]. In contrast, reconstitution with the enteropathogenic *Escherichia coli*, but not with its mutant strain deprived of the translocated intimin receptor gene, enhances the response to stress even further, implying that commensal microbiota can affect the postnatal development of the HPA stress response in mice [43]. In another study using the forced swim test, 28 day long administration of *Lactobacillus rhamnosus* (JB-1) produced animals with lower levels of stress-induced corticosterone and reduced depressive and anxious behavior phenotypes [45]. This outcome was abolished with vagotomy, demonstrating that functional gut to brain direct nerve signaling is necessary for mediating stress response [45]. The HPA axis response to acute stress can also be attenuated by treatment with *Lactobacillus farciminis* [463]. Administration of a probiotic combination of *L. helveticus R0052* and *B. longum R0175* reduced anxiety-like behavior in rats and alleviated psychological distress in volunteers [44]. Moreover, administration of the prebiotics fructooligosaccharide (FOS) and galactooligosaccharide (GOS) abolished the corticosterone response to acute swim stress, attenuated anxiety related behavior in naive mice, and anxiety parameters in chronic social defeat [464]. Delivery of two strains of *Lactobacillus* species to mice pups ameliorated maternal separation-induced gut functional abnormalities and reduced bacterial adhesion and penetration into the colonic mucosa. Moreover, probiotic treatment improved gut dysfunction induced by maternal separation, at least in part by reducing the elevated corticosterone levels and by normalizing the activity of the HPA axis [465]. Thus, adequate stress response of the host depends on microbiota-regulated metabolism of GCs.

#### 3.5.2. Mineralocorticoid Receptor

The MR shows an affinity for MCs like aldosterone, its precursor deoxycorticosterone, as well as for GCs. MCs are a class of steroids produced in the adrenal cortex which mainly influence electrolyte and fluid balance, hemodynamic homeostasis and tissue repair. MR is expressed in many tissues such as the kidney, colon, heart, CNS, adipose tissue, macrophages and sweat glands [466]. In the brain, MR is largely restricted to neurons in limbic areas such as the hippocampus and amygdala [368,467,468]. This contrasts with the GR, which is expressed throughout the brain [368]. The hormones estrogen [469] and progesterone [470], and the nuclear receptors GR, MR, and their MR:GR heterodimer can activate *MR* gene transcription [471]. The promoter of the *MR* gene also contains a noncanonical cAMP-responsive element, which for instance allows GPR48 stimulation of MR expression through the cAMP/PKA pathway [472]. The *MR* gene can generate several transcripts [473], which in humans are derived from two different promoters [471,474]. GR and MR are co-expressed in many types of tissues, often in the same cells, where they interact at the molecular and functional levels, in synergy, but sometimes also in antagonism. GR and MR can coordinate their actions, for example in regulating neuronal Ca^2+^ voltage-gated currents in the hippocampus [475,476,477]. In preadipocytes, selective MR activation results in the transcription of several genes, including *Pparγ* required for preadipocyte maturation. On the contrary, GR agonists prevent adipogenesis [478,479]. Moreover, in response to inflammation GR often dampens MR’s effects. Overstimulation of MR in the heart, vessels, kidneys, and parts of the brain may lead to increased reactive oxygen species, inflammation, and cardiovascular and renal diseases [480]. Thus, balanced MR and GR activities are crucial for adaptation to stimuli and homeostasis [481,482]. The DNA binding domains of MR and GR are nearly identical, and both receptors are capable of binding GREs as well as nGREs [483,484]. Besides competing for the same ligands MR and GR share several co-regulatory proteins required for the initiation of transcription. However, the primary functions of MR and GR and some of their ligands serve different purposes and are regulated very differently [485,486]. A fundamental role of MR includes the regulation of ion and fluid transport in order to sustain osmotic and hemodynamic homeostasis, maintenance of membrane excitability in neurons and muscle cells as well as responses to injury. MR regulates blood pressure, and its inappropriate activation leads to hypertension [487,488,489]. Treatment with MR antagonists decreases both cardiovascular risk markers and insulin resistance [487,488,489,490]. Accordingly, bacterially-derived GC metabolites have been suggested to cause hypertension [360]. Besides acting as a typical NR and modulating gene expression, MR displays several rapid nongenomic activities which do not require transcription and are mediated by classical cell signaling pathways [491,492,493,494]. The types of nongenomic signaling mediated by MR vary between different cells and depend on the activating stimuli. Typically, it includes protein kinase C (PKC), cyclic adenosine 3′,5′-monophosphate (cAMP) and phosphoinositide 3-kinases (PI3K), cumulating with downstream activation of various kinases, ion channels and pumps [491,492,493,494]. MR also acts through the EGF–EGFR–MEK1/2–ERK1/2 signaling cascade which influences cell differentiation, proliferation and repair [492,495,496]. In various tissues, nongenomic MR activation leads to intracellular Ca^2+^ flux [497].

MR exhibits different ligand preferences depending on the tissues: aldosterone in the kidney and colon epithelia, and GCs in most parts of the brain. In most tissues at basal levels, GCs favors binding to MR. For instance, in the hippocampus MRs bind GCs with approximately 10-fold higher affinity than its colocalized GR. When GCs levels rise due to stress or circadian cycle GCs also activate GR [366,368,498,499,500]. Thus, GR is thought to be responsible for the GCs-triggered stress response. However, MR located at the plasma membrane has been shown to also mediate stress level GC effects in a nongenomic manner in the hippocampus [501]. In the brain, GCs are more abundant than aldosterone, thus they constantly occupy MR. GCs are able to passively diffuse across the plasma membrane, and their bioavailability within the cell is controlled by two 11β-hydroxysteroid dehydrogenase enzymes. 11β-hydroxysteroid dehydrogenase-2 (11β-HSD2) converts cortisol and corticosterone to the inactive cortisone, and 11β-hydroxysteroid dehydrogenase-1 (11β-HSD1) converts cortisone back to its active form [502,503]. The two 11β-HSD enzymes show cell-specific expression, therefore controlling the level of active GCs and in turn receptor activation. 11β-HSD2 is expressed in aldosterone-sensitive tissues, including kidneys, liver, lungs, colon, salivary glands, H2D2 neurons and placenta, while 11β-HSD1 is highly expressed in key metabolic tissues like liver, adipose tissue, and CNS. Inactivation of GCs by 11β-HSD2 allows binding of aldosterone to the MR within aldosterone target cells, and at the same time, limits activation of the GR in these cells [504,505,506]. 11β-HSD1 plays an important role in switching between MR-mediated proinflammatory activation of IL-6, TNF-α and NF-κB and GR-triggered suppression of these factors in microglial cells [507]. Thus, this enzyme mediates activation of microglia and may affect the development of neurogenerative diseases. Remarkably, the microbiota is able to produce glycerrhetinic acid-like factors (GALFs), named after glycerrhetinic acid from licorice root, which can inhibit 11β-HSDs activity [360]. Therefore, by modulating the activity of the 11β-HSD enzymes, the microbiota influences whether MR or GR is activated.

Appropriate activation of the MR is essential for normal neuronal differentiation, migration, and function of the developing and adult brain [508,509,510,511]. Chronic social stress in adolescent mice evokes a decrease in the mRNA levels of MR and GR in the hippocampus. It also increases adrenal weight, elevates GCs levels, dampens the circadian rhythm and promotes anxiety-related behaviors compared to unstressed mice. The decrease in MR expression and behavioral changes are still detectable a year later, suggesting underlying epigenetic events [512]. Subjects suffering from depression and bipolar disorder display reduced total MR and increased ratio of MR to GR mRNA expression in the prefrontal and anterior cingulate cortex. On the contrary, a decreased MR to GR ratio for both mRNA and receptor is seen in the dorsolateral prefrontal cortex and PVN [513]. Moreover, chronic treatment with corticosterone triggers a depression-like phenotype in mice, however, co-administration of a MR-specific antagonist, spironolactone, mitigates depressive behavior [514]. Both MR and GR participate in corticosteroid-induced direct transcriptional repression of the *5-HT_1A_* gene [483]. Physiological effects of 5-HT_1A_ receptor agonists are attenuated after chronic treatment with GCs [515,516]. Therefore, both receptors influence behavior and impact depression [445,517,518].

The hippocampus, which shows the highest expression of MR in the brain, is the major integrative center for the formation of memories, learning, cognition, and coping with stress. In fact, MR activity is crucial for learning and for forming memories. MR antagonists boost cognition in heart failure patients, sometimes without an actual impact on cardiac function [519] while the decrease in MR expression in the aged hippocampus positively correlates with age-induced impairments in spatial memory [520]. However, for optimal cognitive performance, balanced activation of both MR and GR is required [521]. Interestingly, therapy with the antidepressant amitriptyline in young rats increases hippocampal MR but not GR expression, and stimulates spatial memory. The same treatment has no effect in aged rats [522]. Furthermore, long-term treatment with amitriptyline in middle-aged rats prevented the prevalence of cognitive impairments and reduced anxiety-related behaviors in aged rats [523]. In contrast, short-term use of MR antagonists in healthy humans has adverse effects on attention, memory, and cognition [524].

**Figure 3:**
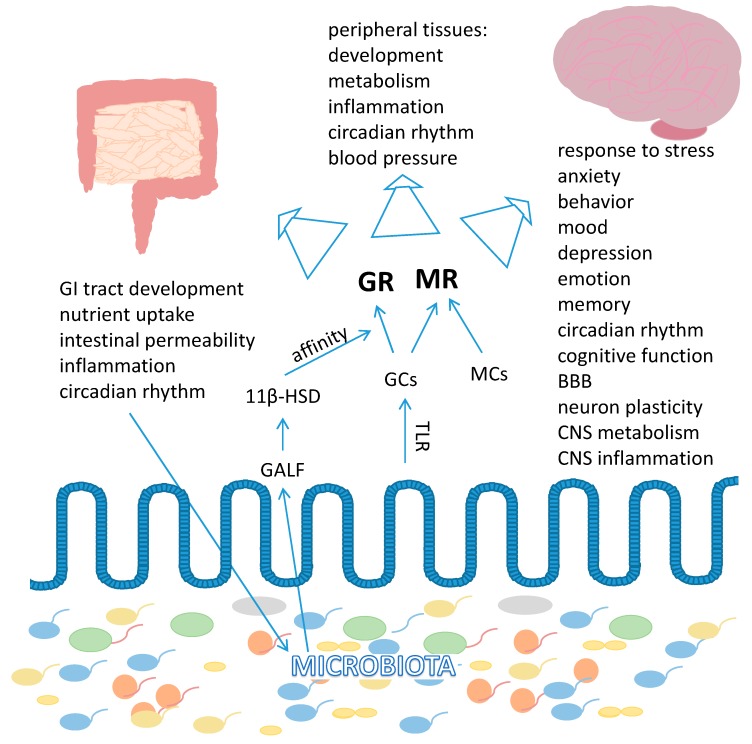
Interaction between glucocorticoid receptor (GR), mineralocorticoid receptor (MR) and microbiota. By regulating the activity of 11β-HSD, bacterial glycerrhetinic acid-like factors (GALFs) modulate GCs availability and GCs affinity towards GR or MR. Additionally, the microbiota regulates circadian production of GCs in the intestine thereby affecting the activity of steroid receptors in multiple tissues.

In summary, the microbiota modulates levels of GCs thereby affecting activity of GR and MR in multiple organs including the brain (Figure 3).

#### 3.5.3. Estrogen Receptors

Estrogens are primarily produced in the ovaries, adrenal glands and adipose tissue and are C-18 steroid hormones derived from the reduction of C-27 cholesterol. The main forms of endogenous estrogens are estradiol (E2), which is predominant in nonpregnant women prior to menopause; estrone (E1), present after menopause; and estriol (E3), abundant during pregnancy [525]. While passing through the liver E1 and E2 estrogens undergo irreversible hydroxylation at the C-2, C-4, or C-16 positions of the steroid ring. These modifications produce estrogen metabolites varying in hormone potency and half-life. The types of estrogen and their modifications influence their preference for and capacity to activate different types of estrogen receptors (ERs) [526].

Estrogens mainly play role in reproduction, however, they are also vital modulators of metabolism and behavior. They protect against high-fat diet induced insulin resistance and glucose intolerance in mice [527]. Lower levels of estrogens in postmenopausal women [528] or in ovariectomized animals are associated with obesity [529], while estradiol-17β replacement in ovariectomized mice prevents weight gain by diminishing food intake and stimulating energy expenditure [529]. Accordingly, hormone replacement therapy in postmenopausal women reverses the progression of obesity and metabolic dysfunctions [530]. Importantly, estrogen levels strongly impact gut microbiota composition and microbiota affects body weight. In men and postmenopausal women, levels of total urinary estrogens correlate to richness and α diversity of fecal microbiota [531,532]. Ovariectomy in adult mice diminishes gut microbiota richness [533]. In rats, neonatal androgenization or adult ovariectomy causes shifts in the relative abundances of the two major phyla causing a higher *Firmicutes* to *Bacteroidetes* ratio [533]. In humans, similar gender-dependent differences were observed. Estrogens are strongly and significantly associated with the fecal *Clostridia* taxa, including non-*Clostridiales* and three genera in the *Ruminococcaceae* family [531], while men have higher levels of *Bacteroidetes* and *Prevotella* than women [534,535]. In mice, sex differences in gut microbiota are decreased after castration of male mice [536]. Stunningly, hormonal changes caused by early androgenisation or ovariectomy impact the microbial flora more strongly than nutritional changes from chow to a high fat diet [533], suggesting a decisive role for gonadal hormone levels in the modulation of GI flora.

A cluster of bacteria, referred to as “estrobolome” due to its capacity to metabolize estrogens, can significantly influence the host’s estrogen levels [537]. The series of reductive reactions leading to the synthesis of estrogens from cholesterol is partly catalyzed by hydroxysteroid dehydrogenases (HSD), a group of alcohol oxidoreductases. Bioinformatic annotation of HSD in all entirely sequenced bacterial genomes has proven that HSDs are found in a wide variety of microorganisms including bacteria and archaea. A large number of HSD-expressing bacteria participate in the normal human gut microbiota [538].

After circulation in the blood, estrogens and their metabolites are directed to the liver where they are conjugated through glucuronidation or sulfonation which facilitates their excretion in the bile, urine and feces [539]. β-glucuronidases and β-glucosidases, expressed by several bacterial strains, are capable of deconjugating estrogen which can then be reabsorbed by the intestine and reenter the host’s estrogen pool [540,541]. The activity of fecal bacterial β-glucuronidase activity, just like the intestinal flora composition, can be modulated by diet [542,543,544]. In rats, a decrease in fecal β-glucuronidase enzyme activity was observed after antibiotics administration [545]. In line with this observation, antibiotic therapy results in significant increases in fecal conjugated estrogens and a reduction in urinary estrogen in men and women [546,547,548]. More than half of the low G + C% gram-positive *Firmicutes*, which are a dominant bacterial group within the human large intestine [549], harbor β-glucosidase activity [550]. Most of *Bacteroides* spp. carry high β-glucuronidase and β-glucosidase activity, while the majority of *Bifidobacterium* spp. show only β-glucuronidase activity. The highest β-glucuronidase and β-glucosidase activities in feces correlate with the occurrence of *Clostridium* spp. [540,550,551]. Similarly, *Peptococcus niger* by means of sulfatase activity can deconjugate estrogen sulfate metabolites [552]. Furthermore, the gut microbiota is able to metabolize estrogen-like compounds from nutrition. For instance, daidzein found in soy can be metabolized to O-desmethylangolesin (ODMA) and S-equol [553], which are structurally similar to estrogen and can activate ER [554,555]. It is noteworthy that individuals harboring a gut flora capable of producing ODMA, but not S-equol, are more likely to be obese [553]. Importantly, several epidemiologic studies have suggested a possible association of antibiotic use and breast cancer risk which may be a consequence of disturbed estrogen metabolism [556,557].

Microbiota-derived estrogen may influence the host’s fertility. In GF mice, reproduction capacity is lower than in SPF mice. Inoculation of GF mice with *B. distasonis* and *C. perfringens* induces significant increase in sperm motility in male mice while in females it results in the normalization of the estrous cycle, and rises the copulation and fertilized egg implantation rates. On the contrary, the reproduction rate of mice carrying only the single *Bacillus subtilis* strain is comparable to GF mice, underscoring the importance for hormonal homeostasis of selected bacterial strains in the gut [558].

Estrogen exerts its activity via the estrogen receptor α (ERα, NR3A1) and β (ERβ, NR3A2) [559,560]. ERα and ERβ are highly homologous, particularly in the DNA binding domains (95%) and the ligand binding domain (55%), and show affinity for the same estrogen response elements (ERE) in DNA [560]. Although ERα and ERβ share similar ligand binding domains, ERβ possesses a relative binding affinity for several steroid hormones that differs from that of ERα [561]. ERα and ERβ are expressed in multiple organs [562] including all parts of the GI tract [563,564,565,566], but their expression levels differs in various tissues. For instance, ERα is the predominant isotype in the breast and uterus, whereas ERβ is expressed at higher levels in the urogenital tract, endothelial cells and the CNS [562,567]. ERα and ERβ are present throughout the rostral–caudal extent of the brain and spinal cord and their expression overlaps in most of the CNS with a few exceptions. For instance, although both receptors are expressed by neurons in the arcuate nucleus and hippocampus, ERα is more abundant in the arcuate nucleus, and ERβ is more prevalent in the hippocampus [568,569,570,571,572]. Importantly, ERβ mediates brain development by regulating neural progenitor cell proliferation [573] and stem cell differentiation into dopamine neurons [574].

ERα, but not ERβ, is vital to reproductive function and regulation of the preovulatory surge of luteinizing hormone (LH) in response to rising levels of estrogen [575,576,577]. However, ERβ may be involved in mediating the negative feedback control of anterior pituitary LH secretion, a process mainly governed by ERα [578]. Moreover, ERα-null female mice are deficient in sexual behavioral interactions, they are more aggressive towards other females and show reduced levels of parental behavior towards newborn pups [575]. Thus, ERα regulates reproduction not only at the physiological, but also behavioral level.

The effects of estrogens on energy balance are primarily mediated by ERα, as women or female mice with mutations in the ERα gene display hyperadiposity [579,580]. Importantly, estrogen levels directly affect nutrient uptake from the intestine [581,582]. Female mice lacking ERα in hypothalamic steroidogenic factor-1 (SF1) neurons are characterized by hypometabolism and abdominal obesity, but not hyperphagia. In contrast, loss of ERα from the hypothalamic POMC neurons leads to hyperphagia, without directly influencing energy expenditure or fat distribution [583]. Thus, estrogens act on distinct hypothalamic ERα neurons to regulate different aspects of energy homeostasis and reproduction.

While ERα is essential for reproductive neuroendocrine function, ERβ seems more important for emotional behavior. ERβ-null mice show increases of 5-HT_1A_ receptor expression in the medial amygdala as well as lower serotonin and dopamine levels in several brain regions [584,585]. Accordingly, E2, acting via ERβ located in 5-HT cell bodies of the dorsal raphe nucleus, induces expression of tryptophan hydroxylase which is involved in the synthesis of 5-HT [586]. As a result, mice and rats treated with E2 or selective ERβ agonists and subsequently submitted to the forced swim test, spend less time immobile and struggle longer than non-treated controls, which is interpreted as a less depressive-like behavior [587,588]. This effect is not observed in ERβ-null mice [587]. Selective agonists of ERβ exert potent anxiolytic activity in rats [589,590], whereas ERβ-null mice show increased anxiety accompanied by reduced threshold for the induction of synaptic plasticity in the basolateral amygdala [584,585]. In elevated plus maze and open field tests for anxiety-like behaviors, ERβ-null mice show increased anxiety relative to their wild-type counterparts. Importantly, this phenotype of the ERβ-null mice is present only in females, indicating a specific gender-dependent sensitivity to hormonal changes expressed in behavior [584,585]. ERβ may exert its impact on behavior via the HPA axis [591] and other behavior-related hormones as it represses neuronal vasopressin [592], CRH [593] and corticosterone [590]. Notably, GCs are transported in the blood predominantly bound to corticosteroid-binding globulin (CBG), also known as transcortin, which serves as a regulator of the biological availability of GCs. CBG is produced by the liver, and interestingly, it is stimulated by estrogens, which is reflected as higher levels of total circulating corticosterone in females compared to males [594]. Importantly, the sex hormones estrogen and progesterone influence the expression of MR [469,470] and the ER inhibits the transactivational effects of the MR in several cell types [595]. In addition to MCs and GCs, progesterone is a competitive MR antagonist with a similar affinity for the MR as aldosterone [596]. Moreover, MR mRNA levels in the human hippocampus show higher concentrations in women compared to men [597]. The hormonal differences associated with MR correlate with occurrence of cardiovascular diseases in premenopausal woman. Consistently, sex differences exist in the cortisol stress response [598], in response to parental separation of pups [599], in hippocampal MR and GR expression after exposure to stress of adults rats [600,601] and in the prevalence of stress-related psychiatric disorders [602,603]. On the contrary, DEX-treated female rats increase ERβ expression in the hypothalamic PVN and supraoptic nuclei (SON) [604], whereas adrenalectomy reduces ERβ mRNA expression in the PVN, and corticosterone replacement fully reverses this effect in a dose-dependent fashion [605]. Thus, ERβ mediates and interacts with the HPA axis.

In contrast, treatment with the ERα agonist propylpyrazoletriol (PPT) was anxiogenic in the elevated plus maze test and, in a consistent manner, stimulated corticosterone stress hormone response [590,593]. These results provide an explanation to the fact that estrogen has both anxiogenic and anxiolytic effects [606,607].

Considering the vital contribution of estrogens to the regulation of fertility, metabolism and behavior described above, the regulation of estrogen bioavailability by the microbiota impacts essential functions in the host’s life.

### 3.6. Thyroid Hormone Receptors

An essential component of thyroid hormones (TH), iodide is actively transported and concentrated in the thyroid gland and incorporated in THs. Thyroid cells combine iodine and the amino acid tyrosine to make T_3_ and T_4_ [608,609,610,611]. Thyroxine (T_4_) contains four iodine atoms and the more potent triiodothyronine (T_3_) has three of them. T_4_ is the prevalent form of released THs and deiodination of its outer ring by enzymes iodothyronine deiodinases yields T_3_. The deiodinases are selenoproteins expressed in various tissues including the GI tract which regulate the intracellular biological activity of THs [612,613,614,615]. Thus, deficiency of deiodinases can mimic hypothyroidism.

TH secretion is regulated by a negative feedback system involving the hypothalamus, the pituitary gland and the thyroid gland. Thyrotropin releasing hormone (TRH) is synthetized in the hypothalamus and when released it is transported in the circulation and binds to receptors of the pituitary thyrotropes. The pituitary then secretes thyroid stimulating hormone (TSH) which promotes TH release from the thyroid gland. Both TRH and TSH secretions are negatively regulated by TH. Also, somatostatin and dopamine inhibit TSH release [616,617]. Importantly, both of these factors can be produced by microbiota [35,48,49].

THs serve as ligands for the thyroid hormone receptors α (TRα, NR1A1) and β (TRβ, NR1A2), which occur in several forms. TR-α1 is widely expressed with high levels in cardiac and skeletal muscles. In the brain its highest levels are in the olfactory bulb, hippocampus and parts of cerebellar cortex. TR-α2 is most abundant in the brain but it is the only TR form that is not capable of binding thyroid hormone. TR-β1 is present mainly in brain, liver and kidney and TR-β2 is limited to brain with high levels in the hypothalamic PVN and anterior pituitary [618,619]. TR can bind to DNA whether or not it is activated by appropriate ligands. In the unliganded inactivated from it recruits corepressors thereby inhibiting gene expression. Upon ligand binding, the TR changes conformation facilitating dissociation of the corepressors and the recruitment of coactivators resulting in the stimulation of target gene promoters and gene transcription [620]. TRs also exerts nongenomic actions [621].

T_3_ increases cardiac output and heart rate, lung ventilation, basal metabolic rate, catabolism of proteins and carbohydrates as well as growth and brain development. In the brain, THs promote a wide range of developmental processes including myelination, neuronal migration as well as neuronal and ganglion cell differentiation [611,619]. THs also affect mood and behavior [622]. Among target genes in the brain TH regulates genes coding for brain myelin basic protein [623,624], BDNF [625], glutamine synthase [626], prostaglandin D2 synthase [627], adhesion molecules, matrix proteins, factors affecting neuronal migration and apoptosis, nerve metabolism and signal transduction [628,629,630]. Both TH deficiency and excessive production lead to metabolic and neuronal malfunctions. Hyperthyroidism is characterized by increased levels of free circulating TH, weight loss, frequent bowel movements and associated diarrhea, weakness, tremors, difficulty to sleep, heat intolerance, increased heartbeat, anxiety, and memory problems. Hypothyroidism is associated with weight gain, exhaustion, constipation, indigestion, depression, anxiety, psychosis, general pains and fertility problems in females [619,631,632,633]. Hypothyroidism affects brain development and may cause neurological defects including diminished neuron development in hippocampus, cerebral cortex, visual and auditory cortex [628,634,635]. Hypothyroidism-caused developmental malfunctions can be reversed if THs are administrated within 2 weeks after birth [628]. On the contrary, absence of congenital hypothyroidism treatment in infants leads to a profound brain development delay accompanied by mental retardation. Interestingly, hypothyroid patients show lower amounts of gut *Bifidobacterium* and *Lactobacilli* while having more *Enterococci* than healthy subjects [636]. Additionally, patients with a history of hypothyroidism show more prevalent occurrence of small intestine bacterial overgrowth (SIBO) which may be reversed by antibiotic treatment [637].

Hypothyroidism may be due to genetic factors or dietary iodine deficiency. Iodine concentration in the thyroid gland as well as in peripheral tissues largely depends on ionic pumps and transporters, the efficiency of which is modulated by iodothyronine deiodinases as well as the availability of iodine and selenium. An inability to synthesize TH results in a decrease of the negative feedback to hypothalamus, which stimulates TSH production leading to enlargement of thyroid gland, a condition named goiter. An enlarged thyroid gland enhances its ability to trap iodine [638,639,640].

The amount of exchangeable T_3_ and T_4_ in the intestine constitutes the second largest reservoir of TH after the thyroid gland, before the liver and kidneys [641]. Short gut syndrome and bariatric surgery are both associated with reduced intestinal nutrient uptake and malnourishment, which do not influence iodine levels [642,643]. On the contrary, modulation of rat microbiota by kanamycin reduces iodine uptake [644] implying that the gut flora constitutes the functional component of the GI tract responsible for iodine absorption. Moreover, the microbiota in the small intestine, cecum, colon and feces of rats reversibly binds T_3_ and T_4_ and this capacity is abolished by antibiotic treatment [645,646]. Ex vivo studies show that *Escherichia coli*, one of major gut colonizers, is capable of binding T_3_ and T_4_ [647]. Thus, gut flora could serve as a reservoir, retaining and releasing TH.

Selenium is an essential microelement required for the synthesis and bioactivity of selenoproteins, including deiodinases. Consequently, this microelement affects the activity of TH. Selenium is absorbed in the duodenum and caecum [648] and in the body is most highly concentrated in the thyroid gland [649]. Gut bacteria also require selenium and when it is not taken up by the host, colonic bacteria utilize it [650]. Upon selenium shortage, the microbiota gets to the point of competing with the host for selenium [651], which may result in reduced expression of selenoproteins in the host [652] and the microbiota may inhibit the enzymatic activity of deiodinases in the cecum and colon walls [653]. Selenium supplementation results in increased diversity of gut bacterial population [652] and enhanced colonic and rectal fermentation [654] as multiple microbes are capable of producing their own selenoproteins [655,656,657,658].

THs are inactivated mainly in the liver by conjugation. Attached sulfate and glucuronic acid moieties enable secretion of the conjugated hormones in bile and decrease their reabsorption from the intestine. Moreover, sulfation facilitates irreversible deiodination and catabolism of THs [659]. However, glucuronidated and sulfated THs can be hydrolyzed to active precursors in the GI tract and various other tissues. Thus, conjugated THs can serve as a reservoir for active hormones. In rats, but not humans, stimulation of glucuronidation by drugs and toxins may results in a drop in T_4_ and T_3_ levels provoking elevated TSH secretion and resulting in goiter formation [638,660,661]. Incubation of various TH sulfate conjugates with human or rat feces results in complete hydrolysis of the conjugates. However, preheated fecal suspensions and supernatants of centrifuged fecal solution do not affect sulfation of TH. Similarly, fecal suspension obtained from GF or ampicillin-treated rats showed drastically reduced deconjugation capacity [662,663,664]. Consequently, in GF mice the enterohepatic cycle of TH including intestinal deconjugation of T_3_ secreted in bile and its reabsorption to circulation is abolished [665]. Deconjugation activity relies on the presence of obligate anaerobic intestinal bacteria. Bacterial strains isolated from human feces, including *Peptostreptococcus productus*, possess sulfatase activity [662,663,664].

The bacterial signaling that influences TH turnover and LPS which triggers septic shock also promotes non-thyroidal illness syndrome. Higher doses of LPS in mice decrease the levels of liver deiodinase mRNA, pituitary TR mRNA and protein, and reduces the amount of TSH receptor mRNA in the thyroid gland [666]. In pigs, LPS lowers TH levels in serum and most tissues. Moreover, it decreases the expression of deiodinases in the liver and kidneys as well as deiodinases activity in kidneys. LPS administration results in lowered mRNA levels of TRβ in the frontal lobe, adrenal glands and kidney cortex [667]. Thus, bacterial LPS is capable of affecting TH signaling on multiple levels in the whole organism, including in the brain.

Patients suffering from celiac disease, which is generally accompanied by GI inflammation and abnormal microbiota, show malabsorption of orally delivered T_4_ and in case of Hashimoto thyroiditis require higher doses of the T_4_ drugs [668]. Approximately one third of patients with autoimmune thyroid disease show also symptoms of atrophic gastritis [669]. Furthermore, 40% of Hashimoto thyroiditis patients exhibit lymphocytic colitis [670]. Rarefication and partial disappearance of microvilli, spaces between microvilli and thickening of microvilli were increased in intestinal mucosal biopsies from autoimmune thyroiditis patients. The patients also showed increased mucosal permeability [671]. Thus the host’s TH affects gut health and therefore also the gut flora. TH can also affect the bacteria population by influencing the number of parietal cells in the gastric mucosa, the secretion of pepsinogen and the basal acidity in the stomach [672,673]. Thereby in the stomach, TH increases the efficiency of killing bacteria taken up with the food, reducing the colonization of more distal parts of the GI tract by these foodborne microorganisms.

The direct connection of enteric bacteria and TR in the context of the gut–brain axis has not been addressed so far. However as seen above, the importance of TR in brain development and proper functioning and the crucial impact of the microbiota on TR ligands availability, provide strong support for a GI tract–CNS TR-dependent signaling.

## 4. Conclusions and Future Directions

**Figure 4:**
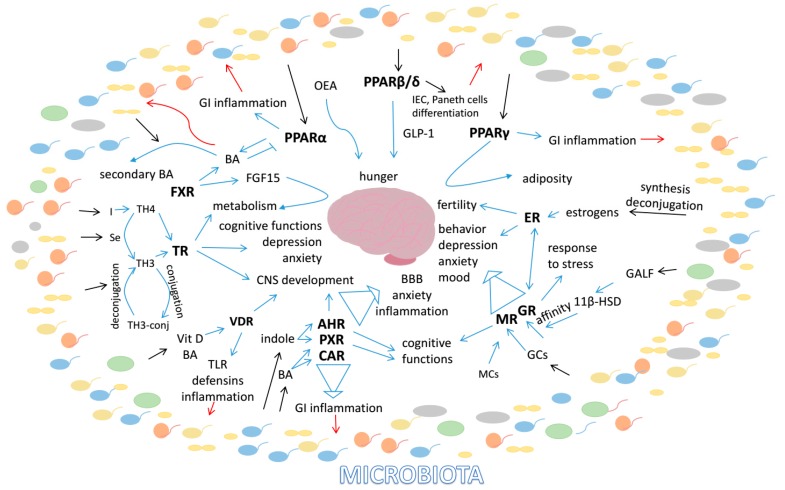
Summary of the interactions of NRs with enteric microbiota and the brain. Broad blue arrows indicate the involvement of groups of NRs (AHR/PXR/CAR; MR/GR), narrow blue arrows picture single factor actions. Black lines illustrate bacteria-born signals and red arrows specify impacts on the microbiota.

As presented in this review, there are multiple studies proving the connection between a healthy gut and a healthy brain. NRs are key players in regulating virtually every process necessary for the survival and health of a living organism. By influencing NRs, gut flora participates in the regulatory roles of the nuclear receptors (Figure 4). Via signaling to the brain, the microbiota regulates metabolism, CNS development, inflammation as well as mood and behavior. Notably, it also impacts reproductive behavior and, therefore, contributes to species survival. Importantly, the human host has the capacity to voluntarily influence and improve its own microbiota by the power of nutritional or probiotic interventions. Thus, nutritional approaches, combined with physical exercise, aiming at providing or sustaining the production of NR ligands and increasing the microbiota diversity should appear as a common sense approach to promote a physically and physiologically healthy life.

Multiple NRs have been proven to interact with the microbiota and/or to play a role in the brain. However, a full picture of the involvement of NRs in the gut–brain axis is still missing. Interestingly, there is one exception. The RAR-related orphan receptor (ROR) is involved in cerebellum development [674]. Additionally, the RORγ subtype is considered to be a master regulator of lineage specification of a subset of CD4^+^ T helper cells (T(H)17) playing a role in the development of autoimmune diseases [675]. Importantly, microbes induce Th17 cells via RORγ, which results in activation of sensory neurons [676]. Evidence for such a direct regulation of the nervous system by bacteria has been rarely found so far. Therefore, more research is needed to fully unveil the great potential of NRs in regulating the enteric microbiota–gut–brain axis and to learn how we could benefit from modulating their activity by pharmacological or nutritional interventions.

## References

[1] Evans R.M., Mangelsdorf D.J. (2014). Nuclear Receptors, RXR, and the Big Bang. Cell.

[2] Martin R., Makino H., Cetinyurek Yavuz A., Ben-Amor K., Roelofs M., Ishikawa E., Kubota H., Swinkels S., Sakai T., Oishi K. (2016). Early-Life Events, Including Mode of Delivery and Type of Feeding, Siblings and Gender, Shape the Developing Gut Microbiota. PLoS ONE.

[3] Yatsunenko T., Rey F.E., Manary M.J., Trehan I., Dominguez-Bello M.G., Contreras M., Magris M., Hidalgo G., Baldassano R.N., Anokhin A.P. (2012). Human gut microbiome viewed across age and geography. Nature.

[4] Ianiro G., Tilg H., Gasbarrini A. (2016). Antibiotics as deep modulators of gut microbiota: Between good and evil. Gut.

[5] Gupta V.K., Paul S., Dutta C. (2017). Geography, Ethnicity or Subsistence-Specific Variations in Human Microbiome Composition and Diversity. Front. Microbiol..

[6] Garcia-Gutierrez E., Mayer M.J., Cotter P.D., Narbad A. (2018). Gut microbiota as a source of novel antimicrobials. Gut Microbes.

[7] Rothschild D., Weissbrod O., Barkan E., Kurilshikov A., Korem T., Zeevi D., Costea P.I., Godneva A., Kalka I.N., Bar N. (2018). Environment dominates over host genetics in shaping human gut microbiota. Nature.

[8] Diaz Heijtz R., Wang S., Anuar F., Qian Y., Bjorkholm B., Samuelsson A., Hibberd M.L., Forssberg H., Pettersson S. (2011). Normal gut microbiota modulates brain development and behavior. Proc. Natl. Acad. Sci. USA.

[9] Jenkins T.A., Nguyen J.C., Polglaze K.E., Bertrand P.P. (2016). Influence of Tryptophan and Serotonin on Mood and Cognition with a Possible Role of the Gut-Brain Axis. Nutrients.

[10] Schmidt C. (2015). Mental health: Thinking from the gut. Nature.

[11] Smith P.A. (2015). The tantalizing links between gut microbes and the brain. Nature.

[12] Mayer E.A., Knight R., Mazmanian S.K., Cryan J.F., Tillisch K. (2014). Gut microbes and the brain: Paradigm shift in neuroscience. J. Neurosci..

[13] Sommer F., Backhed F. (2013). The gut microbiota—Masters of host development and physiology. Nat. Rev. Microbiol..

[14] Cryan J.F., Dinan T.G. (2015). More than a gut feeling: The microbiota regulates neurodevelopment and behavior. Neuropsychopharmacology.

[15] Rhee S.H., Pothoulakis C., Mayer E.A. (2009). Principles and clinical implications of the brain-gut-enteric microbiota axis. Nat. Rev. Gastroenterol. Hepatol..

[16] Bailey M.T., Lubach G.R., Coe C.L. (2004). Prenatal stress alters bacterial colonization of the gut in infant monkeys. J. Pediatr. Gastroenterol. Nutr..

[17] Bailey M.T., Coe C.L. (1999). Maternal separation disrupts the integrity of the intestinal microflora in infant rhesus monkeys. Dev. Psychobiol..

[18] O’Mahony S.M., Marchesi J.R., Scully P., Codling C., Ceolho A.M., Quigley E.M., Cryan J.F., Dinan T.G. (2009). Early life stress alters behavior, immunity, and microbiota in rats: Implications for irritable bowel syndrome and psychiatric illnesses. Biol. Psychiatry.

[19] Kiliaan A.J., Saunders P.R., Bijlsma P.B., Berin M.C., Taminiau J.A., Groot J.A., Perdue M.H. (1998). Stress stimulates transepithelial macromolecular uptake in rat jejunum. Am. J. Physiol..

[20] Demaude J., Salvador-Cartier C., Fioramonti J., Ferrier L., Bueno L. (2006). Phenotypic changes in colonocytes following acute stress or activation of mast cells in mice: Implications for delayed epithelial barrier dysfunction. Gut.

[21] Bailey M.T., Engler H., Sheridan J.F. (2006). Stress induces the translocation of cutaneous and gastrointestinal microflora to secondary lymphoid organs of C57BL/6 mice. J. Neuroimmunol..

[22] Freestone P.P., Williams P.H., Haigh R.D., Maggs A.F., Neal C.P., Lyte M. (2002). Growth stimulation of intestinal commensal Escherichia coli by catecholamines: A possible contributory factor in trauma-induced sepsis. Shock.

[23] Freestone P.P., Haigh R.D., Williams P.H., Lyte M. (2003). Involvement of enterobactin in norepinephrine-mediated iron supply from transferrin to enterohaemorrhagic Escherichia coli. FEMS Microbiol. Lett..

[24] Lyte M., Ernst S. (1992). Catecholamine induced growth of gram negative bacteria. Life Sci..

[25] Lyte M., Freestone P.P., Neal C.P., Olson B.A., Haigh R.D., Bayston R., Williams P.H. (2003). Stimulation of Staphylococcus epidermidis growth and biofilm formation by catecholamine inotropes. Lancet.

[26] Hegde M., Wood T.K., Jayaraman A. (2009). The neuroendocrine hormone norepinephrine increases *Pseudomonas aeruginosa* PA14 virulence through the las quorum-sensing pathway. Appl. Microbiol. Biotechnol..

[27] Sperandio V., Torres A.G., Jarvis B., Nataro J.P., Kaper J.B. (2003). Bacteria-host communication: The language of hormones. Proc. Natl. Acad. Sci. USA.

[28] Kasubuchi M., Hasegawa S., Hiramatsu T., Ichimura A., Kimura I. (2015). Dietary gut microbial metabolites, short-chain fatty acids, and host metabolic regulation. Nutrients.

[29] Queipo-Ortuno M.I., Seoane L.M., Murri M., Pardo M., Gomez-Zumaquero J.M., Cardona F., Casanueva F., Tinahones F.J. (2013). Gut microbiota composition in male rat models under different nutritional status and physical activity and its association with serum leptin and ghrelin levels. PLoS ONE.

[30] Parnell J.A., Reimer R.A. (2009). Weight loss during oligofructose supplementation is associated with decreased ghrelin and increased peptide YY in overweight and obese adults. Am. J. Clin. Nutr..

[31] Ravussin Y., Koren O., Spor A., LeDuc C., Gutman R., Stombaugh J., Knight R., Ley R.E., Leibel R.L. (2012). Responses of gut microbiota to diet composition and weight loss in lean and obese mice. Obesity.

[32] Naruszewicz M., Johansson M.L., Zapolska-Downar D., Bukowska H. (2002). Effect of Lactobacillus plantarum 299v on cardiovascular disease risk factors in smokers. Am. J. Clin. Nutr..

[33] Lam V., Su J., Koprowski S., Hsu A., Tweddell J.S., Rafiee P., Gross G.J., Salzman N.H., Baker J.E. (2012). Intestinal microbiota determine severity of myocardial infarction in rats. FASEB J..

[34] Karlsson F.H., Tremaroli V., Nookaew I., Bergstrom G., Behre C.J., Fagerberg B., Nielsen J., Backhed F. (2013). Gut metagenome in European women with normal, impaired and diabetic glucose control. Nature.

[35] LeRoith D., Pickens W., Vinik A.I., Shiloach J. (1985). Bacillus subtilis contains multiple forms of somatostatin-like material. Biochem. Biophys. Res. Commun..

[36] Yadav H., Lee J.H., Lloyd J., Walter P., Rane S.G. (2013). Beneficial metabolic effects of a probiotic via butyrate-induced GLP-1 hormone secretion. J. Biol. Chem..

[37] Wichmann A., Allahyar A., Greiner T.U., Plovier H., Lunden G.O., Larsson T., Drucker D.J., Delzenne N.M., Cani P.D., Backhed F. (2013). Microbial modulation of energy availability in the colon regulates intestinal transit. Cell Host Microbe.

[38] Fetissov S.O., Hamze Sinno M., Coeffier M., Bole-Feysot C., Ducrotte P., Hokfelt T., Dechelotte P. (2008). Autoantibodies against appetite-regulating peptide hormones and neuropeptides: Putative modulation by gut microflora. Nutrition.

[39] Grenham S., Clarke G., Cryan J.F., Dinan T.G. (2011). Brain-gut-microbe communication in health and disease. Front. Physiol..

[40] O’Hara A.M., Shanahan F. (2006). The gut flora as a forgotten organ. EMBO Rep..

[41] Neufeld K.M., Kang N., Bienenstock J., Foster J.A. (2011). Reduced anxiety-like behavior and central neurochemical change in germ-free mice. Neurogastroenterol. Motil..

[42] Bercik P., Denou E., Collins J., Jackson W., Lu J., Jury J., Deng Y., Blennerhassett P., Macri J., McCoy K.D. (2011). The intestinal microbiota affect central levels of brain-derived neurotropic factor and behavior in mice. Gastroenterology.

[43] Sudo N., Chida Y., Aiba Y., Sonoda J., Oyama N., Yu X.N., Kubo C., Koga Y. (2004). Postnatal microbial colonization programs the hypothalamic-pituitary-adrenal system for stress response in mice. J. Physiol..

[44] Messaoudi M., Lalonde R., Violle N., Javelot H., Desor D., Nejdi A., Bisson J.F., Rougeot C., Pichelin M., Cazaubiel M. (2011). Assessment of psychotropic-like properties of a probiotic formulation (*Lactobacillus helveticus* R0052 and *Bifidobacterium longum* R0175) in rats and human subjects. Br. J. Nutr..

[45] Bravo J.A., Forsythe P., Chew M.V., Escaravage E., Savignac H.M., Dinan T.G., Bienenstock J., Cryan J.F. (2011). Ingestion of Lactobacillus strain regulates emotional behavior and central GABA receptor expression in a mouse via the vagus nerve. Proc. Natl. Acad. Sci. USA.

[46] Markle J.G., Frank D.N., Mortin-Toth S., Robertson C.E., Feazel L.M., Rolle-Kampczyk U., von Bergen M., McCoy K.D., Macpherson A.J., Danska J.S. (2013). Sex differences in the gut microbiome drive hormone-dependent regulation of autoimmunity. Science.

[47] Asano Y., Hiramoto T., Nishino R., Aiba Y., Kimura T., Yoshihara K., Koga Y., Sudo N. (2012). Critical role of gut microbiota in the production of biologically active, free catecholamines in the gut lumen of mice. Am. J. Physiol. Gastrointest. Liver Physiol..

[48] Lyte M. (2013). Microbial endocrinology in the microbiome-gut-brain axis: How bacterial production and utilization of neurochemicals influence behavior. PLoS Pathog..

[49] Lyte M. (2014). Microbial endocrinology and the microbiota-gut-brain axis. Adv. Exp. Med. Biol..

[50] Reigstad C.S., Salmonson C.E., Rainey J.F., Szurszewski J.H., Linden D.R., Sonnenburg J.L., Farrugia G., Kashyap P.C. (2015). Gut microbes promote colonic serotonin production through an effect of short-chain fatty acids on enterochromaffin cells. FASEB J..

[51] Clarke G., Grenham S., Scully P., Fitzgerald P., Moloney R.D., Shanahan F., Dinan T.G., Cryan J.F. (2013). The microbiome-gut-brain axis during early life regulates the hippocampal serotonergic system in a sex-dependent manner. Mol. Psychiatry.

[52] Wikoff W.R., Anfora A.T., Liu J., Schultz P.G., Lesley S.A., Peters E.C., Siuzdak G. (2009). Metabolomics analysis reveals large effects of gut microflora on mammalian blood metabolites. Proc. Natl. Acad. Sci. USA.

[53] Desbonnet L., Garrett L., Clarke G., Bienenstock J., Dinan T.G. (2008). The probiotic Bifidobacteria infantis: An assessment of potential antidepressant properties in the rat. J. Psychiatr. Res..

[54] Donia M.S., Fischbach M.A. (2015). Small molecules from the human microbiota. Science.

[55] Nuclear Receptors Nomenclature Committee (1999). A unified nomenclature system for the nuclear receptor superfamily. Cell.

[56] Desvergne B., Wahli W. (1999). Peroxisome proliferator-activated receptors: Nuclear control of metabolism. Endocr. Rev..

[57] Green S., Wahli W. (1994). Peroxisome proliferator-activated receptors: Finding the orphan a home. Mol. Cell. Endocrinol..

[58] Braissant O., Foufelle F., Scotto C., Dauca M., Wahli W. (1996). Differential expression of peroxisome proliferator-activated receptors (PPARs): Tissue distribution of PPAR-α, -β, and -γ in the adult rat. Endocrinology.

[59] Duszka K., Ellero-Simatos S., Ow G.G., Defernez M., Paramalingam E., Tett A., Ying S., König J., Narbad A., Kuznetsov V.A. (2018). Complementary intestinal mucosa and microbiota responses to caloric restriction. Sci. Rep..

[60] Issemann I., Green S. (1990). Activation of a member of the steroid hormone receptor superfamily by peroxisome proliferators. Nature.

[61] Hashimoto T., Cook W.S., Qi C., Yeldandi A.V., Reddy J.K., Rao M.S. (2000). Defect in peroxisome proliferator-activated receptor α-inducible fatty acid oxidation determines the severity of hepatic steatosis in response to fasting. J. Biol. Chem..

[62] Kersten S., Seydoux J., Peters J.M., Gonzalez F.J., Desvergne B., Wahli W. (1999). Peroxisome proliferator-activated receptor α mediates the adaptive response to fasting. J. Clin. Investig..

[63] Leone T.C., Weinheimer C.J., Kelly D.P. (1999). A critical role for the peroxisome proliferator-activated receptor α (PPARα) in the cellular fasting response: The PPARα-null mouse as a model of fatty acid oxidation disorders. Proc. Natl. Acad. Sci. USA.

[64] Rodriguez J.C., Gil-Gomez G., Hegardt F.G., Haro D. (1994). Peroxisome proliferator-activated receptor mediates induction of the mitochondrial 3-hydroxy-3-methylglutaryl-CoA synthase gene by fatty acids. J. Biol. Chem..

[65] Badman M.K., Pissios P., Kennedy A.R., Koukos G., Flier J.S., Maratos-Flier E. (2007). Hepatic fibroblast growth factor 21 is regulated by PPARα and is a key mediator of hepatic lipid metabolism in ketotic states. Cell Metab..

[66] Crawford P.A., Crowley J.R., Sambandam N., Muegge B.D., Costello E.K., Hamady M., Knight R., Gordon J.I. (2009). Regulation of myocardial ketone body metabolism by the gut microbiota during nutrient deprivation. Proc. Natl. Acad. Sci. USA.

[67] Schmutz I., Ripperger J.A., Baeriswyl-Aebischer S., Albrecht U. (2010). The mammalian clock component PERIOD2 coordinates circadian output by interaction with nuclear receptors. Genes Dev..

[68] Canaple L., Rambaud J., Dkhissi-Benyahya O., Rayet B., Tan N.S., Michalik L., Delaunay F., Wahli W., Laudet V. (2006). Reciprocal regulation of brain and muscle Arnt-like protein 1 and peroxisome proliferator-activated receptor α defines a novel positive feedback loop in the rodent liver circadian clock. Mol. Endocrinol..

[69] Montagner A., Rando G., Degueurce G., Leuenberger N., Michalik L., Wahli W. (2011). New insights into the role of PPARs. Prostaglandins Leukot. Essent. Fatty Acids.

[70] Corton J.C., Apte U., Anderson S.P., Limaye P., Yoon L., Latendresse J., Dunn C., Everitt J.I., Voss K.A., Swanson C. (2004). Mimetics of caloric restriction include agonists of lipid-activated nuclear receptors. J. Biol. Chem..

[71] Fontaine C., Dubois G., Duguay Y., Helledie T., Vu-Dac N., Gervois P., Soncin F., Mandrup S., Fruchart J.C., Fruchart-Najib J. (2003). The orphan nuclear receptor Rev-Erbα is a peroxisome proliferator-activated receptor (PPAR) γ target gene and promotes PPARγ-induced adipocyte differentiation. J. Biol. Chem..

[72] Oishi K., Shirai H., Ishida N. (2005). CLOCK is involved in the circadian transactivation of peroxisome-proliferator-activated receptor α (PPARα) in mice. Biochem. J..

[73] Gibbons G.F., Patel D., Wiggins D., Knight B.L. (2002). The functional efficiency of lipogenic and cholesterogenic gene expression in normal mice and in mice lacking the peroxisomal proliferator-activated receptor-α (PPAR-α). Adv. Enzym. Regul..

[74] Patel D.D., Knight B.L., Wiggins D., Humphreys S.M., Gibbons G.F. (2001). Disturbances in the normal regulation of SREBP-sensitive genes in PPAR α-deficient mice. J. Lipid Res..

[75] Mukherji A., Kobiita A., Ye T., Chambon P. (2013). Homeostasis in intestinal epithelium is orchestrated by the circadian clock and microbiota cues transduced by TLRs. Cell.

[76] Manoharan I., Suryawanshi A., Hong Y., Ranganathan P., Shanmugam A., Ahmad S., Swafford D., Manicassamy B., Ramesh G., Koni P.A. (2016). Homeostatic PPARα Signaling Limits Inflammatory Responses to Commensal Microbiota in the Intestine. J. Immunol..

[77] Mazzon E., Cuzzocrea S. (2007). Absence of functional peroxisome proliferator-activated receptor-α enhanced ileum permeability during experimental colitis. Shock.

[78] Riccardi L., Mazzon E., Bruscoli S., Esposito E., Crisafulli C., Di Paola R., Caminiti R., Riccardi C., Cuzzocrea S. (2009). Peroxisome proliferator-activated receptor-α modulates the anti-inflammatory effect of glucocorticoids in a model of inflammatory bowel disease in mice. Shock.

[79] Esposito E., Mazzon E., Paterniti I., Dal Toso R., Pressi G., Caminiti R., Cuzzocrea S. (2010). PPAR-α Contributes to the Anti-Inflammatory Activity of Verbascoside in a Model of Inflammatory Bowel Disease in Mice. PPAR Res..

[80] Cuzzocrea S., Di Paola R., Mazzon E., Genovese T., Muia C., Centorrino T., Caputi A.P. (2004). Role of endogenous and exogenous ligands for the peroxisome proliferators activated receptors α (PPAR-α) in the development of inflammatory bowel disease in mice. Lab. Investig..

[81] Fu J., Gaetani S., Oveisi F., Lo Verme J., Serrano A., Rodriguez De Fonseca F., Rosengarth A., Luecke H., Di Giacomo B., Tarzia G. (2003). Oleylethanolamide regulates feeding and body weight through activation of the nuclear receptor PPAR-α. Nature.

[82] Fu J., Dipatrizio N.V., Guijarro A., Schwartz G.J., Li X., Gaetani S., Astarita G., Piomelli D. (2011). Sympathetic activity controls fat-induced oleoylethanolamide signaling in small intestine. J. Neurosci..

[83] Magotti P., Bauer I., Igarashi M., Babagoli M., Marotta R., Piomelli D., Garau G. (2015). Structure of human *N*-acylphosphatidylethanolamine-hydrolyzing phospholipase D: Regulation of fatty acid ethanolamide biosynthesis by bile acids. Structure.

[84] Pineda Torra I., Claudel T., Duval C., Kosykh V., Fruchart J.C., Staels B. (2003). Bile acids induce the expression of the human peroxisome proliferator-activated receptor α gene via activation of the farnesoid X receptor. Mol. Endocrinol..

[85] Gaetani S., Oveisi F., Piomelli D. (2003). Modulation of meal pattern in the rat by the anorexic lipid mediator oleoylethanolamide. Neuropsychopharmacology.

[86] Fu J., Oveisi F., Gaetani S., Lin E., Piomelli D. (2005). Oleoylethanolamide, an endogenous PPAR-α agonist, lowers body weight and hyperlipidemia in obese rats. Neuropharmacology.

[87] Caillon A., Duszka K., Wahli W., Rohner-Jeanrenaud F., Altirriba J. (2018). The OEA effect on food intake is independent from the presence of PPARα in the intestine and the nodose ganglion, while the impact of OEA on energy expenditure requires the presence of PPARα in mice. Metabolism.

[88] Lo Verme J., Gaetani S., Fu J., Oveisi F., Burton K., Piomelli D. (2005). Regulation of food intake by oleoylethanolamide. Cell. Mol. Life Sci..

[89] Piomelli D. (2013). A fatty gut feeling. Trends Endocrinol. Metab..

[90] Proulx K., Cota D., Castaneda T.R., Tschop M.H., D’Alessio D.A., Tso P., Woods S.C., Seeley R.J. (2005). Mechanisms of oleoylethanolamide-induced changes in feeding behavior and motor activity. Am. J. Physiol. Regul. Integr. Comp. Physiol..

[91] Rodriguez de Fonseca F., Navarro M., Gomez R., Escuredo L., Nava F., Fu J., Murillo-Rodriguez E., Giuffrida A., LoVerme J., Gaetani S. (2001). An anorexic lipid mediator regulated by feeding. Nature.

[92] Gaetani S., Fu J., Cassano T., Dipasquale P., Romano A., Righetti L., Cianci S., Laconca L., Giannini E., Scaccianoce S. (2010). The fat-induced satiety factor oleoylethanolamide suppresses feeding through central release of oxytocin. J. Neurosci..

[93] Tan N.S., Michalik L., Di-Poi N., Ng C.Y., Mermod N., Roberts A.B., Desvergne B., Wahli W. (2004). Essential role of Smad3 in the inhibition of inflammation-induced PPARβ/δ expression. EMBO J..

[94] Varnat F., Heggeler B.B., Grisel P., Boucard N., Corthesy-Theulaz I., Wahli W., Desvergne B. (2006). PPARβ/δ regulates paneth cell differentiation via controlling the hedgehog signaling pathway. Gastroenterology.

[95] Peters J.M., Hollingshead H.E., Gonzalez F.J. (2008). Role of peroxisome-proliferator-activated receptor β/δ (PPARβ/δ) in gastrointestinal tract function and disease. Clin. Sci..

[96] Burdick A.D., Kim D.J., Peraza M.A., Gonzalez F.J., Peters J.M. (2006). The role of peroxisome proliferator-activated receptor-β/δ in epithelial cell growth and differentiation. Cell Signal..

[97] Hollingshead H.E., Morimura K., Adachi M., Kennett M.J., Billin A.N., Willson T.M., Gonzalez F.J., Peters J.M. (2007). PPARβ/δ protects against experimental colitis through a ligand-independent mechanism. Dig. Dis. Sci..

[98] Wahli W. (2008). A gut feeling of the PXR, PPAR and NF-κB connection. J. Intern. Med..

[99] Doktorova M., Zwarts I., Zutphen T.V., Dijk T.H., Bloks V.W., Harkema L., Bruin A., Downes M., Evans R.M., Verkade H.J. (2017). Intestinal PPARδ protects against diet-induced obesity, insulin resistance and dyslipidemia. Sci. Rep..

[100] Daoudi M., Hennuyer N., Borland M.G., Touche V., Duhem C., Gross B., Caiazzo R., Kerr-Conte J., Pattou F., Peters J.M. (2011). PPARβ/δ activation induces enteroendocrine L cell GLP-1 production. Gastroenterology.

[101] Anghel S.I., Wahli W. (2007). Fat poetry: A kingdom for PPAR γ. Cell Res..

[102] Auwerx J. (2002). Nuclear receptors. I. PPAR γ in the gastrointestinal tract: Gain or pain?. Am. J. Physiol. Gastrointest. Liver Physiol..

[103] Dubuquoy L., Rousseaux C., Thuru X., Peyrin-Biroulet L., Romano O., Chavatte P., Chamaillard M., Desreumaux P. (2006). PPARγ as a new therapeutic target in inflammatory bowel diseases. Gut.

[104] Leonardini A., Laviola L., Perrini S., Natalicchio A., Giorgino F. (2009). Cross-Talk between PPARγ and Insulin Signaling and Modulation of Insulin Sensitivity. PPAR Res..

[105] Tontonoz P., Spiegelman B.M. (2008). Fat and beyond: The diverse biology of PPARγ. Annu. Rev. Biochem..

[106] Escher P., Braissant O., Basu-Modak S., Michalik L., Wahli W., Desvergne B. (2001). Rat PPARs: Quantitative analysis in adult rat tissues and regulation in fasting and refeeding. Endocrinology.

[107] Harmon G.S., Dumlao D.S., Ng D.T., Barrett K.E., Dennis E.A., Dong H., Glass C.K. (2010). Pharmacological correction of a defect in PPAR-γ signaling ameliorates disease severity in Cftr-deficient mice. Nat. Med..

[108] Mansen A., Guardiola-Diaz H., Rafter J., Branting C., Gustafsson J.A. (1996). Expression of the peroxisome proliferator-activated receptor (PPAR) in the mouse colonic mucosa. Biochem. Biophys. Res. Commun..

[109] Marion-Letellier R., Dechelotte P., Iacucci M., Ghosh S. (2009). Dietary modulation of peroxisome proliferator-activated receptor γ. Gut.

[110] Schwab M., Reynders V., Loitsch S., Steinhilber D., Stein J., Schroder O. (2007). Involvement of different nuclear hormone receptors in butyrate-mediated inhibition of inducible NF κ B signalling. Mol. Immunol..

[111] Wachtershauser A., Loitsch S.M., Stein J. (2000). PPAR-γ is selectively upregulated in Caco-2 cells by butyrate. Biochem. Biophys. Res. Commun..

[112] Nepelska M., de Wouters T., Jacouton E., Beguet-Crespel F., Lapaque N., Dore J., Arulampalam V., Blottiere H.M. (2017). Commensal gut bacteria modulate phosphorylation-dependent PPARγ transcriptional activity in human intestinal epithelial cells. Sci. Rep..

[113] Voltan S., Martines D., Elli M., Brun P., Longo S., Porzionato A., Macchi V., D’Inca R., Scarpa M., Palu G. (2008). Lactobacillus crispatus M247-derived H_2_O_2_ acts as a signal transducing molecule activating peroxisome proliferator activated receptor-γ in the intestinal mucosa. Gastroenterology.

[114] Are A., Aronsson L., Wang S., Greicius G., Lee Y.K., Gustafsson J.A., Pettersson S., Arulampalam V. (2008). Enterococcus faecalis from newborn babies regulate endogenous PPARγ activity and IL-10 levels in colonic epithelial cells. Proc. Natl. Acad. Sci. USA.

[115] Couvigny B., de Wouters T., Kaci G., Jacouton E., Delorme C., Dore J., Renault P., Blottiere H.M., Guedon E., Lapaque N. (2015). Commensal Streptococcus salivarius Modulates PPARγ Transcriptional Activity in Human Intestinal Epithelial Cells. PLoS ONE.

[116] Murakami M., Tognini P., Liu Y., Eckel-Mahan K.L., Baldi P., Sassone-Corsi P. (2016). Gut microbiota directs PPARγ-driven reprogramming of the liver circadian clock by nutritional challenge. EMBO Rep..

[117] Cerbone A., Toaldo C., Laurora S., Briatore F., Pizzimenti S., Dianzani M.U., Ferretti C., Barrera G. (2007). 4-Hydroxynonenal and PPARγ ligands affect proliferation, differentiation, and apoptosis in colon cancer cells. Free Radic. Biol. Med..

[118] Martinasso G., Oraldi M., Trombetta A., Maggiora M., Bertetto O., Canuto R.A., Muzio G. (2007). Involvement of PPARs in Cell Proliferation and Apoptosis in Human Colon Cancer Specimens and in Normal and Cancer Cell Lines. PPAR Res..

[119] Theocharis S., Margeli A., Vielh P., Kouraklis G. (2004). Peroxisome proliferator-activated receptor-γ ligands as cell-cycle modulators. Cancer Treat. Rev..

[120] Xu W.P., Zhang X., Xie W.F. (2014). Differentiation therapy for solid tumors. J. Dig. Dis..

[121] Chen G.G., Lee J.F., Wang S.H., Chan U.P., Ip P.C., Lau W.Y. (2002). Apoptosis induced by activation of peroxisome-proliferator activated receptor-γ is associated with Bcl-2 and NF-κB in human colon cancer. Life Sci..

[122] Chen G.G., Xu H., Lee J.F., Subramaniam M., Leung K.L., Wang S.H., Chan U.P., Spelsberg T.C. (2003). 15-hydroxy-eicosatetraenoic acid arrests growth of colorectal cancer cells via a peroxisome proliferator-activated receptor γ-dependent pathway. Int. J. Cancer.

[123] Lee C.J., Han J.S., Seo C.Y., Park T.H., Kwon H.C., Jeong J.S., Kim I.H., Yun J., Bae Y.S., Kwak J.Y. (2006). Pioglitazone, a synthetic ligand for PPARγ, induces apoptosis in RB-deficient human colorectal cancer cells. Apoptosis.

[124] Sharma C., Pradeep A., Wong L., Rana A., Rana B. (2004). Peroxisome proliferator-activated receptor γ activation can regulate β-catenin levels via a proteasome-mediated and adenomatous polyposis coli-independent pathway. J. Biol. Chem..

[125] Lewis J.D., Lichtenstein G.R., Deren J.J., Sands B.E., Hanauer S.B., Katz J.A., Lashner B., Present D.H., Chuai S., Ellenberg J.H. (2008). Rosiglitazone for active ulcerative colitis: A randomized placebo-controlled trial. Gastroenterology.

[126] Lewis J.D., Lichtenstein G.R., Stein R.B., Deren J.J., Judge T.A., Fogt F., Furth E.E., Demissie E.J., Hurd L.B., Su C.G. (2001). An open-label trial of the PPAR-γ ligand rosiglitazone for active ulcerative colitis. Am. J. Gastroenterol..

[127] Liang H.L., Ouyang Q. (2008). A clinical trial of combined use of rosiglitazone and 5-aminosalicylate for ulcerative colitis. World J. Gastroenterol..

[128] Bassaganya-Riera J., Hontecillas R. (2006). CLA and n-3 PUFA differentially modulate clinical activity and colonic PPAR-responsive gene expression in a pig model of experimental IBD. Clin. Nutr..

[129] Hontecillas R., Wannemeulher M.J., Zimmerman D.R., Hutto D.L., Wilson J.H., Ahn D.U., Bassaganya-Riera J. (2002). Nutritional regulation of porcine bacterial-induced colitis by conjugated linoleic acid. J. Nutr..

[130] Sanchez-Hidalgo M., Martin A.R., Villegas I., de la Lastra C.A. (2007). Rosiglitazone, a PPARγ ligand, modulates signal transduction pathways during the development of acute TNBS-induced colitis in rats. Eur. J. Pharmacol..

[131] Sato N., Kozar R.A., Zou L., Weatherall J.M., Attuwaybi B., Moore-Olufemi S.D., Weisbrodt N.W., Moore F.A. (2005). Peroxisome proliferator-activated receptor γ mediates protection against cyclooxygenase-2-induced gut dysfunction in a rodent model of mesenteric ischemia/reperfusion. Shock.

[132] Saubermann L.J., Nakajima A., Wada K., Zhao S., Terauchi Y., Kadowaki T., Aburatani H., Matsuhashi N., Nagai R., Blumberg R.S. (2002). Peroxisome proliferator-activated receptor γ agonist ligands stimulate a Th2 cytokine response and prevent acute colitis. Inflamm. Bowel Dis..

[133] Su C.G., Wen X., Bailey S.T., Jiang W., Rangwala S.M., Keilbaugh S.A., Flanigan A., Murthy S., Lazar M.A., Wu G.D. (1999). A novel therapy for colitis utilizing PPAR-γ ligands to inhibit the epithelial inflammatory response. J. Clin. Investig..

[134] Kundu P., Ling T.W., Korecka A., Li Y., D’Arienzo R., Bunte R.M., Berger T., Arulampalam V., Chambon P., Mak T.W. (2014). Absence of intestinal PPARγ aggravates acute infectious colitis in mice through a lipocalin-2-dependent pathway. PLoS Pathog..

[135] Michalik L., Wahli W. (2008). PPARs Mediate Lipid Signaling in Inflammation and Cancer. PPAR Res..

[136] Shah Y.M., Morimura K., Gonzalez F.J. (2007). Expression of peroxisome proliferator-activated receptor-γ in macrophage suppresses experimentally induced colitis. Am. J. Physiol. Gastrointest. Liver Physiol..

[137] Leung E., Hong J., Fraser A., Merriman T., Krissansen G. (2006). PPAR-γ and Crohn’s disease in New Zealand. Gastroenterology.

[138] Wada K., Nakajima A., Blumberg R.S. (2001). PPARγ and inflammatory bowel disease: A new therapeutic target for ulcerative colitis and Crohn’s disease. Trends Mol. Med..

[139] Yasui Y., Hosokawa M., Sahara T., Suzuki R., Ohgiya S., Kohno H., Tanaka T., Miyashita K. (2005). Bitter gourd seed fatty acid rich in 9c,11t,13t-conjugated linolenic acid induces apoptosis and up-regulates the GADD45, p53 and PPARγ in human colon cancer Caco-2 cells. Prostaglandins Leukot. Essent. Fatty Acids.

[140] Akinyeke T.O., Stewart L.V. (2011). Troglitazone suppresses c-Myc levels in human prostate cancer cells via a PPARγ-independent mechanism. Cancer Biol. Ther..

[141] Yang W.L., Frucht H. (2001). Activation of the PPAR pathway induces apoptosis and COX-2 inhibition in HT-29 human colon cancer cells. Carcinogenesis.

[142] Subbaramaiah K., Lin D.T., Hart J.C., Dannenberg A.J. (2001). Peroxisome proliferator-activated receptor γ ligands suppress the transcriptional activation of cyclooxygenase-2. Evidence for involvement of activator protein-1 and CREB-binding protein/p300. J. Biol. Chem..

[143] Vandoros G.P., Konstantinopoulos P.A., Sotiropoulou-Bonikou G., Kominea A., Papachristou G.I., Karamouzis M.V., Gkermpesi M., Varakis I., Papavassiliou A.G. (2006). PPAR-γ is expressed and NF-kB pathway is activated and correlates positively with COX-2 expression in stromal myofibroblasts surrounding colon adenocarcinomas. J. Cancer Res. Clin. Oncol..

[144] Girnun G.D., Smith W.M., Drori S., Sarraf P., Mueller E., Eng C., Nambiar P., Rosenberg D.W., Bronson R.T., Edelmann W. (2002). APC-dependent suppression of colon carcinogenesis by PPARγ. Proc. Natl. Acad. Sci. USA.

[145] Fujisawa T., Nakajima A., Fujisawa N., Takahashi H., Ikeda I., Tomimoto A., Yonemitsu K., Nakajima N., Kudo C., Wada K. (2008). Peroxisome proliferator-activated receptor γ (PPARγ) suppresses colonic epithelial cell turnover and colon carcinogenesis through inhibition of the β-catenin/T cell factor (TCF) pathway. J. Pharmacol. Sci..

[146] Peyrin-Biroulet L., Beisner J., Wang G., Nuding S., Oommen S.T., Kelly D., Parmentier-Decrucq E., Dessein R., Merour E., Chavatte P. (2010). Peroxisome proliferator-activated receptor γ activation is required for maintenance of innate antimicrobial immunity in the colon. Proc. Natl. Acad. Sci. USA.

[147] Duszka K., Oresic M., Le May C., Konig J., Wahli W. (2017). PPARγ Modulates Long Chain Fatty Acid Processing in the Intestinal Epithelium. Int. J. Mol. Sci..

[148] Duszka K., Picard A., Ellero-Simatos S., Chen J., Defernez M., Paramalingam E., Pigram A., Vanoaica L., Canlet C., Parini P. (2016). Intestinal PPARγ signalling is required for sympathetic nervous system activation in response to caloric restriction. Sci. Rep..

[149] Parseus A., Sommer N., Sommer F., Caesar R., Molinaro A., Stahlman M., Greiner T.U., Perkins R., Backhed F. (2017). Microbiota-induced obesity requires farnesoid X receptor. Gut.

[150] Midtvedt T. (1974). Microbial bile acid transformation. Am. J. Clin. Nutr..

[151] Begley M., Gahan C.G., Hill C. (2005). The interaction between bacteria and bile. FEMS Microbiol. Rev..

[152] Buffie C.G., Bucci V., Stein R.R., McKenney P.T., Ling L., Gobourne A., No D., Liu H., Kinnebrew M., Viale A. (2015). Precision microbiome reconstitution restores bile acid mediated resistance to *Clostridium difficile*. Nature.

[153] Inagaki T., Moschetta A., Lee Y.K., Peng L., Zhao G., Downes M., Yu R.T., Shelton J.M., Richardson J.A., Repa J.J. (2006). Regulation of antibacterial defense in the small intestine by the nuclear bile acid receptor. Proc. Natl. Acad. Sci. USA.

[154] Kim I., Ahn S.H., Inagaki T., Choi M., Ito S., Guo G.L., Kliewer S.A., Gonzalez F.J. (2007). Differential regulation of bile acid homeostasis by the farnesoid X receptor in liver and intestine. J. Lipid Res..

[155] Makishima M., Okamoto A.Y., Repa J.J., Tu H., Learned R.M., Luk A., Hull M.V., Lustig K.D., Mangelsdorf D.J., Shan B. (1999). Identification of a nuclear receptor for bile acids. Science.

[156] Nguyen A., Bouscarel B. (2008). Bile acids and signal transduction: Role in glucose homeostasis. Cell Signal..

[157] Lee J.M., Wagner M., Xiao R., Kim K.H., Feng D., Lazar M.A., Moore D.D. (2014). Nutrient-sensing nuclear receptors coordinate autophagy. Nature.

[158] Seok S., Fu T., Choi S.E., Li Y., Zhu R., Kumar S., Sun X., Yoon G., Kang Y., Zhong W. (2014). Transcriptional regulation of autophagy by an FXR-CREB axis. Nature.

[159] Li F., Jiang C., Krausz K.W., Li Y., Albert I., Hao H., Fabre K.M., Mitchell J.B., Patterson A.D., Gonzalez F.J. (2013). Microbiome remodelling leads to inhibition of intestinal farnesoid X receptor signalling and decreased obesity. Nat. Commun..

[160] Jiang C., Xie C., Li F., Zhang L., Nichols R.G., Krausz K.W., Cai J., Qi Y., Fang Z.Z., Takahashi S. (2015). Intestinal farnesoid X receptor signaling promotes nonalcoholic fatty liver disease. J. Clin. Investog..

[161] Schmitt J., Kong B., Stieger B., Tschopp O., Schultze S.M., Rau M., Weber A., Mullhaupt B., Guo G.L., Geier A. (2015). Protective effects of farnesoid X receptor (FXR) on hepatic lipid accumulation are mediated by hepatic FXR and independent of intestinal FGF15 signal. Liver Int..

[162] Rao A., Kosters A., Mells J.E., Zhang W., Setchell K.D., Amanso A.M., Wynn G.M., Xu T., Keller B.T., Yin H. (2016). Inhibition of ileal bile acid uptake protects against nonalcoholic fatty liver disease in high-fat diet-fed mice. Sci. Transl. Med..

[163] Raju U., Levitz M., Javitt N.B. (1990). Bile acids in human breast cyst fluid: The identification of lithocholic acid. J. Clin. Endocrinol. Metab..

[164] Javitt N.B., Budai K., Miller D.G., Cahan A.C., Raju U., Levitz M. (1994). Breast-gut connection: Origin of chenodeoxycholic acid in breast cyst fluid. Lancet.

[165] Swales K.E., Korbonits M., Carpenter R., Walsh D.T., Warner T.D., Bishop-Bailey D. (2006). The farnesoid X receptor is expressed in breast cancer and regulates apoptosis and aromatase expression. Cancer Res..

[166] Inagaki T., Choi M., Moschetta A., Peng L., Cummins C.L., McDonald J.G., Luo G., Jones S.A., Goodwin B., Richardson J.A. (2005). Fibroblast growth factor 15 functions as an enterohepatic signal to regulate bile acid homeostasis. Cell Metab..

[167] Lundasen T., Galman C., Angelin B., Rudling M. (2006). Circulating intestinal fibroblast growth factor 19 has a pronounced diurnal variation and modulates hepatic bile acid synthesis in man. J. Intern. Med..

[168] Lin B.C., Wang M., Blackmore C., Desnoyers L.R. (2007). Liver-specific activities of FGF19 require Klotho β. J. Biol. Chem..

[169] Choi M., Moschetta A., Bookout A.L., Peng L., Umetani M., Holmstrom S.R., Suino-Powell K., Xu H.E., Richardson J.A., Gerard R.D. (2006). Identification of a hormonal basis for gallbladder filling. Nat. Med..

[170] Kurosu H., Choi M., Ogawa Y., Dickson A.S., Goetz R., Eliseenkova A.V., Mohammadi M., Rosenblatt K.P., Kliewer S.A., Kuro-o M. (2007). Tissue-specific expression of βKlotho and fibroblast growth factor (FGF) receptor isoforms determines metabolic activity of FGF19 and FGF21. J. Biol. Chem..

[171] Shin D.J., Osborne T.F. (2009). FGF15/FGFR4 integrates growth factor signaling with hepatic bile acid metabolism and insulin action. J. Biol. Chem..

[172] Kir S., Beddow S.A., Samuel V.T., Miller P., Previs S.F., Suino-Powell K., Xu H.E., Shulman G.I., Kliewer S.A., Mangelsdorf D.J. (2011). FGF19 as a postprandial, insulin-independent activator of hepatic protein and glycogen synthesis. Science.

[173] Huang X., Yang C., Luo Y., Jin C., Wang F., McKeehan W.L. (2007). FGFR4 prevents hyperlipidemia and insulin resistance but underlies high-fat diet induced fatty liver. Diabetes.

[174] Potthoff M.J., Boney-Montoya J., Choi M., He T., Sunny N.E., Satapati S., Suino-Powell K., Xu H.E., Gerard R.D., Finck B.N. (2011). FGF15/19 regulates hepatic glucose metabolism by inhibiting the CREB-PGC-1α pathway. Cell Metab..

[175] Liu S., Marcelin G., Blouet C., Jeong J.H., Jo Y.H., Schwartz G.J., Chua S. (2018). A gut-brain axis regulating glucose metabolism mediated by bile acids and competitive fibroblast growth factor actions at the hypothalamus. Mol. Metab..

[176] Ryan K.K., Kohli R., Gutierrez-Aguilar R., Gaitonde S.G., Woods S.C., Seeley R.J. (2013). Fibroblast growth factor-19 action in the brain reduces food intake and body weight and improves glucose tolerance in male rats. Endocrinology.

[177] Morton G.J., Matsen M.E., Bracy D.P., Meek T.H., Nguyen H.T., Stefanovski D., Bergman R.N., Wasserman D.H., Schwartz M.W. (2013). FGF19 action in the brain induces insulin-independent glucose lowering. J. Clin. Investig..

[178] Lan T., Morgan D.A., Rahmouni K., Sonoda J., Fu X., Burgess S.C., Holland W.L., Kliewer S.A., Mangelsdorf D.J. (2017). FGF19, FGF21, and an FGFR1/β-Klotho-Activating Antibody Act on the Nervous System to Regulate Body Weight and Glycemia. Cell Metab..

[179] Zhou X., Cao L., Jiang C., Xie Y., Cheng X., Krausz K.W., Qi Y., Sun L., Shah Y.M., Gonzalez F.J. (2014). PPARα-UGT axis activation represses intestinal FXR-FGF15 feedback signalling and exacerbates experimental colitis. Nat. Commun..

[180] Okamura A., Koyanagi S., Dilxiat A., Kusunose N., Chen J.J., Matsunaga N., Shibata S., Ohdo S. (2014). Bile acid-regulated peroxisome proliferator-activated receptor-α (PPARα) activity underlies circadian expression of intestinal peptide absorption transporter PepT1/Slc15a1. J. Biol. Chem..

[181] Overbergh L., Stoffels K., Waer M., Verstuyf A., Bouillon R., Mathieu C. (2006). Immune regulation of 25-hydroxyvitamin d-1α-hydroxylase in human monocytic THP1 cells: Mechanisms of interferon-γ-mediated induction. J. Clin. Endocrinol. Metab..

[182] Overbergh L., Decallonne B., Valckx D., Verstuyf A., Depovere J., Laureys J., Rutgeerts O., Saint-Arnaud R., Bouillon R., Mathieu C. (2000). Identification and immune regulation of 25-hydroxyvitamin d-1-α-hydroxylase in murine macrophages. Clin. Exp. Immunol..

[183] Segersten U., Holm P.K., Bjorklund P., Hessman O., Nordgren H., Binderup L., Akerstrom G., Hellman P., Westin G. (2005). 25-Hydroxyvitamin D_3_ 1α-hydroxylase expression in breast cancer and use of non-1α-hydroxylated vitamin D analogue. Breast Cancer Res..

[184] McCarthy K., Laban C., Bustin S.A., Ogunkolade W., Khalaf S., Carpenter R., Jenkins P.J. (2009). Expression of 25-hydroxyvitamin d-1-α-hydroxylase, and vitamin D receptor mRNA in normal and malignant breast tissue. Anticancer Res..

[185] Tangpricha V., Flanagan J.N., Whitlatch L.W., Tseng C.C., Chen T.C., Holt P.R., Lipkin M.S., Holick M.F. (2001). 25-hydroxyvitamin d-1α-hydroxylase in normal and malignant colon tissue. Lancet.

[186] Garland C.F., Kim J.J., Mohr S.B., Gorham E.D., Grant W.B., Giovannucci E.L., Baggerly L., Hofflich H., Ramsdell J.W., Zeng K. (2014). Meta-analysis of all-cause mortality according to serum 25-hydroxyvitamin D. Am. J. Public Health.

[187] Chapuy M.C., Preziosi P., Maamer M., Arnaud S., Galan P., Hercberg S., Meunier P.J. (1997). Prevalence of vitamin D insufficiency in an adult normal population. Osteoporos. Int..

[188] Chatterjee M. (2001). Vitamin D and genomic stability. Mutat. Res..

[189] Schwalfenberg G.K. (2011). A review of the critical role of vitamin D in the functioning of the immune system and the clinical implications of vitamin D deficiency. Mol. Nutr. Food Res..

[190] Amrein K., Quraishi S.A., Litonjua A.A., Gibbons F.K., Pieber T.R., Camargo C.A., Giovannucci E., Christopher K.B. (2014). Evidence for a U-shaped relationship between prehospital vitamin D status and mortality: A cohort study. J. Clin. Endocrinol. Metab..

[191] Wu-Wong J.R., Chen Y.W., Nakane M., Wolf M. (2011). Differential effects of vitamin d receptor agonists on gene expression in neonatal rat cardiomyocytes. Cardiovasc. Drugs Ther..

[192] Adachi R., Honma Y., Masuno H., Kawana K., Shimomura I., Yamada S., Makishima M. (2005). Selective activation of vitamin D receptor by lithocholic acid acetate, a bile acid derivative. J. Lipid Res..

[193] Haussler M.R., Haussler C.A., Bartik L., Whitfield G.K., Hsieh J.C., Slater S., Jurutka P.W. (2008). Vitamin D receptor: Molecular signaling and actions of nutritional ligands in disease prevention. Nutr. Rev..

[194] Moon J. (1994). The role of vitamin D in toxic metal absorption: A review. J. Am. Coll. Nutr..

[195] Eyles D.W., Liu P.Y., Josh P., Cui X. (2014). Intracellular distribution of the vitamin D receptor in the brain: Comparison with classic target tissues and redistribution with development. Neuroscience.

[196] Wang Y., Zhu J., DeLuca H.F. (2012). Where is the vitamin D receptor?. Arch. Biochem. Biophys..

[197] Prufer K., Veenstra T.D., Jirikowski G.F., Kumar R. (1999). Distribution of 1,25-dihydroxyvitamin D_3_ receptor immunoreactivity in the rat brain and spinal cord. J. Chem. Neuroanat..

[198] Pardridge W.M., Sakiyama R., Coty W.A. (1985). Restricted transport of vitamin D and A derivatives through the rat blood-brain barrier. J. Neurochem..

[199] Fu G.K., Lin D., Zhang M.Y., Bikle D.D., Shackleton C.H., Miller W.L., Portale A.A. (1997). Cloning of human 25-hydroxyvitamin d-1 α-hydroxylase and mutations causing vitamin D-dependent rickets type 1. Mol. Endocrinol..

[200] Eyles D.W., Smith S., Kinobe R., Hewison M., McGrath J.J. (2005). Distribution of the vitamin D receptor and 1 α-hydroxylase in human brain. J. Chem. Neuroanat..

[201] Du J., Wei X., Ge X., Chen Y., Li Y.C. (2017). Microbiota-Dependent Induction of Colonic Cyp27b1 Is Associated with Colonic Inflammation: Implications of Locally Produced 1,25-Dihydroxyvitamin D_3_ in Inflammatory Regulation in the Colon. Endocrinology.

[202] Pols T.W.H., Puchner T., Korkmaz H.I., Vos M., Soeters M.R., de Vries C.J.M. (2017). Lithocholic acid controls adaptive immune responses by inhibition of Th1 activation through the Vitamin D receptor. PLoS ONE.

[203] He L., Liu T., Shi Y., Tian F., Hu H., Deb D.K., Chen Y., Bissonnette M., Li Y.C. (2018). Gut Epithelial Vitamin D Receptor Regulates Microbiota-Dependent Mucosal Inflammation by Suppressing Intestinal Epithelial Cell Apoptosis. Endocrinology.

[204] Chen J., Waddell A., Lin Y.D., Cantorna M.T. (2015). Dysbiosis caused by vitamin D receptor deficiency confers colonization resistance to Citrobacter rodentium through modulation of innate lymphoid cells. Mucosal Immunol..

[205] Su D., Nie Y., Zhu A., Chen Z., Wu P., Zhang L., Luo M., Sun Q., Cai L., Lai Y. (2016). Vitamin D Signaling through Induction of Paneth Cell Defensins Maintains Gut Microbiota and Improves Metabolic Disorders and Hepatic Steatosis in Animal Models. Front. Physiol..

[206] Wu S., Zhang Y.G., Lu R., Xia Y., Zhou D., Petrof E.O., Claud E.C., Chen D., Chang E.B., Carmeliet G. (2015). Intestinal epithelial vitamin D receptor deletion leads to defective autophagy in colitis. Gut.

[207] Wang J., Thingholm L.B., Skieceviciene J., Rausch P., Kummen M., Hov J.R., Degenhardt F., Heinsen F.A., Ruhlemann M.C., Szymczak S. (2016). Genome-wide association analysis identifies variation in vitamin D receptor and other host factors influencing the gut microbiota. Nat. Genet..

[208] Liu P.T., Stenger S., Li H., Wenzel L., Tan B.H., Krutzik S.R., Ochoa M.T., Schauber J., Wu K., Meinken C. (2006). Toll-like receptor triggering of a vitamin D-mediated human antimicrobial response. Science.

[209] Liu P.T., Stenger S., Tang D.H., Modlin R.L. (2007). Cutting edge: Vitamin D-mediated human antimicrobial activity against Mycobacterium tuberculosis is dependent on the induction of cathelicidin. J. Immunol..

[210] Wu S., Yoon S., Zhang Y.G., Lu R., Xia Y., Wan J., Petrof E.O., Claud E.C., Chen D., Sun J. (2015). Vitamin D receptor pathway is required for probiotic protection in colitis. Am. J. Physiol. Gastrointest. Liver Physiol..

[211] Bora S.A., Kennett M.J., Smith P.B., Patterson A.D., Cantorna M.T. (2018). Regulation of vitamin D metabolism following disruption of the microbiota using broad spectrum antibiotics. J. Nutr. Biochem..

[212] Eyles D., Brown J., Mackay-Sim A., McGrath J., Feron F. (2003). Vitamin D_3_ and brain development. Neuroscience.

[213] Jiang P., Zhang L.H., Cai H.L., Li H.D., Liu Y.P., Tang M.M., Dang R.L., Zhu W.Y., Xue Y., He X. (2014). Neurochemical effects of chronic administration of calcitriol in rats. Nutrients.

[214] Smith M.P., Fletcher-Turner A., Yurek D.M., Cass W.A. (2006). Calcitriol protection against dopamine loss induced by intracerebroventricular administration of 6-hydroxydopamine. Neurochem. Res..

[215] Wang J.Y., Wu J.N., Cherng T.L., Hoffer B.J., Chen H.H., Borlongan C.V., Wang Y. (2001). Vitamin D_3_ attenuates 6-hydroxydopamine-induced neurotoxicity in rats. Brain Res..

[216] Nataf S., Garcion E., Darcy F., Chabannes D., Muller J.Y., Brachet P. (1996). 1,25 Dihydroxyvitamin D_3_ exerts regional effects in the central nervous system during experimental allergic encephalomyelitis. J. Neuropathol. Exp. Neurol..

[217] Garcion E., Sindji L., Montero-Menei C., Andre C., Brachet P., Darcy F. (1998). Expression of inducible nitric oxide synthase during rat brain inflammation: Regulation by 1,25-dihydroxyvitamin D_3_. Glia.

[218] Cannell J.J. (2008). Autism and vitamin D. Med. Hypotheses.

[219] Grant W.B., Soles C.M. (2009). Epidemiologic evidence supporting the role of maternal vitamin D deficiency as a risk factor for the development of infantile autism. Dermatoendocrinol.

[220] McGrath J. (1999). Hypothesis: Is low prenatal vitamin D a risk-modifying factor for schizophrenia?. Schizophr. Res..

[221] Kesby J.P., Burne T.H., McGrath J.J., Eyles D.W. (2006). Developmental vitamin D deficiency alters MK 801-induced hyperlocomotion in the adult rat: An animal model of schizophrenia. Biol. Psychiatry.

[222] Kesby J.P., Cui X., O’Loan J., McGrath J.J., Burne T.H., Eyles D.W. (2010). Developmental vitamin D deficiency alters dopamine-mediated behaviors and dopamine transporter function in adult female rats. Psychopharmacology.

[223] Munger K.L., Zhang S.M., O’Reilly E., Hernan M.A., Olek M.J., Willett W.C., Ascherio A. (2004). Vitamin D intake and incidence of multiple sclerosis. Neurology.

[224] Munger K.L., Levin L.I., Hollis B.W., Howard N.S., Ascherio A. (2006). Serum 25-hydroxyvitamin D levels and risk of multiple sclerosis. JAMA.

[225] Evatt M.L., Delong M.R., Khazai N., Rosen A., Triche S., Tangpricha V. (2008). Prevalence of vitamin D insufficiency in patients with Parkinson disease and Alzheimer disease. Arch. Neurol..

[226] Hahn M.E., Karchner S.I., Shapiro M.A., Perera S.A. (1997). Molecular evolution of two vertebrate aryl hydrocarbon (dioxin) receptors (AHR1 and AHR2) and the PAS family. Proc. Natl. Acad. Sci. USA.

[227] Diani-Moore S., Ram P., Li X., Mondal P., Youn D.Y., Sauve A.A., Rifkind A.B. (2010). Identification of the aryl hydrocarbon receptor target gene TiPARP as a mediator of suppression of hepatic gluconeogenesis by 2,3,7,8-tetrachlorodibenzo-*p*-dioxin and of nicotinamide as a corrective agent for this effect. J. Biol. Chem..

[228] Zhang S., Qin C., Safe S.H. (2003). Flavonoids as aryl hydrocarbon receptor agonists/antagonists: Effects of structure and cell context. Environ. Health Perspect..

[229] Hammerschmidt-Kamper C., Biljes D., Merches K., Steiner I., Daldrup T., Bol-Schoenmakers M., Pieters R.H.H., Esser C. (2017). Indole-3-carbinol, a plant nutrient and AhR-Ligand precursor, supports oral tolerance against OVA and improves peanut allergy symptoms in mice. PLoS ONE.

[230] Busbee P.B., Rouse M., Nagarkatti M., Nagarkatti P.S. (2013). Use of natural AhR ligands as potential therapeutic modalities against inflammatory disorders. Nutr. Rev..

[231] Mezrich J.D., Fechner J.H., Zhang X., Johnson B.P., Burlingham W.J., Bradfield C.A. (2010). An interaction between kynurenine and the aryl hydrocarbon receptor can generate regulatory T cells. J. Immunol..

[232] DiNatale B.C., Murray I.A., Schroeder J.C., Flaveny C.A., Lahoti T.S., Laurenzana E.M., Omiecinski C.J., Perdew G.H. (2010). Kynurenic acid is a potent endogenous aryl hydrocarbon receptor ligand that synergistically induces interleukin-6 in the presence of inflammatory signaling. Toxicol. Sci..

[233] Hubbard T.D., Murray I.A., Bisson W.H., Lahoti T.S., Gowda K., Amin S.G., Patterson A.D., Perdew G.H. (2015). Adaptation of the human aryl hydrocarbon receptor to sense microbiota-derived indoles. Sci. Rep..

[234] Jin U.H., Lee S.O., Sridharan G., Lee K., Davidson L.A., Jayaraman A., Chapkin R.S., Alaniz R., Safe S. (2014). Microbiome-derived tryptophan metabolites and their aryl hydrocarbon receptor-dependent agonist and antagonist activities. Mol. Pharmacol..

[235] Moura-Alves P., Fae K., Houthuys E., Dorhoi A., Kreuchwig A., Furkert J., Barison N., Diehl A., Munder A., Constant P. (2014). AhR sensing of bacterial pigments regulates antibacterial defence. Nature.

[236] Jin U.H., Cheng Y., Park H., Davidson L.A., Callaway E.S., Chapkin R.S., Jayaraman A., Asante A., Allred C., Weaver E.A. (2017). Short Chain Fatty Acids Enhance Aryl Hydrocarbon (Ah) Responsiveness in Mouse Colonocytes and Caco-2 Human Colon Cancer Cells. Sci. Rep..

[237] Gandhi R., Kumar D., Burns E.J., Nadeau M., Dake B., Laroni A., Kozoriz D., Weiner H.L., Quintana F.J. (2010). Activation of the aryl hydrocarbon receptor induces human type 1 regulatory T cell-like and Foxp3^+^ regulatory T cells. Nat. Immunol..

[238] Quintana F.J., Murugaiyan G., Farez M.F., Mitsdoerffer M., Tukpah A.M., Burns E.J., Weiner H.L. (2010). An endogenous aryl hydrocarbon receptor ligand acts on dendritic cells and T cells to suppress experimental autoimmune encephalomyelitis. Proc. Natl. Acad. Sci. USA.

[239] Veldhoen M., Hirota K., Christensen J., O’Garra A., Stockinger B. (2009). Natural agonists for aryl hydrocarbon receptor in culture medium are essential for optimal differentiation of Th17 T cells. J. Exp. Med..

[240] Veldhoen M., Hirota K., Westendorf A.M., Buer J., Dumoutier L., Renauld J.C., Stockinger B. (2008). The aryl hydrocarbon receptor links TH17-cell-mediated autoimmunity to environmental toxins. Nature.

[241] Quintana F.J., Basso A.S., Iglesias A.H., Korn T., Farez M.F., Bettelli E., Caccamo M., Oukka M., Weiner H.L. (2008). Control of T_reg_ and T_H_17 cell differentiation by the aryl hydrocarbon receptor. Nature.

[242] Duarte J.H., Di Meglio P., Hirota K., Ahlfors H., Stockinger B. (2013). Differential influences of the aryl hydrocarbon receptor on Th17 mediated responses in vitro and in vivo. PLoS ONE.

[243] Nguyen N.T., Hanieh H., Nakahama T., Kishimoto T. (2013). The roles of aryl hydrocarbon receptor in immune responses. Int. Immunol..

[244] Stockinger B., Di Meglio P., Gialitakis M., Duarte J.H. (2014). The aryl hydrocarbon receptor: Multitasking in the immune system. Annu. Rev. Immunol..

[245] Chng S.H., Kundu P., Dominguez-Brauer C., Teo W.L., Kawajiri K., Fujii-Kuriyama Y., Mak T.W., Pettersson S. (2016). Ablating the aryl hydrocarbon receptor (AhR) in CD11c+ cells perturbs intestinal epithelium development and intestinal immunity. Sci. Rep..

[246] Korecka A., Dona A., Lahiri S., Tett A.J., Al-Asmakh M., Braniste V., D’Arienzo R., Abbaspour A., Reichardt N., Fujii-Kuriyama Y. (2016). Bidirectional communication between the Aryl hydrocarbon Receptor (AhR) and the microbiome tunes host metabolism. NPJ Biofilms Microbiomes.

[247] Lamas B., Richard M.L., Leducq V., Pham H.P., Michel M.L., Da Costa G., Bridonneau C., Jegou S., Hoffmann T.W., Natividad J.M. (2016). CARD9 impacts colitis by altering gut microbiota metabolism of tryptophan into aryl hydrocarbon receptor ligands. Nat. Med..

[248] Le May C., Pineau T., Bigot K., Kohl C., Girard J., Pegorier J.P. (2000). Reduced hepatic fatty acid oxidation in fasting PPARα null mice is due to impaired mitochondrial hydroxymethylglutaryl-CoA synthase gene expression. FEBS Lett..

[249] Shaban Z., Soliman M., El-Shazly S., El-Bohi K., Abdelazeez A., Kehelo K., Kim H.S., Muzandu K., Ishizuka M., Kazusaka A. (2005). AhR and PPARα: Antagonistic effects on CYP2B and CYP3A, and additive inhibitory effects on CYP2C11. Xenobiotica.

[250] Lee J.H., Wada T., Febbraio M., He J., Matsubara T., Lee M.J., Gonzalez F.J., Xie W. (2010). A novel role for the dioxin receptor in fatty acid metabolism and hepatic steatosis. Gastroenterology.

[251] Lu P., Yan J., Liu K., Garbacz W.G., Wang P., Xu M., Ma X., Xie W. (2015). Activation of aryl hydrocarbon receptor dissociates fatty liver from insulin resistance by inducing fibroblast growth factor 21. Hepatology.

[252] Remillard R.B., Bunce N.J. (2002). Linking dioxins to diabetes: Epidemiology and biologic plausibility. Environ. Health Perspect..

[253] Villard P.H., Caverni S., Baanannou A., Khalil A., Martin P.G., Penel C., Pineau T., Seree E., Barra Y. (2007). PPARα transcriptionally induces AhR expression in Caco-2, but represses AhR pro-inflammatory effects. Biochem. Biophys. Res. Commun..

[254] Fallone F., Villard P.H., Decome L., Seree E., Meo M., Chacon C., Durand A., Barra Y., Lacarelle B. (2005). PPARα activation potentiates AhR-induced CYP1A1 expression. Toxicology.

[255] Dutchak P.A., Katafuchi T., Bookout A.L., Choi J.H., Yu R.T., Mangelsdorf D.J., Kliewer S.A. (2012). Fibroblast growth factor-21 regulates PPARγ activity and the antidiabetic actions of thiazolidinediones. Cell.

[256] Oishi K., Tomita T. (2011). Thiazolidinediones are potent inducers of fibroblast growth factor 21 expression in the liver. Biol. Pharm. Bull..

[257] Inagaki T., Dutchak P., Zhao G., Ding X., Gautron L., Parameswara V., Li Y., Goetz R., Mohammadi M., Esser V. (2007). Endocrine regulation of the fasting response by PPARα-mediated induction of fibroblast growth factor 21. Cell Metab..

[258] Liang Q., Zhong L., Zhang J., Wang Y., Bornstein S.R., Triggle C.R., Ding H., Lam K.S., Xu A. (2014). FGF21 maintains glucose homeostasis by mediating the cross talk between liver and brain during prolonged fasting. Diabetes.

[259] Coskun T., Bina H.A., Schneider M.A., Dunbar J.D., Hu C.C., Chen Y., Moller D.E., Kharitonenkov A. (2008). Fibroblast growth factor 21 corrects obesity in mice. Endocrinology.

[260] Kharitonenkov A., Shiyanova T.L., Koester A., Ford A.M., Micanovic R., Galbreath E.J., Sandusky G.E., Hammond L.J., Moyers J.S., Owens R.A. (2005). FGF-21 as a novel metabolic regulator. J. Clin. Investig..

[261] Owen B.M., Ding X., Morgan D.A., Coate K.C., Bookout A.L., Rahmouni K., Kliewer S.A., Mangelsdorf D.J. (2014). FGF21 acts centrally to induce sympathetic nerve activity, energy expenditure, and weight loss. Cell Metab..

[262] Bookout A.L., de Groot M.H., Owen B.M., Lee S., Gautron L., Lawrence H.L., Ding X., Elmquist J.K., Takahashi J.S., Mangelsdorf D.J. (2013). FGF21 regulates metabolism and circadian behavior by acting on the nervous system. Nat. Med..

[263] Iroz A., Montagner A., Benhamed F., Levavasseur F., Polizzi A., Anthony E., Regnier M., Fouche E., Lukowicz C., Cauzac M. (2017). A Specific ChREBP and PPARα Cross-Talk Is Required for the Glucose-Mediated FGF21 Response. Cell Rep..

[264] Talukdar S., Owen B.M., Song P., Hernandez G., Zhang Y., Zhou Y., Scott W.T., Paratala B., Turner T., Smith A. (2016). FGF21 Regulates Sweet and Alcohol Preference. Cell Metab..

[265] Song P., Zechner C., Hernandez G., Canovas J., Xie Y., Sondhi V., Wagner M., Stadlbauer V., Horvath A., Leber B. (2018). The Hormone FGF21 Stimulates Water Drinking in Response to Ketogenic Diet and Alcohol. Cell Metab..

[266] Filbrandt C.R., Wu Z., Zlokovic B., Opanashuk L., Gasiewicz T.A. (2004). Presence and functional activity of the aryl hydrocarbon receptor in isolated murine cerebral vascular endothelial cells and astrocytes. Neurotoxicology.

[267] Jacob A., Hartz A.M., Potin S., Coumoul X., Yousif S., Scherrmann J.M., Bauer B., Decleves X. (2011). Aryl hydrocarbon receptor-dependent upregulation of Cyp1b1 by TCDD and diesel exhaust particles in rat brain microvessels. Fluids Barriers CNS.

[268] Qin H., Powell-Coffman J.A. (2004). The Caenorhabditis elegans aryl hydrocarbon receptor, AHR-1, regulates neuronal development. Dev. Biol..

[269] Huang X., Powell-Coffman J.A., Jin Y. (2004). The AHR-1 aryl hydrocarbon receptor and its co-factor the AHA-1 aryl hydrocarbon receptor nuclear translocator specify GABAergic neuron cell fate in *C. elegans*. Development.

[270] Hill A., Howard C.V., Strahle U., Cossins A. (2003). Neurodevelopmental defects in zebrafish (*Danio rerio*) at environmentally relevant dioxin (TCDD) concentrations. Toxicol. Sci..

[271] Gohlke J.M., Stockton P.S., Sieber S., Foley J., Portier C.J. (2009). AhR-mediated gene expression in the developing mouse telencephalon. Reprod. Toxicol..

[272] Williamson M.A., Gasiewicz T.A., Opanashuk L.A. (2005). Aryl hydrocarbon receptor expression and activity in cerebellar granule neuroblasts: Implications for development and dioxin neurotoxicity. Toxicol. Sci..

[273] Kimura E., Ding Y., Tohyama C. (2016). AhR signaling activation disrupts migration and dendritic growth of olfactory interneurons in the developing mouse. Sci. Rep..

[274] Latchney S.E., Hein A.M., O’Banion M.K., DiCicco-Bloom E., Opanashuk L.A. (2013). Deletion or activation of the aryl hydrocarbon receptor alters adult hippocampal neurogenesis and contextual fear memory. J. Neurochem..

[275] Qin H., Zhai Z., Powell-Coffman J.A. (2006). The Caenorhabditis elegans AHR-1 transcription complex controls expression of soluble guanylate cyclase genes in the URX neurons and regulates aggregation behavior. Dev. Biol..

[276] Pocivavsek A., Wu H.Q., Elmer G.I., Bruno J.P., Schwarcz R. (2012). Pre- and postnatal exposure to kynurenine causes cognitive deficits in adulthood. Eur. J. Neurosci..

[277] Wang X., Hawkins B.T., Miller D.S. (2011). Aryl hydrocarbon receptor-mediated up-regulation of ATP-driven xenobiotic efflux transporters at the blood-brain barrier. FASEB J..

[278] Wang X., Hawkins B.T., Miller D.S. (2011). Activating PKC-β1 at the blood-brain barrier reverses induction of P-glycoprotein activity by dioxin and restores drug delivery to the CNS. J. Cereb. Blood Flow Metab..

[279] Braniste V., Al-Asmakh M., Kowal C., Anuar F., Abbaspour A., Toth M., Korecka A., Bakocevic N., Ng L.G., Kundu P. (2014). The gut microbiota influences blood-brain barrier permeability in mice. Sci. Transl. Med..

[280] Yu M., Wang Q., Ma Y., Li L., Yu K., Zhang Z., Chen G., Li X., Xiao W., Xu P. (2018). Aryl Hydrocarbon Receptor Activation Modulates Intestinal Epithelial Barrier Function by Maintaining Tight Junction Integrity. Int. J. Biol. Sci..

[281] Andrysik Z., Prochazkova J., Kabatkova M., Umannova L., Simeckova P., Kohoutek J., Kozubik A., Machala M., Vondracek J. (2013). Aryl hydrocarbon receptor-mediated disruption of contact inhibition is associated with connexin43 downregulation and inhibition of gap junctional intercellular communication. Arch. Toxicol..

[282] Ezan P., Andre P., Cisternino S., Saubamea B., Boulay A.C., Doutremer S., Thomas M.A., Quenech’du N., Giaume C., Cohen-Salmon M. (2012). Deletion of astroglial connexins weakens the blood-brain barrier. J. Cereb. Blood Flow Metab..

[283] Benson J.M., Shepherd D.M. (2011). Dietary ligands of the aryl hydrocarbon receptor induce anti-inflammatory and immunoregulatory effects on murine dendritic cells. Toxicol. Sci..

[284] Lee Y.H., Lin C.H., Hsu P.C., Sun Y.Y., Huang Y.J., Zhuo J.H., Wang C.Y., Gan Y.L., Hung C.C., Kuan C.Y. (2015). Aryl hydrocarbon receptor mediates both proinflammatory and anti-inflammatory effects in lipopolysaccharide-activated microglia. Glia.

[285] Jung H.J., Nam K.N., Son M.S., Kang H., Hong J.W., Kim J.W., Lee E.H. (2011). Indirubin-3′-oxime inhibits inflammatory activation of rat brain microglia. Neurosci. Lett..

[286] Rothhammer V., Mascanfroni I.D., Bunse L., Takenaka M.C., Kenison J.E., Mayo L., Chao C.C., Patel B., Yan R., Blain M. (2016). Type I interferons and microbial metabolites of tryptophan modulate astrocyte activity and central nervous system inflammation via the aryl hydrocarbon receptor. Nat. Med..

[287] Sofroniew M.V. (2015). Astrocyte barriers to neurotoxic inflammation. Nat. Rev. Neurosci..

[288] Ma Y., Liu D. (2012). Activation of pregnane X receptor by pregnenolone 16 α-carbonitrile prevents high-fat diet-induced obesity in AKR/J mice. PLoS ONE.

[289] Konno Y., Negishi M., Kodama S. (2008). The roles of nuclear receptors CAR and PXR in hepatic energy metabolism. Drug Metab. Pharmacokinet..

[290] Kliewer S.A., Goodwin B., Willson T.M. (2002). The nuclear pregnane X receptor: A key regulator of xenobiotic metabolism. Endocr. Rev..

[291] Mencarelli A., Migliorati M., Barbanti M., Cipriani S., Palladino G., Distrutti E., Renga B., Fiorucci S. (2010). Pregnane-X-receptor mediates the anti-inflammatory activities of rifaximin on detoxification pathways in intestinal epithelial cells. Biochem. Pharmacol..

[292] Lamba V., Yasuda K., Lamba J.K., Assem M., Davila J., Strom S., Schuetz E.G. (2004). PXR (NR1I2): Splice variants in human tissues, including brain, and identification of neurosteroids and nicotine as PXR activators. Toxicol. Appl. Pharmacol..

[293] Fukuen S., Fukuda T., Matsuda H., Sumida A., Yamamoto I., Inaba T., Azuma J. (2002). Identification of the novel splicing variants for the hPXR in human livers. Biochem. Biophys. Res. Commun..

[294] Gray M.A., Pollock C.B., Schook L.B., Squires E.J. (2010). Characterization of porcine pregnane X receptor, farnesoid X receptor and their splice variants. Exp. Biol. Med..

[295] Staudinger J.L., Goodwin B., Jones S.A., Hawkins-Brown D., MacKenzie K.I., LaTour A., Liu Y., Klaassen C.D., Brown K.K., Reinhard J. (2001). The nuclear receptor PXR is a lithocholic acid sensor that protects against liver toxicity. Proc. Natl. Acad. Sci. USA.

[296] Maglich J.M., Stoltz C.M., Goodwin B., Hawkins-Brown D., Moore J.T., Kliewer S.A. (2002). Nuclear pregnane x receptor and constitutive androstane receptor regulate overlapping but distinct sets of genes involved in xenobiotic detoxification. Mol. Pharmacol..

[297] Venkatesh M., Mukherjee S., Wang H., Li H., Sun K., Benechet A.P., Qiu Z., Maher L., Redinbo M.R., Phillips R.S. (2014). Symbiotic bacterial metabolites regulate gastrointestinal barrier function via the xenobiotic sensor PXR and Toll-like receptor 4. Immunity.

[298] Chrencik J.E., Orans J., Moore L.B., Xue Y., Peng L., Collins J.L., Wisely G.B., Lambert M.H., Kliewer S.A., Redinbo M.R. (2005). Structural disorder in the complex of human pregnane X receptor and the macrolide antibiotic rifampicin. Mol. Endocrinol..

[299] Xie W., Radominska-Pandya A., Shi Y., Simon C.M., Nelson M.C., Ong E.S., Waxman D.J., Evans R.M. (2001). An essential role for nuclear receptors SXR/PXR in detoxification of cholestatic bile acids. Proc. Natl. Acad. Sci. USA.

[300] Javitt N.B. (1966). Cholestasis in rats induced by taurolithocholate. Nature.

[301] Selye H. (1972). Prevention by catatoxic steroids of lithocholic acid-induced biliary concrements in the rat. Proc. Soc. Exp. Biol. Med..

[302] Dring M.M., Goulding C.A., Trimble V.I., Keegan D., Ryan A.W., Brophy K.M., Smyth C.M., Keeling P.W., O’Donoghue D., O’Sullivan M. (2006). The pregnane X receptor locus is associated with susceptibility to inflammatory bowel disease. Gastroenterology.

[303] Garg A., Zhao A., Erickson S.L., Mukherjee S., Lau A.J., Alston L., Chang T.K., Mani S., Hirota S.A. (2016). Pregnane X Receptor Activation Attenuates Inflammation-Associated Intestinal Epithelial Barrier Dysfunction by Inhibiting Cytokine-Induced Myosin Light-Chain Kinase Expression and c-Jun N-Terminal Kinase 1/2 Activation. J. Pharmacol. Exp. Ther..

[304] Terc J., Hansen A., Alston L., Hirota S.A. (2014). Pregnane X receptor agonists enhance intestinal epithelial wound healing and repair of the intestinal barrier following the induction of experimental colitis. Eur. J. Pharm. Sci..

[305] Mencarelli A., Renga B., Palladino G., Claudio D., Ricci P., Distrutti E., Barbanti M., Baldelli F., Fiorucci S. (2011). Inhibition of NF-κB by a PXR-dependent pathway mediates counter-regulatory activities of rifaximin on innate immunity in intestinal epithelial cells. Eur. J. Pharmacol..

[306] Dou W., Zhang J., Zhang E., Sun A., Ding L., Chou G., Wang Z., Mani S. (2013). Chrysin ameliorates chemically induced colitis in the mouse through modulation of a PXR/NF-κB signaling pathway. J. Pharmacol. Exp. Ther..

[307] Boussadia B., Lakhal L., Payrastre L., Ghosh C., Pascussi J.M., Gangarossa G., Marchi N. (2018). Pregnane X Receptor Deletion Modifies Recognition Memory and Electroencephalographic Activity. Neuroscience.

[308] Bauer B., Yang X., Hartz A.M., Olson E.R., Zhao R., Kalvass J.C., Pollack G.M., Miller D.S. (2006). In vivo activation of human pregnane X receptor tightens the blood-brain barrier to methadone through P-glycoprotein up-regulation. Mol. Pharmacol..

[309] Pascussi J.M., Busson-Le Coniat M., Maurel P., Vilarem M.J. (2003). Transcriptional analysis of the orphan nuclear receptor constitutive androstane receptor (NR1I3) gene promoter: Identification of a distal glucocorticoid response element. Mol. Endocrinol..

[310] Patel R.D., Hollingshead B.D., Omiecinski C.J., Perdew G.H. (2007). Aryl-hydrocarbon receptor activation regulates constitutive androstane receptor levels in murine and human liver. Hepatology.

[311] Ding X., Lichti K., Kim I., Gonzalez F.J., Staudinger J.L. (2006). Regulation of constitutive androstane receptor and its target genes by fasting, cAMP, hepatocyte nuclear factor α, and the coactivator peroxisome proliferator-activated receptor γ coactivator-1α. J. Biol. Chem..

[312] Mutoh S., Sobhany M., Moore R., Perera L., Pedersen L., Sueyoshi T., Negishi M. (2013). Phenobarbital indirectly activates the constitutive active androstane receptor (CAR) by inhibition of epidermal growth factor receptor signaling. Sci. Signal..

[313] Lundin A., Bok C.M., Aronsson L., Bjorkholm B., Gustafsson J.A., Pott S., Arulampalam V., Hibberd M., Rafter J., Pettersson S. (2008). Gut flora, Toll-like receptors and nuclear receptors: A tripartite communication that tunes innate immunity in large intestine. Cell. Microbiol..

[314] Assenat E., Gerbal-Chaloin S., Larrey D., Saric J., Fabre J.M., Maurel P., Vilarem M.J., Pascussi J.M. (2004). Interleukin 1β inhibits CAR-induced expression of hepatic genes involved in drug and bilirubin clearance. Hepatology.

[315] Kawamoto T., Kakizaki S., Yoshinari K., Negishi M. (2000). Estrogen activation of the nuclear orphan receptor CAR (constitutive active receptor) in induction of the mouse Cyp2b10 gene. Mol. Endocrinol..

[316] Banerjee M., Robbins D., Chen T. (2013). Modulation of xenobiotic receptors by steroids. Molecules.

[317] Bjorkholm B., Bok C.M., Lundin A., Rafter J., Hibberd M.L., Pettersson S. (2009). Intestinal microbiota regulate xenobiotic metabolism in the liver. PLoS ONE.

[318] Kawamoto T., Sueyoshi T., Zelko I., Moore R., Washburn K., Negishi M. (1999). Phenobarbital-responsive nuclear translocation of the receptor CAR in induction of the CYP2B gene. Mol. Cell. Biol..

[319] Honkakoski P., Zelko I., Sueyoshi T., Negishi M. (1998). The nuclear orphan receptor CAR-retinoid X receptor heterodimer activates the phenobarbital-responsive enhancer module of the CYP2B gene. Mol. Cell. Biol..

[320] Auerbach S.S., Ramsden R., Stoner M.A., Verlinde C., Hassett C., Omiecinski C.J. (2003). Alternatively spliced isoforms of the human constitutive androstane receptor. Nucleic Acids Res..

[321] Koyano S., Kurose K., Saito Y., Ozawa S., Hasegawa R., Komamura K., Ueno K., Kamakura S., Kitakaze M., Nakajima T. (2004). Functional characterization of four naturally occurring variants of human pregnane X receptor (PXR): One variant causes dramatic loss of both DNA binding activity and the transactivation of the CYP3A4 promoter/enhancer region. Drug Metab. Dispos..

[322] Lamba J.K., Lamba V., Yasuda K., Lin Y.S., Assem M., Thompson E., Strom S., Schuetz E. (2004). Expression of constitutive androstane receptor splice variants in human tissues and their functional consequences. J. Pharmacol. Exp. Ther..

[323] Lin Y.S., Yasuda K., Assem M., Cline C., Barber J., Li C.W., Kholodovych V., Ai N., Chen J.D., Welsh W.J. (2009). The major human pregnane X receptor (PXR) splice variant, PXR.2, exhibits significantly diminished ligand-activated transcriptional regulation. Drug Metab. Dispos..

[324] Wallace B.D., Redinbo M.R. (2013). Xenobiotic-sensing nuclear receptors involved in drug metabolism: A structural perspective. Drug Metab. Rev..

[325] Dussault I., Lin M., Hollister K., Fan M., Termini J., Sherman M.A., Forman B.M. (2002). A structural model of the constitutive androstane receptor defines novel interactions that mediate ligand-independent activity. Mol. Cell. Biol..

[326] Jyrkkarinne J., Windshugel B., Ronkko T., Tervo A.J., Kublbeck J., Lahtela-Kakkonen M., Sippl W., Poso A., Honkakoski P. (2008). Insights into ligand-elicited activation of human constitutive androstane receptor based on novel agonists and three-dimensional quantitative structure-activity relationship. J. Med. Chem..

[327] Yoshinari K., Kobayashi K., Moore R., Kawamoto T., Negishi M. (2003). Identification of the nuclear receptor CAR:HSP90 complex in mouse liver and recruitment of protein phosphatase 2A in response to phenobarbital. FEBS Lett..

[328] Chen S., Wang K., Wan Y.J. (2010). Retinoids activate RXR/CAR-mediated pathway and induce CYP3A. Biochem. Pharmacol..

[329] Koike C., Moore R., Negishi M. (2005). Localization of the nuclear receptor CAR at the cell membrane of mouse liver. FEBS Lett..

[330] Moore L.B., Parks D.J., Jones S.A., Bledsoe R.K., Consler T.G., Stimmel J.B., Goodwin B., Liddle C., Blanchard S.G., Willson T.M. (2000). Orphan nuclear receptors constitutive androstane receptor and pregnane X receptor share xenobiotic and steroid ligands. J. Biol. Chem..

[331] Goodwin B., Hodgson E., D’Costa D.J., Robertson G.R., Liddle C. (2002). Transcriptional regulation of the human CYP3A4 gene by the constitutive androstane receptor. Mol. Pharmacol..

[332] Wortham M., Czerwinski M., He L., Parkinson A., Wan Y.J. (2007). Expression of constitutive androstane receptor, hepatic nuclear factor 4 α, and P450 oxidoreductase genes determines interindividual variability in basal expression and activity of a broad scope of xenobiotic metabolism genes in the human liver. Drug Metab. Dispos..

[333] Wang H., Faucette S., Sueyoshi T., Moore R., Ferguson S., Negishi M., LeCluyse E.L. (2003). A novel distal enhancer module regulated by pregnane X receptor/constitutive androstane receptor is essential for the maximal induction of CYP2B6 gene expression. J. Biol. Chem..

[334] Ueda A., Hamadeh H.K., Webb H.K., Yamamoto Y., Sueyoshi T., Afshari C.A., Lehmann J.M., Negishi M. (2002). Diverse roles of the nuclear orphan receptor CAR in regulating hepatic genes in response to phenobarbital. Mol. Pharmacol..

[335] Ferguson S.S., LeCluyse E.L., Negishi M., Goldstein J.A. (2002). Regulation of human CYP2C9 by the constitutive androstane receptor: Discovery of a new distal binding site. Mol. Pharmacol..

[336] Gerbal-Chaloin S., Daujat M., Pascussi J.M., Pichard-Garcia L., Vilarem M.J., Maurel P. (2002). Transcriptional regulation of CYP2C9 gene. Role of glucocorticoid receptor and constitutive androstane receptor. J. Biol. Chem..

[337] Sugatani J., Kojima H., Ueda A., Kakizaki S., Yoshinari K., Gong Q.H., Owens I.S., Negishi M., Sueyoshi T. (2001). The phenobarbital response enhancer module in the human bilirubin UDP-glucuronosyltransferase UGT1A1 gene and regulation by the nuclear receptor CAR. Hepatology.

[338] Gao J., He J., Zhai Y., Wada T., Xie W. (2009). The constitutive androstane receptor is an anti-obesity nuclear receptor that improves insulin sensitivity. J. Biol. Chem..

[339] Yarushkin A.A., Kachaylo E.M., Pustylnyak V.O. (2013). The constitutive androstane receptor activator 4-[(4*R*,6*R*)-4,6-diphenyl-1,3-dioxan-2-yl]-*N*,*N*-dimethylaniline inhibits the gluconeogenic genes PEPCK and G6Pase through the suppression of HNF4α and FOXO1 transcriptional activity. Br. J. Pharmacol..

[340] Kachaylo E.M., Yarushkin A.A., Pustylnyak V.O. (2012). Constitutive androstane receptor activation by 2,4,6-triphenyldioxane-1,3 suppresses the expression of the gluconeogenic genes. Eur. J. Pharmacol..

[341] Roth A., Looser R., Kaufmann M., Blattler S.M., Rencurel F., Huang W., Moore D.D., Meyer U.A. (2008). Regulatory cross-talk between drug metabolism and lipid homeostasis: Constitutive androstane receptor and pregnane X receptor increase Insig-1 expression. Mol. Pharmacol..

[342] Nakamura K., Moore R., Negishi M., Sueyoshi T. (2007). Nuclear pregnane X receptor cross-talk with FoxA2 to mediate drug-induced regulation of lipid metabolism in fasting mouse liver. J. Biol. Chem..

[343] Maglich J.M., Watson J., McMillen P.J., Goodwin B., Willson T.M., Moore J.T. (2004). The nuclear receptor CAR is a regulator of thyroid hormone metabolism during caloric restriction. J. Biol. Chem..

[344] Hudson G.M., Flannigan K.L., Erickson S.L., Vicentini F.A., Zamponi A., Hirota C.L., Alston L., Altier C., Ghosh S., Rioux K.P. (2017). Constitutive androstane receptor regulates the intestinal mucosal response to injury. Br. J. Pharmacol..

[345] Boussadia B., Gangarossa G., Mselli-Lakhal L., Rousset M.C., de Bock F., Lassere F., Ghosh C., Pascussi J.M., Janigro D., Marchi N. (2016). Lack of CAR impacts neuronal function and cerebrovascular integrity in vivo. Exp. Neurol..

[346] Litwa E., Rzemieniec J., Wnuk A., Lason W., Krzeptowski W., Kajta M. (2016). RXRα, PXR and CAR xenobiotic receptors mediate the apoptotic and neurotoxic actions of nonylphenol in mouse hippocampal cells. J. Steroid Biochem. Mol. Biol..

[347] Du Z.H., Xia J., Sun X.C., Li X.N., Zhang C., Zhao H.S., Zhu S.Y., Li J.L. (2017). A novel nuclear xenobiotic receptors (AhR/PXR/CAR)-mediated mechanism of DEHP-induced cerebellar toxicity in quails (Coturnix japonica) via disrupting CYP enzyme system homeostasis. Environ. Pollut..

[348] Chung S., Son G.H., Kim K. (2011). Circadian rhythm of adrenal glucocorticoid: Its regulation and clinical implications. Biochim. Biophys. Acta.

[349] Van Cauter E., Blackman J.D., Roland D., Spire J.P., Refetoff S., Polonsky K.S. (1991). Modulation of glucose regulation and insulin secretion by circadian rhythmicity and sleep. J. Clin. Investig..

[350] Czeisler C.A., Klerman E.B. (1999). Circadian and sleep-dependent regulation of hormone release in humans. Recent Prog. Horm. Res..

[351] Smith S.M., Vale W.W. (2006). The role of the hypothalamic-pituitary-adrenal axis in neuroendocrine responses to stress. Dialogues Clin. Neurosci..

[352] Thiboutot D., Jabara S., McAllister J.M., Sivarajah A., Gilliland K., Cong Z., Clawson G. (2003). Human skin is a steroidogenic tissue: Steroidogenic enzymes and cofactors are expressed in epidermis, normal sebocytes, and an immortalized sebocyte cell line (SEB-1). J. Investig. Dermatol..

[353] Lechner O., Wiegers G.J., Oliveira-Dos-Santos A.J., Dietrich H., Recheis H., Waterman M., Boyd R., Wick G. (2000). Glucocorticoid production in the murine thymus. Eur. J. Immunol..

[354] Pazirandeh A., Xue Y., Rafter I., Sjovall J., Jondal M., Okret S. (1999). Paracrine glucocorticoid activity produced by mouse thymic epithelial cells. FASEB J..

[355] Takeda Y., Miyamori I., Yoneda T., Iki K., Hatakeyama H., Blair I.A., Hsieh F.Y., Takeda R. (1994). Synthesis of corticosterone in the vascular wall. Endocrinology.

[356] Hostettler N., Bianchi P., Gennari-Moser C., Kassahn D., Schoonjans K., Corazza N., Brunner T. (2012). Local glucocorticoid production in the mouse lung is induced by immune cell stimulation. Allergy.

[357] Gomez-Sanchez C.E., Zhou M.Y., Cozza E.N., Morita H., Eddleman F.C., Gomez-Sanchez E.P. (1996). Corticosteroid synthesis in the central nervous system. Endocr. Res..

[358] Mueller M., Cima I., Noti M., Fuhrer A., Jakob S., Dubuquoy L., Schoonjans K., Brunner T. (2006). The nuclear receptor LRH-1 critically regulates extra-adrenal glucocorticoid synthesis in the intestine. J. Exp. Med..

[359] Cima I., Corazza N., Dick B., Fuhrer A., Herren S., Jakob S., Ayuni E., Mueller C., Brunner T. (2004). Intestinal epithelial cells synthesize glucocorticoids and regulate T cell activation. J. Exp. Med..

[360] Morris D.J., Ridlon J.M. (2017). Glucocorticoids and gut bacteria: “The GALF Hypothesis” in the metagenomic era. Steroids.

[361] Kuo T., McQueen A., Chen T.C., Wang J.C. (2015). Regulation of Glucose Homeostasis by Glucocorticoids. Adv. Exp. Med. Biol..

[362] Sapolsky R.M., Romero L.M., Munck A.U. (2000). How do glucocorticoids influence stress responses? Integrating permissive, suppressive, stimulatory, and preparative actions. Endocr. Rev..

[363] Barnes P.J. (1998). Anti-inflammatory actions of glucocorticoids: Molecular mechanisms. Clin. Sci..

[364] Vegiopoulos A., Herzig S. (2007). Glucocorticoids, metabolism and metabolic diseases. Mol. Cell. Endocrinol..

[365] McEwen B.S., De Kloet E.R., Rostene W. (1986). Adrenal steroid receptors and actions in the nervous system. Physiol. Rev..

[366] Spencer R.L., Young E.A., Choo P.H., McEwen B.S. (1990). Adrenal steroid type I and type II receptor binding: Estimates of in vivo receptor number, occupancy, and activation with varying level of steroid. Brain Res..

[367] Morimoto M., Morita N., Ozawa H., Yokoyama K., Kawata M. (1996). Distribution of glucocorticoid receptor immunoreactivity and mRNA in the rat brain: An immunohistochemical and in situ hybridization study. Neurosci. Res..

[368] Reul J.M., de Kloet E.R. (1985). Two receptor systems for corticosterone in rat brain: Microdistribution and differential occupation. Endocrinology.

[369] Encio I.J., Detera-Wadleigh S.D. (1991). The genomic structure of the human glucocorticoid receptor. J. Biol. Chem..

[370] Hollenberg S.M., Weinberger C., Ong E.S., Cerelli G., Oro A., Lebo R., Thompson E.B., Rosenfeld M.G., Evans R.M. (1985). Primary structure and expression of a functional human glucocorticoid receptor cDNA. Nature.

[371] Beger C., Gerdes K., Lauten M., Tissing W.J., Fernandez-Munoz I., Schrappe M., Welte K. (2003). Expression and structural analysis of glucocorticoid receptor isoform γ in human leukaemia cells using an isoform-specific real-time polymerase chain reaction approach. Br. J. Haematol..

[372] Lu N.Z., Cidlowski J.A. (2005). Translational regulatory mechanisms generate N-terminal glucocorticoid receptor isoforms with unique transcriptional target genes. Mol. Cell.

[373] Kumar R., Calhoun W.J. (2008). Differential regulation of the transcriptional activity of the glucocorticoid receptor through site-specific phosphorylation. Biologics.

[374] Ito K., Yamamura S., Essilfie-Quaye S., Cosio B., Ito M., Barnes P.J., Adcock I.M. (2006). Histone deacetylase 2-mediated deacetylation of the glucocorticoid receptor enables NF-κB suppression. J. Exp. Med..

[375] Duma D., Jewell C.M., Cidlowski J.A. (2006). Multiple glucocorticoid receptor isoforms and mechanisms of post-translational modification. J. Steroid Biochem. Mol. Biol..

[376] Grad I., Picard D. (2007). The glucocorticoid responses are shaped by molecular chaperones. Mol. Cell. Endocrinol..

[377] Pratt W.B., Toft D.O. (1997). Steroid receptor interactions with heat shock protein and immunophilin chaperones. Endocr. Rev..

[378] Beato M. (1989). Gene regulation by steroid hormones. Cell.

[379] Freedman L.P. (1992). Anatomy of the steroid receptor zinc finger region. Endocr. Rev..

[380] Revollo J.R., Cidlowski J.A. (2009). Mechanisms generating diversity in glucocorticoid receptor signaling. Ann. N. Y. Acad. Sci..

[381] Surjit M., Ganti K.P., Mukherji A., Ye T., Hua G., Metzger D., Li M., Chambon P. (2011). Widespread negative response elements mediate direct repression by agonist-liganded glucocorticoid receptor. Cell.

[382] Adcock I.M., Nasuhara Y., Stevens D.A., Barnes P.J. (1999). Ligand-induced differentiation of glucocorticoid receptor (GR) trans-repression and transactivation: Preferential targetting of NF-κB and lack of I-κB involvement. Br. J. Pharmacol..

[383] Scheinman R.I., Gualberto A., Jewell C.M., Cidlowski J.A., Baldwin A.S. (1995). Characterization of mechanisms involved in transrepression of NF-κ B by activated glucocorticoid receptors. Mol. Cell. Biol..

[384] Tan C.K., Wahli W. (2016). A trilogy of glucocorticoid receptor actions. Proc. Natl. Acad. Sci. USA.

[385] Van Rossum E.F., Lamberts S.W. (2004). Polymorphisms in the glucocorticoid receptor gene and their associations with metabolic parameters and body composition. Recent Prog. Horm. Res..

[386] Park E., Lee M.S., Baik S.M., Cho E.B., Son G.H., Seong J.Y., Lee K.H., Kim K. (2009). Nova-1 mediates glucocorticoid-induced inhibition of pre-mRNA splicing of gonadotropin-releasing hormone transcripts. J. Biol. Chem..

[387] Park O.H., Do E., Kim Y.K. (2015). A new function of glucocorticoid receptor: Regulation of mRNA stability. BMB Rep..

[388] Smith L.K., Shah R.R., Cidlowski J.A. (2010). Glucocorticoids modulate microRNA expression and processing during lymphocyte apoptosis. J. Biol. Chem..

[389] Savory J.G., Prefontaine G.G., Lamprecht C., Liao M., Walther R.F., Lefebvre Y.A., Hache R.J. (2001). Glucocorticoid receptor homodimers and glucocorticoid-mineralocorticoid receptor heterodimers form in the cytoplasm through alternative dimerization interfaces. Mol. Cell. Biol..

[390] Liu W., Wang J., Sauter N.K., Pearce D. (1995). Steroid receptor heterodimerization demonstrated in vitro and in vivo. Proc. Natl. Acad. Sci. USA.

[391] Croxtall J.D., Choudhury Q., Flower R.J. (2000). Glucocorticoids act within minutes to inhibit recruitment of signalling factors to activated EGF receptors through a receptor-dependent, transcription-independent mechanism. Br. J. Pharmacol..

[392] Solito E., Mulla A., Morris J.F., Christian H.C., Flower R.J., Buckingham J.C. (2003). Dexamethasone induces rapid serine-phosphorylation and membrane translocation of annexin 1 in a human folliculostellate cell line via a novel nongenomic mechanism involving the glucocorticoid receptor, protein kinase C, phosphatidylinositol 3-kinase, and mitogen-activated protein kinase. Endocrinology.

[393] Groeneweg F.L., Karst H., de Kloet E.R., Joels M. (2012). Mineralocorticoid and glucocorticoid receptors at the neuronal membrane, regulators of nongenomic corticosteroid signalling. Mol. Cell. Endocrinol..

[394] Kadmiel M., Cidlowski J.A. (2013). Glucocorticoid receptor signaling in health and disease. Trends Pharmacol. Sci..

[395] Whirledge S., DeFranco D.B. (2018). Glucocorticoid Signaling in Health and Disease: Insights from Tissue-Specific GR Knockout Mice. Endocrinology.

[396] Nader N., Chrousos G.P., Kino T. (2010). Interactions of the circadian CLOCK system and the HPA axis. Trends Endocrinol. Metab..

[397] Grimaldi B., Nakahata Y., Sahar S., Kaluzova M., Gauthier D., Pham K., Patel N., Hirayama J., Sassone-Corsi P. (2007). Chromatin remodeling and circadian control: Master regulator CLOCK is an enzyme. Cold Spring Harb. Symp. Quant. Biol..

[398] Nader N., Chrousos G.P., Kino T. (2009). Circadian rhythm transcription factor CLOCK regulates the transcriptional activity of the glucocorticoid receptor by acetylating its hinge region lysine cluster: Potential physiological implications. FASEB J..

[399] Balsalobre A., Brown S.A., Marcacci L., Tronche F., Kellendonk C., Reichardt H.M., Schutz G., Schibler U. (2000). Resetting of circadian time in peripheral tissues by glucocorticoid signaling. Science.

[400] Ramamoorthy S., Cidlowski J.A. (2013). Exploring the molecular mechanisms of glucocorticoid receptor action from sensitivity to resistance. Endocr. Dev..

[401] Wiley J.W., Higgins G.A., Athey B.D. (2016). Stress and glucocorticoid receptor transcriptional programming in time and space: Implications for the brain-gut axis. Neurogastroenterol. Motil..

[402] Hussain M.M., Pan X. (2009). Clock genes, intestinal transport and plasma lipid homeostasis. Trends Endocrinol. Metab..

[403] Hoogerwerf W.A., Hellmich H.L., Cornelissen G., Halberg F., Shahinian V.B., Bostwick J., Savidge T.C., Cassone V.M. (2007). Clock gene expression in the murine gastrointestinal tract: Endogenous rhythmicity and effects of a feeding regimen. Gastroenterology.

[404] Sladek M., Rybova M., Jindrakova Z., Zemanova Z., Polidarova L., Mrnka L., O’Neill J., Pacha J., Sumova A. (2007). Insight into the circadian clock within rat colonic epithelial cells. Gastroenterology.

[405] Pan X., Hussain M.M. (2009). Clock is important for food and circadian regulation of macronutrient absorption in mice. J. Lipid Res..

[406] Thaiss C.A., Zeevi D., Levy M., Zilberman-Schapira G., Suez J., Tengeler A.C., Abramson L., Katz M.N., Korem T., Zmora N. (2014). Transkingdom control of microbiota diurnal oscillations promotes metabolic homeostasis. Cell.

[407] Leone V., Gibbons S.M., Martinez K., Hutchison A.L., Huang E.Y., Cham C.M., Pierre J.F., Heneghan A.F., Nadimpalli A., Hubert N. (2015). Effects of diurnal variation of gut microbes and high-fat feeding on host circadian clock function and metabolism. Cell Host Microbe.

[408] Thaiss C.A., Levy M., Korem T., Dohnalova L., Shapiro H., Jaitin D.A., David E., Winter D.R., Gury-BenAri M., Tatirovsky E. (2016). Microbiota Diurnal Rhythmicity Programs Host Transcriptome Oscillations. Cell.

[409] Imai E., Stromstedt P.E., Quinn P.G., Carlstedt-Duke J., Gustafsson J.A., Granner D.K. (1990). Characterization of a complex glucocorticoid response unit in the phosphoenolpyruvate carboxykinase gene. Mol. Cell. Biol..

[410] Rauch A., Seitz S., Baschant U., Schilling A.F., Illing A., Stride B., Kirilov M., Mandic V., Takacz A., Schmidt-Ullrich R. (2010). Glucocorticoids suppress bone formation by attenuating osteoblast differentiation via the monomeric glucocorticoid receptor. Cell Metab..

[411] Zou W., Yang S., Zhang T., Sun H., Wang Y., Xue H., Zhou D. (2015). Hypoxia enhances glucocorticoid-induced apoptosis and cell cycle arrest via the PI3K/Akt signaling pathway in osteoblastic cells. J. Bone Miner. Metab..

[412] Kim H.J., Zhao H., Kitaura H., Bhattacharyya S., Brewer J.A., Muglia L.J., Ross F.P., Teitelbaum S.L. (2006). Glucocorticoids suppress bone formation via the osteoclast. J. Clin. Investig..

[413] Lee M.J., Pramyothin P., Karastergiou K., Fried S.K. (2014). Deconstructing the roles of glucocorticoids in adipose tissue biology and the development of central obesity. Biochim. Biophys. Acta.

[414] Chinenov Y., Gupte R., Rogatsky I. (2013). Nuclear receptors in inflammation control: Repression by GR and beyond. Mol. Cell. Endocrinol..

[415] Busillo J.M., Cidlowski J.A. (2013). The five Rs of glucocorticoid action during inflammation: Ready, reinforce, repress, resolve, and restore. Trends Endocrinol. Metab..

[416] Faubion W.A., Loftus E.V., Harmsen W.S., Zinsmeister A.R., Sandborn W.J. (2001). The natural history of corticosteroid therapy for inflammatory bowel disease: A population-based study. Gastroenterology.

[417] Howell M.P., Muglia L.J. (2006). Effects of genetically altered brain glucocorticoid receptor action on behavior and adrenal axis regulation in mice. Front. Neuroendocrinol..

[418] Kleiman A., Tuckermann J.P. (2007). Glucocorticoid receptor action in beneficial and side effects of steroid therapy: Lessons from conditional knockout mice. Mol. Cell. Endocrinol..

[419] Gallant C., Kenny P. (1986). Oral glucocorticoids and their complications. A review. J. Am. Acad. Dermatol..

[420] Heimdal K., Hirschberg H., Slettebo H., Watne K., Nome O. (1992). High incidence of serious side effects of high-dose dexamethasone treatment in patients with epidural spinal cord compression. J. Neurooncol..

[421] Dinan T.G., Cryan J., Shanahan F., Keeling P.W., Quigley E.M. (2010). IBS: An epigenetic perspective. Nat. Rev. Gastroenterol. Hepatol..

[422] Elsenbruch S. (2011). Abdominal pain in Irritable Bowel Syndrome: A review of putative psychological, neural and neuro-immune mechanisms. Brain Behav. Immun..

[423] Mayer E.A. (2000). The neurobiology of stress and gastrointestinal disease. Gut.

[424] Crofford L.J., Pillemer S.R., Kalogeras K.T., Cash J.M., Michelson D., Kling M.A., Sternberg E.M., Gold P.W., Chrousos G.P., Wilder R.L. (1994). Hypothalamic-pituitary-adrenal axis perturbations in patients with fibromyalgia. Arthritis Rheum..

[425] Johnson E.O., Vlachoyiannopoulos P.G., Skopouli F.N., Tzioufas A.G., Moutsopoulos H.M. (1998). Hypofunction of the stress axis in Sjogren’s syndrome. J. Rheumatol..

[426] Buske-Kirschbaum A., Jobst S., Wustmans A., Kirschbaum C., Rauh W., Hellhammer D. (1997). Attenuated free cortisol response to psychosocial stress in children with atopic dermatitis. Psychosom. Med..

[427] Nanthakumar N.N., Meng D., Newburg D.S. (2013). Glucocorticoids and microbiota regulate ontogeny of intestinal fucosyltransferase 2 requisite for gut homeostasis. Glycobiology.

[428] Reichardt S.D., Foller M., Rexhepaj R., Pathare G., Minnich K., Tuckermann J.P., Lang F., Reichardt H.M. (2012). Glucocorticoids enhance intestinal glucose uptake via the dimerized glucocorticoid receptor in enterocytes. Endocrinology.

[429] Thiesen A., Wild G.E., Tappenden K.A., Drozdowski L., Keelan M., Thomson B.K., McBurney M.I., Clandinin M.T., Thomson A.B. (2003). The locally acting glucocorticosteroid budesonide enhances intestinal sugar uptake following intestinal resection in rats. Gut.

[430] Boivin M.A., Ye D., Kennedy J.C., Al-Sadi R., Shepela C., Ma T.Y. (2007). Mechanism of glucocorticoid regulation of the intestinal tight junction barrier. Am. J. Physiol. Gastrointest. Liver Physiol..

[431] Lu L., Li T., Williams G., Petit E., Borowsky M., Walker W.A. (2011). Hydrocortisone induces changes in gene expression and differentiation in immature human enterocytes. Am. J. Physiol. Gastrointest. Liver Physiol..

[432] Filaretova L.P., Filaretov A.A., Makara G.B. (1998). Corticosterone increase inhibits stress-induced gastric erosions in rats. Am. J. Physiol..

[433] Vyas A., Mitra R., Shankaranarayana Rao B.S., Chattarji S. (2002). Chronic stress induces contrasting patterns of dendritic remodeling in hippocampal and amygdaloid neurons. J. Neurosci..

[434] Stewart M.G., Davies H.A., Sandi C., Kraev I.V., Rogachevsky V.V., Peddie C.J., Rodriguez J.J., Cordero M.I., Donohue H.S., Gabbott P.L. (2005). Stress suppresses and learning induces plasticity in CA3 of rat hippocampus: A three-dimensional ultrastructural study of thorny excrescences and their postsynaptic densities. Neuroscience.

[435] Watanabe Y., Gould E., McEwen B.S. (1992). Stress induces atrophy of apical dendrites of hippocampal CA3 pyramidal neurons. Brain Res..

[436] Samarasinghe R.A., Di Maio R., Volonte D., Galbiati F., Lewis M., Romero G., DeFranco D.B. (2011). Nongenomic glucocorticoid receptor action regulates gap junction intercellular communication and neural progenitor cell proliferation. Proc. Natl. Acad. Sci. USA.

[437] Popoli M., Yan Z., McEwen B.S., Sanacora G. (2011). The stressed synapse: The impact of stress and glucocorticoids on glutamate transmission. Nat. Rev. Neurosci..

[438] Arango-Lievano M., Lambert W.M., Bath K.G., Garabedian M.J., Chao M.V., Jeanneteau F. (2015). Neurotrophic-priming of glucocorticoid receptor signaling is essential for neuronal plasticity to stress and antidepressant treatment. Proc. Natl. Acad. Sci. USA.

[439] Blandino P., Barnum C.J., Deak T. (2006). The involvement of norepinephrine and microglia in hypothalamic and splenic IL-1β responses to stress. J. Neuroimmunol..

[440] Emmetsberger J., Tsirka S.E. (2012). Microglial inhibitory factor (MIF/TKP) mitigates secondary damage following spinal cord injury. Neurobiol. Dis..

[441] Wohleb E.S., Hanke M.L., Corona A.W., Powell N.D., Stiner L.M., Bailey M.T., Nelson R.J., Godbout J.P., Sheridan J.F. (2011). β-Adrenergic receptor antagonism prevents anxiety-like behavior and microglial reactivity induced by repeated social defeat. J. Neurosci..

[442] Alexander J.K., DeVries A.C., Kigerl K.A., Dahlman J.M., Popovich P.G. (2009). Stress exacerbates neuropathic pain via glucocorticoid and NMDA receptor activation. Brain Behav. Immun..

[443] Wang S., Lim G., Zeng Q., Sung B., Yang L., Mao J. (2005). Central glucocorticoid receptors modulate the expression and function of spinal NMDA receptors after peripheral nerve injury. J. Neurosci..

[444] Sandi C., Merino J.J., Cordero M.I., Touyarot K., Venero C. (2001). Effects of chronic stress on contextual fear conditioning and the hippocampal expression of the neural cell adhesion molecule, its polysialylation, and L1. Neuroscience.

[445] Dubovsky A.N., Arvikar S., Stern T.A., Axelrod L. (2012). The neuropsychiatric complications of glucocorticoid use: Steroid psychosis revisited. Psychosomatics.

[446] Weaver I.C., Cervoni N., Champagne F.A., D’Alessio A.C., Sharma S., Seckl J.R., Dymov S., Szyf M., Meaney M.J. (2004). Epigenetic programming by maternal behavior. Nat. Neurosci..

[447] Liu D., Diorio J., Tannenbaum B., Caldji C., Francis D., Freedman A., Sharma S., Pearson D., Plotsky P.M., Meaney M.J. (1997). Maternal care, hippocampal glucocorticoid receptors, and hypothalamic-pituitary-adrenal responses to stress. Science.

[448] Caldji C., Tannenbaum B., Sharma S., Francis D., Plotsky P.M., Meaney M.J. (1998). Maternal care during infancy regulates the development of neural systems mediating the expression of fearfulness in the rat. Proc. Natl. Acad. Sci. USA.

[449] Dinan T.G., Scott L.V. (2005). Anatomy of melancholia: Focus on hypothalamic-pituitary-adrenal axis overactivity and the role of vasopressin. J. Anat..

[450] de Kloet C.S., Vermetten E., Geuze E., Kavelaars A., Heijnen C.J., Westenberg H.G. (2006). Assessment of HPA-axis function in posttraumatic stress disorder: Pharmacological and non-pharmacological challenge tests, a review. J. Psychiatr. Res..

[451] Schechter D.S., Moser D.A., Paoloni-Giacobino A., Stenz L., Gex-Fabry M., Aue T., Adouan W., Cordero M.I., Suardi F., Manini A. (2015). Methylation of NR3C1 is related to maternal PTSD, parenting stress and maternal medial prefrontal cortical activity in response to child separation among mothers with histories of violence exposure. Front. Psychol..

[452] Webster M.J., Knable M.B., O’Grady J., Orthmann J., Weickert C.S. (2002). Regional specificity of brain glucocorticoid receptor mRNA alterations in subjects with schizophrenia and mood disorders. Mol. Psychiatry.

[453] Bailey M.T. (2014). Influence of stressor-induced nervous system activation on the intestinal microbiota and the importance for immunomodulation. Adv. Exp. Med. Biol..

[454] Bailey M.T., Dowd S.E., Galley J.D., Hufnagle A.R., Allen R.G., Lyte M. (2011). Exposure to a social stressor alters the structure of the intestinal microbiota: Implications for stressor-induced immunomodulation. Brain Behav. Immun..

[455] Bharwani A., Mian M.F., Foster J.A., Surette M.G., Bienenstock J., Forsythe P. (2016). Structural & functional consequences of chronic psychosocial stress on the microbiome & host. Psychoneuroendocrinology.

[456] De Palma G., Blennerhassett P., Lu J., Deng Y., Park A.J., Green W., Denou E., Silva M.A., Santacruz A., Sanz Y. (2015). Microbiota and host determinants of behavioural phenotype in maternally separated mice. Nat. Commun..

[457] De Palma G., Collins S.M., Bercik P., Verdu E.F. (2014). The microbiota-gut-brain axis in gastrointestinal disorders: Stressed bugs, stressed brain or both?. J. Physiol..

[458] Moloney R.D., Desbonnet L., Clarke G., Dinan T.G., Cryan J.F. (2014). The microbiome: Stress, health and disease. Mamm. Genome.

[459] Bridgewater L.C., Zhang C., Wu Y., Hu W., Zhang Q., Wang J., Li S., Zhao L. (2017). Gender-based differences in host behavior and gut microbiota composition in response to high fat diet and stress in a mouse model. Sci. Rep..

[460] Huang E.Y., Inoue T., Leone V.A., Dalal S., Touw K., Wang Y., Musch M.W., Theriault B., Higuchi K., Donovan S. (2015). Using corticosteroids to reshape the gut microbiome: Implications for inflammatory bowel diseases. Inflamm. Bowel Dis..

[461] Unsal H., Balkaya M., Unsal C., Biyik H., Basbulbul G., Poyrazoglu E. (2008). The short-term effects of different doses of dexamethasone on the numbers of some bacteria in the ileum. Dig. Dis. Sci..

[462] Forsythe P., Kunze W.A., Bienenstock J. (2012). On communication between gut microbes and the brain. Curr. Opin. Gastroenterol..

[463] Ait-Belgnaoui A., Durand H., Cartier C., Chaumaz G., Eutamene H., Ferrier L., Houdeau E., Fioramonti J., Bueno L., Theodorou V. (2012). Prevention of gut leakiness by a probiotic treatment leads to attenuated HPA response to an acute psychological stress in rats. Psychoneuroendocrinology.

[464] Burokas A., Arboleya S., Moloney R.D., Peterson V.L., Murphy K., Clarke G., Stanton C., Dinan T.G., Cryan J.F. (2017). Targeting the Microbiota-Gut-Brain Axis: Prebiotics Have Anxiolytic and Antidepressant-like Effects and Reverse the Impact of Chronic Stress in Mice. Biol. Psychiatry.

[465] Gareau M.G., Jury J., MacQueen G., Sherman P.M., Perdue M.H. (2007). Probiotic treatment of rat pups normalises corticosterone release and ameliorates colonic dysfunction induced by maternal separation. Gut.

[466] Hawkins U.A., Gomez-Sanchez E.P., Gomez-Sanchez C.M., Gomez-Sanchez C.E. (2012). The ubiquitous mineralocorticoid receptor: Clinical implications. Curr. Hypertens. Rep..

[467] Herman J.P., Patel P.D., Akil H., Watson S.J. (1989). Localization and regulation of glucocorticoid and mineralocorticoid receptor messenger RNAs in the hippocampal formation of the rat. Mol. Endocrinol..

[468] Vallee S.M., Grillo C.A., Gonzalez S., Cosen-Binker L., de Kloet E.R., McEwen B.S., De Nicola A.F. (1995). Further studies in deoxycorticosterone acetate treated rats: Brain content of mineralocorticoid and glucocorticoid receptors and effect of steroid antagonists on salt intake. Neuroendocrinology.

[469] Turner B.B. (1990). Sex difference in glucocorticoid binding in rat pituitary is estrogen dependent. Life Sci..

[470] Castren M., Patchev V.K., Almeida O.F., Holsboer F., Trapp T., Castren E. (1995). Regulation of rat mineralocorticoid receptor expression in neurons by progesterone. Endocrinology.

[471] Zennaro M.C., Le Menuet D., Lombes M. (1996). Characterization of the human mineralocorticoid receptor gene 5′-regulatory region: Evidence for differential hormonal regulation of two alternative promoters via nonclassical mechanisms. Mol. Endocrinol..

[472] Wang J., Li X., Ke Y., Lu Y., Wang F., Fan N., Sun H., Zhang H., Liu R., Yang J. (2012). GPR48 increases mineralocorticoid receptor gene expression. J. Am. Soc. Nephrol..

[473] Kwak S.P., Patel P.D., Thompson R.C., Akil H., Watson S.J. (1993). 5′-Heterogeneity of the mineralocorticoid receptor messenger ribonucleic acid: Differential expression and regulation of splice variants within the rat hippocampus. Endocrinology.

[474] Zennaro M.C., Keightley M.C., Kotelevtsev Y., Conway G.S., Soubrier F., Fuller P.J. (1995). Human mineralocorticoid receptor genomic structure and identification of expressed isoforms. J. Biol. Chem..

[475] Hesen W., Karst H., Meijer O., Cole T.J., Schmid W., de Kloet E.R., Schutz G., Joels M. (1996). Hippocampal cell responses in mice with a targeted glucocorticoid receptor gene disruption. J. Neurosci..

[476] Karst H., Wadman W.J., Joels M. (1994). Corticosteroid receptor-dependent modulation of calcium currents in rat hippocampal CA1 neurons. Brain Res..

[477] Kerr D.S., Campbell L.W., Thibault O., Landfield P.W. (1992). Hippocampal glucocorticoid receptor activation enhances voltage-dependent Ca^2+^ conductances: Relevance to brain aging. Proc. Natl. Acad. Sci. USA.

[478] Caprio M., Feve B., Claes A., Viengchareun S., Lombes M., Zennaro M.C. (2007). Pivotal role of the mineralocorticoid receptor in corticosteroid-induced adipogenesis. FASEB J..

[479] Marzolla V., Armani A., Zennaro M.C., Cinti F., Mammi C., Fabbri A., Rosano G.M., Caprio M. (2012). The role of the mineralocorticoid receptor in adipocyte biology and fat metabolism. Mol. Cell. Endocrinol..

[480] Gomez-Sanchez E.P., Gomez-Sanchez C.E. (2012). Central regulation of blood pressure by the mineralocorticoid receptor. Mol. Cell. Endocrinol..

[481] Harris A.P., Holmes M.C., de Kloet E.R., Chapman K.E., Seckl J.R. (2013). Mineralocorticoid and glucocorticoid receptor balance in control of HPA axis and behaviour. Psychoneuroendocrinology.

[482] De Kloet E.R., Vreugdenhil E., Oitzl M.S., Joels M. (1998). Brain corticosteroid receptor balance in health and disease. Endocr. Rev..

[483] Ou X.M., Storring J.M., Kushwaha N., Albert P.R. (2001). Heterodimerization of mineralocorticoid and glucocorticoid receptors at a novel negative response element of the 5-HT1A receptor gene. J. Biol. Chem..

[484] Arriza J.L., Weinberger C., Cerelli G., Glaser T.M., Handelin B.L., Housman D.E., Evans R.M. (1987). Cloning of human mineralocorticoid receptor complementary DNA: Structural and functional kinship with the glucocorticoid receptor. Science.

[485] Ackermann D., Gresko N., Carrel M., Loffing-Cueni D., Habermehl D., Gomez-Sanchez C., Rossier B.C., Loffing J. (2010). In vivo nuclear translocation of mineralocorticoid and glucocorticoid receptors in rat kidney: Differential effect of corticosteroids along the distal tubule. Am. J. Physiol. Renal Physiol..

[486] Fuller P.J., Brennan F.E., Burgess J.S. (2000). Acute differential regulation by corticosteroids of epithelial sodium channel subunit and Nedd4 mRNA levels in the distal colon. Pflugers Arch..

[487] Lastra-Gonzalez G., Manrique-Acevedo C., Sowers J.R. (2008). The role of aldosterone in cardiovascular disease in people with diabetes and hypertension: An update. Curr. Diabetes Rep..

[488] Zennaro M.C., Caprio M., Feve B. (2009). Mineralocorticoid receptors in the metabolic syndrome. Trends Endocrinol. Metab..

[489] Vogt B., Burnier M. (2009). Aldosterone and cardiovascular risk. Curr. Hypertens. Rep..

[490] Tirosh A., Garg R., Adler G.K. (2010). Mineralocorticoid receptor antagonists and the metabolic syndrome. Curr. Hypertens. Rep..

[491] Cheskis B.J. (2004). Regulation of cell signalling cascades by steroid hormones. J. Cell Biochem..

[492] Grossmann C., Gekle M. (2009). New aspects of rapid aldosterone signaling. Mol. Cell. Endocrinol..

[493] Grossmann C., Gekle M. (2007). Non-classical actions of the mineralocorticoid receptor: Misuse of EGF receptors?. Mol. Cell. Endocrinol..

[494] Funder J.W. (2001). Non-genomic actions of aldosterone: Role in hypertension. Curr. Opin Nephrol. Hypertens..

[495] Huang S., Zhang A., Ding G., Chen R. (2009). Aldosterone-induced mesangial cell proliferation is mediated by EGF receptor transactivation. Am. J. Physiol. Renal Physiol..

[496] Krug A.W., Schuster C., Gassner B., Freudinger R., Mildenberger S., Troppmair J., Gekle M. (2002). Human epidermal growth factor receptor-1 expression renders Chinese hamster ovary cells sensitive to alternative aldosterone signaling. J. Biol. Chem..

[497] Michea L., Delpiano A.M., Hitschfeld C., Lobos L., Lavandero S., Marusic E.T. (2005). Eplerenone blocks nongenomic effects of aldosterone on the Na^+^/H^+^ exchanger, intracellular Ca^2+^ levels, and vasoconstriction in mesenteric resistance vessels. Endocrinology.

[498] Krozowski Z.S., Funder J.W. (1983). Renal mineralocorticoid receptors and hippocampal corticosterone-binding species have identical intrinsic steroid specificity. Proc. Natl. Acad. Sci. USA.

[499] Veldhuis H.D., Van Koppen C., Van Ittersum M., De Kloet E.R. (1982). Specificity of the adrenal steroid receptor system in rat hippocampus. Endocrinology.

[500] De Kloet E.R., Reul J.M. (1987). Feedback action and tonic influence of corticosteroids on brain function: A concept arising from the heterogeneity of brain receptor systems. Psychoneuroendocrinology.

[501] Karst H., Berger S., Turiault M., Tronche F., Schutz G., Joels M. (2005). Mineralocorticoid receptors are indispensable for nongenomic modulation of hippocampal glutamate transmission by corticosterone. Proc. Natl. Acad. Sci. USA.

[502] Seckl J.R. (2004). 11β-hydroxysteroid dehydrogenases: Changing glucocorticoid action. Curr. Opin. Pharmacol..

[503] Yang H., Dou W., Lou J., Leng Y., Shen J. (2008). Discovery of novel inhibitors of 11β-hydroxysteroid dehydrogenase type 1 by docking and pharmacophore modeling. Bioorg. Med. Chem. Lett..

[504] Zhang Z.H., Kang Y.M., Yu Y., Wei S.G., Schmidt T.J., Johnson A.K., Felder R.B. (2006). 11β-hydroxysteroid dehydrogenase type 2 activity in hypothalamic paraventricular nucleus modulates sympathetic excitation. Hypertension.

[505] Chen J., Gomez-Sanchez C.E., Penman A., May P.J., Gomez-Sanchez E. (2014). Expression of mineralocorticoid and glucocorticoid receptors in preautonomic neurons of the rat paraventricular nucleus. Am. J. Physiol. Regul. Integr. Comp. Physiol..

[506] Geerling J.C., Loewy A.D. (2006). Aldosterone-sensitive neurons in the nucleus of the solitary tract: Bidirectional connections with the central nucleus of the amygdala. J. Comp. Neurol..

[507] Chantong B., Kratschmar D.V., Nashev L.G., Balazs Z., Odermatt A. (2012). Mineralocorticoid and glucocorticoid receptors differentially regulate NF-κB activity and pro-inflammatory cytokine production in murine BV-2 microglial cells. J. Neuroinflamm..

[508] Vazquez D.M., Lopez J.F., Morano M.I., Kwak S.P., Watson S.J., Akil H. (1998). Alpha, β, and γ mineralocorticoid receptor messenger ribonucleic acid splice variants: Differential expression and rapid regulation in the developing hippocampus. Endocrinology.

[509] Munier M., Meduri G., Viengchareun S., Leclerc P., Le Menuet D., Lombes M. (2010). Regulation of mineralocorticoid receptor expression during neuronal differentiation of murine embryonic stem cells. Endocrinology.

[510] Gass P., Kretz O., Wolfer D.P., Berger S., Tronche F., Reichardt H.M., Kellendonk C., Lipp H.P., Schmid W., Schutz G. (2000). Genetic disruption of mineralocorticoid receptor leads to impaired neurogenesis and granule cell degeneration in the hippocampus of adult mice. EMBO Rep..

[511] Brinks V., van der Mark M.H., de Kloet E.R., Oitzl M.S. (2007). Differential MR/GR activation in mice results in emotional states beneficial or impairing for cognition. Neural Plast..

[512] Sterlemann V., Ganea K., Liebl C., Harbich D., Alam S., Holsboer F., Muller M.B., Schmidt M.V. (2008). Long-term behavioral and neuroendocrine alterations following chronic social stress in mice: Implications for stress-related disorders. Horm. Behav..

[513] Qi X.R., Kamphuis W., Wang S., Wang Q., Lucassen P.J., Zhou J.N., Swaab D.F. (2013). Aberrant stress hormone receptor balance in the human prefrontal cortex and hypothalamic paraventricular nucleus of depressed patients. Psychoneuroendocrinology.

[514] Wu T.C., Chen H.T., Chang H.Y., Yang C.Y., Hsiao M.C., Cheng M.L., Chen J.C. (2013). Mineralocorticoid receptor antagonist spironolactone prevents chronic corticosterone induced depression-like behavior. Psychoneuroendocrinology.

[515] Okuhara D.Y., Beck S.G. (1998). Corticosteroids alter 5-hydroxytryptamine1A receptor-effector pathway in hippocampal subfield CA3 pyramidal cells. J. Pharmacol. Exp. Ther..

[516] Beck S.G., Choi K.C., List T.J., Okuhara D.Y., Birnsteil S. (1996). Corticosterone alters 5-HT1A receptor-mediated hyperpolarization in area CA1 hippocampal pyramidal neurons. Neuropsychopharmacology.

[517] Anacker C., Zunszain P.A., Carvalho L.A., Pariante C.M. (2011). The glucocorticoid receptor: Pivot of depression and of antidepressant treatment?. Psychoneuroendocrinology.

[518] Maletic V., Robinson M., Oakes T., Iyengar S., Ball S.G., Russell J. (2007). Neurobiology of depression: An integrated view of key findings. Int. J. Clin. Pract..

[519] Yagi S., Akaike M., Aihara K., Iwase T., Yoshida S., Sumitomo-Ueda Y., Ikeda Y., Ishikawa K., Matsumoto T., Sata M. (2011). High plasma aldosterone concentration is a novel risk factor of cognitive impairment in patients with hypertension. Hypertens. Res..

[520] Yau J.L., Hibberd C., Noble J., Seckl J.R. (2002). The effect of chronic fluoxetine treatment on brain corticosteroid receptor mRNA expression and spatial memory in young and aged rats. Brain Res. Mol. Brain Res..

[521] Tytherleigh M.Y., Vedhara K., Lightman S.L. (2004). Mineralocorticoid and glucocorticoid receptors and their differential effects on memory performance in people with Addison’s disease. Psychoneuroendocrinology.

[522] Yau J.L., Olsson T., Morris R.G., Meaney M.J., Seckl J.R. (1995). Glucocorticoids, hippocampal corticosteroid receptor gene expression and antidepressant treatment: Relationship with spatial learning in young and aged rats. Neuroscience.

[523] Yau J.L., Noble J., Hibberd C., Rowe W.B., Meaney M.J., Morris R.G., Seckl J.R. (2002). Chronic treatment with the antidepressant amitriptyline prevents impairments in water maze learning in aging rats. J. Neurosci..

[524] Cornelisse S., Joels M., Smeets T. (2011). A randomized trial on mineralocorticoid receptor blockade in men: Effects on stress responses, selective attention, and memory. Neuropsychopharmacology.

[525] Gruber C.J., Tschugguel W., Schneeberger C., Huber J.C. (2002). Production and actions of estrogens. N. Engl. J. Med..

[526] Zhu B.T., Han G.Z., Shim J.Y., Wen Y., Jiang X.R. (2006). Quantitative structure-activity relationship of various endogenous estrogen metabolites for human estrogen receptor α and β subtypes: Insights into the structural determinants favoring a differential subtype binding. Endocrinology.

[527] Riant E., Waget A., Cogo H., Arnal J.F., Burcelin R., Gourdy P. (2009). Estrogens protect against high-fat diet-induced insulin resistance and glucose intolerance in mice. Endocrinology.

[528] Carr M.C. (2003). The emergence of the metabolic syndrome with menopause. J. Clin. Endocrinol. Metab..

[529] Gao Q., Mezei G., Nie Y., Rao Y., Choi C.S., Bechmann I., Leranth C., Toran-Allerand D., Priest C.A., Roberts J.L. (2007). Anorectic estrogen mimics leptin’s effect on the rewiring of melanocortin cells and Stat3 signaling in obese animals. Nat. Med..

[530] Wren B.G. (2009). The benefits of oestrogen following menopause: Why hormone replacement therapy should be offered to postmenopausal women. Med. J. Aust..

[531] Flores R., Shi J., Fuhrman B., Xu X., Veenstra T.D., Gail M.H., Gajer P., Ravel J., Goedert J.J. (2012). Fecal microbial determinants of fecal and systemic estrogens and estrogen metabolites: A cross-sectional study. J. Transl. Med..

[532] Fuhrman B.J., Feigelson H.S., Flores R., Gail M.H., Xu X., Ravel J., Goedert J.J. (2014). Associations of the fecal microbiome with urinary estrogens and estrogen metabolites in postmenopausal women. J. Clin. Endocrinol. Metab..

[533] Moreno-Indias I., Sanchez-Alcoholado L., Sanchez-Garrido M.A., Martin-Nunez G.M., Perez-Jimenez F., Tena-Sempere M., Tinahones F.J., Queipo-Ortuno M.I. (2016). Neonatal Androgen Exposure Causes Persistent Gut Microbiota Dysbiosis Related to Metabolic Disease in Adult Female Rats. Endocrinology.

[534] Mueller S., Saunier K., Hanisch C., Norin E., Alm L., Midtvedt T., Cresci A., Silvi S., Orpianesi C., Verdenelli M.C. (2006). Differences in fecal microbiota in different European study populations in relation to age, gender, and country: A cross-sectional study. Appl. Environ. Microbiol..

[535] Dominianni C., Sinha R., Goedert J.J., Pei Z., Yang L., Hayes R.B., Ahn J. (2015). Sex, body mass index, and dietary fiber intake influence the human gut microbiome. PLoS ONE.

[536] Yurkovetskiy L., Burrows M., Khan A.A., Graham L., Volchkov P., Becker L., Antonopoulos D., Umesaki Y., Chervonsky A.V. (2013). Gender bias in autoimmunity is influenced by microbiota. Immunity.

[537] Plottel C.S., Blaser M.J. (2011). Microbiome and malignancy. Cell Host Microbe.

[538] Kisiela M., Skarka A., Ebert B., Maser E. (2012). Hydroxysteroid dehydrogenases (HSDs) in bacteria: A bioinformatic perspective. J. Steroid Biochem. Mol. Biol..

[539] Raftogianis R., Creveling C., Weinshilboum R., Weisz J. (2000). Estrogen metabolism by conjugation. J. Natl. Cancer Inst. Monogr..

[540] McBain A.J., Macfarlane G.T. (1998). Ecological and physiological studies on large intestinal bacteria in relation to production of hydrolytic and reductive enzymes involved in formation of genotoxic metabolites. J. Med. Microbiol..

[541] Kwa M., Plottel C.S., Blaser M.J., Adams S. (2016). The Intestinal Microbiome and Estrogen Receptor-Positive Female Breast Cancer. J. Natl. Cancer Inst..

[542] Reddy B.S., Weisburger J.H., Wynder E.L. (1974). Fecal bacterial β-glucuronidase: Control by diet. Science.

[543] Cenci G., Caldini G., Mastrandrea V., Votoni M.R. (1993). Carbohydrate enriched diets and bacterial glycosidases in rat faeces. Microbios.

[544] Eriyamremu G.E., Osagie V.E., Alufa O.I., Osaghae M.O., Oyibu F.A. (1995). Early biochemical events in mice exposed to cycas and fed a Nigerian-like diet. Ann. Nutr. Metab..

[545] Goldin B.R., Gorbach S.L. (1984). Alterations of the intestinal microflora by diet, oral antibiotics, and Lactobacillus: Decreased production of free amines from aromatic nitro compounds, azo dyes, and glucuronides. J. Natl. Cancer Inst..

[546] Martin F., Peltonen J., Laatikainen T., Pulkkinen M., Adlercreutz H. (1975). Excretion of progesterone metabolites and estriol in faeces from pregnant women during ampicillin administration. J. Steroid Biochem..

[547] Adlercreutz H., Martin F., Tikkanen M.J., Pulkkinen M. (1975). Effect of ampicillin administration on the excretion of twelve oestrogens in pregnancy urine. Acta Endocrinol..

[548] Hamalainen E., Korpela J.T., Adlercreutz H. (1987). Effect of oxytetracycline administration on intestinal metabolism of oestrogens and on plasma sex hormones in healthy men. Gut.

[549] Flint H.J., Duncan S.H., Scott K.P., Louis P. (2007). Interactions and competition within the microbial community of the human colon: Links between diet and health. Environ. Microbiol..

[550] Dabek M., McCrae S.I., Stevens V.J., Duncan S.H., Louis P. (2008). Distribution of β-glucosidase and β-glucuronidase activity and of β-glucuronidase gene gus in human colonic bacteria. FEMS Microbiol. Ecol..

[551] Nakamura J., Kubota Y., Miyaoka M., Saitoh T., Mizuno F., Benno Y. (2002). Comparison of four microbial enzymes in Clostridia and Bacteroides isolated from human feces. Microbiol. Immunol..

[552] Van Eldere J.R., De Pauw G., Eyssen H.J. (1987). Steroid sulfatase activity in a Peptococcus niger strain from the human intestinal microflora. Appl. Environ. Microbiol..

[553] Frankenfeld C.L., Atkinson C., Wahala K., Lampe J.W. (2014). Obesity prevalence in relation to gut microbial environments capable of producing equol or O-desmethylangolensin from the isoflavone daidzein. Eur. J. Clin. Nutr..

[554] Nakatsu C.H., Armstrong A., Clavijo A.P., Martin B.R., Barnes S., Weaver C.M. (2014). Fecal bacterial community changes associated with isoflavone metabolites in postmenopausal women after soy bar consumption. PLoS ONE.

[555] Usui T., Tochiya M., Sasaki Y., Muranaka K., Yamakage H., Himeno A., Shimatsu A., Inaguma A., Ueno T., Uchiyama S. (2013). Effects of natural S-equol supplements on overweight or obesity and metabolic syndrome in the Japanese, based on sex and equol status. Clin. Endocrinol..

[556] Velicer C.M., Heckbert S.R., Lampe J.W., Potter J.D., Robertson C.A., Taplin S.H. (2004). Antibiotic use in relation to the risk of breast cancer. JAMA.

[557] Boursi B., Mamtani R., Haynes K., Yang Y.X. (2015). Recurrent antibiotic exposure may promote cancer formation--Another step in understanding the role of the human microbiota?. Eur. J. Cancer.

[558] Shimizu K., Muranaka Y., Fujimura R., Ishida H., Tazume S., Shimamura T. (1998). Normalization of reproductive function in germfree mice following bacterial contamination. Exp. Anim..

[559] Green S., Walter P., Kumar V., Krust A., Bornert J.M., Argos P., Chambon P. (1986). Human oestrogen receptor cDNA: Sequence, expression and homology to v-erb-A. Nature.

[560] Kuiper G.G., Enmark E., Pelto-Huikko M., Nilsson S., Gustafsson J.A. (1996). Cloning of a novel receptor expressed in rat prostate and ovary. Proc. Natl. Acad. Sci. USA.

[561] Kuiper G.G., Lemmen J.G., Carlsson B., Corton J.C., Safe S.H., van der Saag P.T., van der Burg B., Gustafsson J.A. (1998). Interaction of estrogenic chemicals and phytoestrogens with estrogen receptor β. Endocrinology.

[562] Enmark E., Pelto-Huikko M., Grandien K., Lagercrantz S., Lagercrantz J., Fried G., Nordenskjold M., Gustafsson J.A. (1997). Human estrogen receptor β-gene structure, chromosomal localization, and expression pattern. J. Clin. Endocrinol. Metab..

[563] Sauerwein H., Pfaffl M., Hagen-Mann K., Malucelli A., Meyer H.H. (1995). Expression of estrogen and androgen receptor in the bovine gastrointestinal tract. Dtsch. Tierarztl. Wochenschr..

[564] Pfaffl M.W., Lange I.G., Meyer H.H. (2003). The gastrointestinal tract as target of steroid hormone action: Quantification of steroid receptor mRNA expression (AR, ERα, ERβ and PR) in 10 bovine gastrointestinal tract compartments by kinetic RT-PCR. J. Steroid Biochem. Mol. Biol..

[565] Singh S., Sheppard M.C., Langman M.J. (1993). Sex differences in the incidence of colorectal cancer: An exploration of oestrogen and progesterone receptors. Gut.

[566] Foley E.F., Jazaeri A.A., Shupnik M.A., Jazaeri O., Rice L.W. (2000). Selective loss of estrogen receptor β in malignant human colon. Cancer Res..

[567] Hess R.A., Gist D.H., Bunick D., Lubahn D.B., Farrell A., Bahr J., Cooke P.S., Greene G.L. (1997). Estrogen receptor (α and β) expression in the excurrent ducts of the adult male rat reproductive tract. J. Androl..

[568] Hileman S.M., Handa R.J., Jackson G.L. (1999). Distribution of estrogen receptor-β messenger ribonucleic acid in the male sheep hypothalamus. Biol. Reprod..

[569] Mitra S.W., Hoskin E., Yudkovitz J., Pear L., Wilkinson H.A., Hayashi S., Pfaff D.W., Ogawa S., Rohrer S.P., Schaeffer J.M. (2003). Immunolocalization of estrogen receptor β in the mouse brain: Comparison with estrogen receptor α. Endocrinology.

[570] Laflamme N., Nappi R.E., Drolet G., Labrie C., Rivest S. (1998). Expression and neuropeptidergic characterization of estrogen receptors (ERα and ERβ) throughout the rat brain: Anatomical evidence of distinct roles of each subtype. J. Neurobiol..

[571] Shughrue P., Scrimo P., Lane M., Askew R., Merchenthaler I. (1997). The distribution of estrogen receptor-β mRNA in forebrain regions of the estrogen receptor-α knockout mouse. Endocrinology.

[572] Shughrue P.J., Komm B., Merchenthaler I. (1996). The distribution of estrogen receptor-β mRNA in the rat hypothalamus. Steroids.

[573] Wang J.M., Liu L., Brinton R.D. (2008). Estradiol-17β-induced human neural progenitor cell proliferation is mediated by an estrogen receptor β-phosphorylated extracellularly regulated kinase pathway. Endocrinology.

[574] Li H., Ding C., Ding Z.L., Ling M., Wang T., Wang W., Huang B. (2017). 17β-Oestradiol promotes differentiation of human embryonic stem cells into dopamine neurons via cross-talk between insulin-like growth factors-1 and oestrogen receptor β. J. Cell. Mol. Med..

[575] Ogawa S., Eng V., Taylor J., Lubahn D.B., Korach K.S., Pfaff D.W. (1998). Roles of estrogen receptor-α gene expression in reproduction-related behaviors in female mice. Endocrinology.

[576] Hewitt S.C., Korach K.S. (2003). Oestrogen receptor knockout mice: Roles for oestrogen receptors α and β in reproductive tissues. Reproduction.

[577] Herbison A.E., Pape J.R. (2001). New evidence for estrogen receptors in gonadotropin-releasing hormone neurons. Front. Neuroendocrinol..

[578] Dorling A.A., Todman M.G., Korach K.S., Herbison A.E. (2003). Critical role for estrogen receptor α in negative feedback regulation of gonadotropin-releasing hormone mRNA expression in the female mouse. Neuroendocrinology.

[579] Heine P.A., Taylor J.A., Iwamoto G.A., Lubahn D.B., Cooke P.S. (2000). Increased adipose tissue in male and female estrogen receptor-α knockout mice. Proc. Natl. Acad. Sci. USA.

[580] Okura T., Koda M., Ando F., Niino N., Ohta S., Shimokata H. (2003). Association of polymorphisms in the estrogen receptor α gene with body fat distribution. Int. J. Obes. Relat. Metab. Disord..

[581] Thomas M.L., Ibarra M.J. (1987). Effects of ovariectomy on duodenal calcium transport in the rat: Altered ability to adapt to low-calcium diet. Proc. Soc. Exp. Biol. Med..

[582] Hope W.G., Bruns M.E., Thomas M.L. (1992). Regulation of duodenal insulin-like growth factor I and active calcium transport by ovariectomy in female rats. Proc Soc. Exp. Biol. Med..

[583] Xu Y., Nedungadi T.P., Zhu L., Sobhani N., Irani B.G., Davis K.E., Zhang X., Zou F., Gent L.M., Hahner L.D. (2011). Distinct hypothalamic neurons mediate estrogenic effects on energy homeostasis and reproduction. Cell Metab..

[584] Krezel W., Dupont S., Krust A., Chambon P., Chapman P.F. (2001). Increased anxiety and synaptic plasticity in estrogen receptor β -deficient mice. Proc. Natl. Acad. Sci. USA.

[585] Imwalle D.B., Gustafsson J.A., Rissman E.F. (2005). Lack of functional estrogen receptor β influences anxiety behavior and serotonin content in female mice. Physiol. Behav..

[586] Donner N., Handa R.J. (2009). Estrogen receptor β regulates the expression of tryptophan-hydroxylase 2 mRNA within serotonergic neurons of the rat dorsal raphe nuclei. Neuroscience.

[587] Rocha B.A., Fleischer R., Schaeffer J.M., Rohrer S.P., Hickey G.J. (2005). 17 Beta-estradiol-induced antidepressant-like effect in the forced swim test is absent in estrogen receptor-β knockout (BERKO) mice. Psychopharmacology.

[588] Walf A.A., Rhodes M.E., Frye C.A. (2004). Antidepressant effects of ERβ-selective estrogen receptor modulators in the forced swim test. Pharmacol. Biochem. Behav..

[589] Walf A.A., Frye C.A. (2005). ERβ-selective estrogen receptor modulators produce antianxiety behavior when administered systemically to ovariectomized rats. Neuropsychopharmacology.

[590] Lund T.D., Rovis T., Chung W.C., Handa R.J. (2005). Novel actions of estrogen receptor-β on anxiety-related behaviors. Endocrinology.

[591] Burgess L.H., Handa R.J. (1992). Chronic estrogen-induced alterations in adrenocorticotropin and corticosterone secretion, and glucocorticoid receptor-mediated functions in female rats. Endocrinology.

[592] Shapiro R.A., Xu C., Dorsa D.M. (2000). Differential transcriptional regulation of rat vasopressin gene expression by estrogen receptor α and β. Endocrinology.

[593] Miller W.J., Suzuki S., Miller L.K., Handa R., Uht R.M. (2004). Estrogen receptor (ER)β isoforms rather than ERα regulate corticotropin-releasing hormone promoter activity through an alternate pathway. J. Neurosci..

[594] Siiteri P.K., Murai J.T., Hammond G.L., Nisker J.A., Raymoure W.J., Kuhn R.W. (1982). The serum transport of steroid hormones. Recent Prog. Horm. Res..

[595] Barrett Mueller K., Lu Q., Mohammad N.N., Luu V., McCurley A., Williams G.H., Adler G.K., Karas R.H., Jaffe I.Z. (2014). Estrogen receptor inhibits mineralocorticoid receptor transcriptional regulatory function. Endocrinology.

[596] Quinkler M., Meyer B., Bumke-Vogt C., Grossmann C., Gruber U., Oelkers W., Diederich S., Bahr V. (2002). Agonistic and antagonistic properties of progesterone metabolites at the human mineralocorticoid receptor. Eur. J. Endocrinol..

[597] Watzka M., Beyenburg S., Blumcke I., Elger C.E., Bidlingmaier F., Stoffel-Wagner B. (2000). Expression of mineralocorticoid and glucocorticoid receptor mRNA in the human hippocampus. Neurosci. Lett..

[598] Kudielka B.M., Kirschbaum C. (2005). Sex differences in HPA axis responses to stress: A review. Biol. Psychol..

[599] Llorente R., Miguel-Blanco C., Aisa B., Lachize S., Borcel E., Meijer O.C., Ramirez M.J., De Kloet E.R., Viveros M.P. (2011). Long term sex-dependent psychoneuroendocrine effects of maternal deprivation and juvenile unpredictable stress in rats. J. Neuroendocrinol..

[600] Karandrea D., Kittas C., Kitraki E. (2000). Contribution of sex and cellular context in the regulation of brain corticosteroid receptors following restraInt. stress. Neuroendocrinology.

[601] Kitraki E., Kremmyda O., Youlatos D., Alexis M.N., Kittas C. (2004). Gender-dependent alterations in corticosteroid receptor status and spatial performance following 21 days of restraint stress. Neuroscience.

[602] Cyranowski J.M., Frank E., Young E., Shear M.K. (2000). Adolescent onset of the gender difference in lifetime rates of major depression: A theoretical model. Arch. Gen. Psychiatry.

[603] Piccinelli M., Wilkinson G. (2000). Gender differences in depression: Critical review. Br. J. Psychiatry.

[604] Suzuki S., Handa R.J. (2004). Regulation of estrogen receptor-β expression in the female rat hypothalamus: Differential effects of dexamethasone and estradiol. Endocrinology.

[605] Isgor C., Cecchi M., Kabbaj M., Akil H., Watson S.J. (2003). Estrogen receptor β in the paraventricular nucleus of hypothalamus regulates the neuroendocrine response to stress and is regulated by corticosterone. Neuroscience.

[606] Leret M.L., Molina-Holgado F., Gonzalez M.I. (1994). The effect of perinatal exposure to estrogens on the sexually dimorphic response to novelty. Physiol. Behav..

[607] Palermo-Neto J., Dorce V.A. (1990). Influences of estrogen and/or progesterone on some dopamine related behavior in rats. Gen. Pharmacol..

[608] Smanik P.A., Liu Q., Furminger T.L., Ryu K., Xing S., Mazzaferri E.L., Jhiang S.M. (1996). Cloning of the human sodium lodide symporter. Biochem. Biophys. Res. Commun..

[609] Dai G., Levy O., Carrasco N. (1996). Cloning and characterization of the thyroid iodide transporter. Nature.

[610] Yen P.M., Ando S., Feng X., Liu Y., Maruvada P., Xia X. (2006). Thyroid hormone action at the cellular, genomic and target gene levels. Mol. Cell. Endocrinol..

[611] Brent G.A. (2012). Mechanisms of thyroid hormone action. J. Clin. Investig..

[612] Braverman L.E., Ingbar S.H., Sterling K. (1970). Conversion of thyroxine (T_4_) to triiodothyronine (T_3_) in athyreotic human subjects. J. Clin. Investig..

[613] Kohrle J. (2000). The selenoenzyme family of deiodinase isozymes controls local thyroid hormone availability. Rev. Endocr. Metab. Disord..

[614] Sabatino L., Iervasi G., Ferrazzi P., Francesconi D., Chopra I.J. (2000). A study of iodothyronine 5′-monodeiodinase activities in normal and pathological tissues in man and their comparison with activities in rat tissues. Life Sci..

[615] Galton V.A., McCarthy P.T., St Germain D.L. (1991). The ontogeny of iodothyronine deiodinase systems in liver and intestine of the rat. Endocrinology.

[616] Rodriguez F., Jolin T. (1991). The role of somatostatin and/or dopamine in basal and TRH-stimulated TSH release in food-restricted rats. Acta Endocrinol..

[617] DeRuyter H., Burman K.D., Wartofsky L., Smallridge R.C. (1984). Thyrotropin secretion in starved rats is enhanced by somatostatin antiserum. Horm. Metab. Res..

[618] Bradley D.J., Young W.S., Weinberger C. (1989). Differential expression of α and β thyroid hormone receptor genes in rat brain and pituitary. Proc. Natl. Acad. Sci. USA.

[619] Bernal J. (2007). Thyroid hormone receptors in brain development and function. Nat. Clin. Pract. Endocrinol. Metab..

[620] Horlein A.J., Naar A.M., Heinzel T., Torchia J., Gloss B., Kurokawa R., Ryan A., Kamei Y., Soderstrom M., Glass C.K. (1995). Ligand-independent repression by the thyroid hormone receptor mediated by a nuclear receptor co-repressor. Nature.

[621] Guigon C.J., Zhao L., Lu C., Willingham M.C., Cheng S.Y. (2008). Regulation of β-catenin by a novel nongenomic action of thyroid hormone β receptor. Mol. Cell. Biol..

[622] Bauer M., Whybrow P.C. (2001). Thyroid hormone, neural tissue and mood modulation. World J. Biol. Psychiatry.

[623] Farsetti A., Desvergne B., Hallenbeck P., Robbins J., Nikodem V.M. (1992). Characterization of myelin basic protein thyroid hormone response element and its function in the context of native and heterologous promoter. J. Biol. Chem..

[624] Farsetti A., Mitsuhashi T., Desvergne B., Robbins J., Nikodem V.M. (1991). Molecular basis of thyroid hormone regulation of myelin basic protein gene expression in rodent brain. J. Biol. Chem..

[625] Koibuchi N., Fukuda H., Chin W.W. (1999). Promoter-specific regulation of the brain-derived neurotropic factor gene by thyroid hormone in the developing rat cerebellum. Endocrinology.

[626] Graff M.N., Baas D., Puymirat J., Sarlieve L.L., Delaunoy J.P. (1993). The α and β thyroid receptors are expressed by cultured ependymal cells. Correlation with the effect of l-3,5,3′-triiodothyronine on glutamine synthetase mRNAs. Neurosci. Lett..

[627] Garcia-Fernandez L.F., Rausell E., Urade Y., Hayaishi O., Bernal J., Munoz A. (1997). Hypothyroidism alters the expression of prostaglandin D_2_ synthase/β trace in specific areas of the developing rat brain. Eur. J. Neurosci..

[628] Yen P.M. (2001). Physiological and molecular basis of thyroid hormone action. Physiol. Rev..

[629] Dong H., Yauk C.L., Rowan-Carroll A., You S.H., Zoeller R.T., Lambert I., Wade M.G. (2009). Identification of thyroid hormone receptor binding sites and target genes using ChIP-on-chip in developing mouse cerebellum. PLoS ONE.

[630] Gagne R., Green J.R., Dong H., Wade M.G., Yauk C.L. (2013). Identification of thyroid hormone receptor binding sites in developing mouse cerebellum. BMC Genom..

[631] Nayak B., Hodak S.P. (2007). Hyperthyroidism. Endocrinol. Metab. Clin. N. Am..

[632] Zamoner A., Heimfarth L., Pessoa-Pureur R. (2008). Congenital hypothyroidism is associated with intermediate filament misregulation, glutamate transporters down-regulation and MAPK activation in developing rat brain. Neurotoxicology.

[633] Joffe R.T., Sokolov S.T. (1994). Thyroid hormones, the brain, and affective disorders. Crit. Rev. Neurobiol..

[634] Rabie A., Favre C., Clavel M.C., Legrand J. (1977). Effects of thyroid dysfunction on the development of the rat cerebellum, with special reference to cell death within the internal granular layer. Brain Res..

[635] Rabie A., Legrand J. (1973). Effects of thyroid hormone and undernourishment on the amount of synaptosomal fraction in the cerebellum of the young rat. Brain Res..

[636] Zhou L., Li X., Ahmed A., Wu D., Liu L., Qiu J., Yan Y., Jin M., Xin Y. (2014). Gut microbe analysis between hyperthyroid and healthy individuals. Curr. Microbiol..

[637] Lauritano E.C., Bilotta A.L., Gabrielli M., Scarpellini E., Lupascu A., Laginestra A., Novi M., Sottili S., Serricchio M., Cammarota G. (2007). Association between hypothyroidism and small intestinal bacterial overgrowth. J. Clin. Endocrinol. Metab..

[638] Wu S.Y., Green W.L., Huang W.S., Hays M.T., Chopra I.J. (2005). Alternate pathways of thyroid hormone metabolism. Thyroid.

[639] Rayman M.P. (2012). Selenium and human health. Lancet.

[640] Ventura M., Melo M., Carrilho F. (2017). Selenium and Thyroid Disease: From Pathophysiology to Treatment. Int. J. Endocrinol..

[641] Nguyen T.T., DiStefano J.J., Yamada H., Yen Y.M. (1993). Steady state organ distribution and metabolism of thyroxine and 3,5,3′-triiodothyronine in intestines, liver, kidneys, blood, and residual carcass of the rat in vivo. Endocrinology.

[642] Navarro A.M., Suen V.M., Souza I.M., De Oliveira J.E., Marchini J.S. (2005). Patients with severe bowel malabsorption do not have changes in iodine status. Nutrition.

[643] Michalaki M., Volonakis S., Mamali I., Kalfarentzos F., Vagenakis A.G., Markou K.B. (2014). Dietary iodine absorption is not influenced by malabsorptive bariatric surgery. Obes. Surg..

[644] Vought R.L., Brown F.A., Sibinovic K.H., McDaniel E.G. (1972). Effect of changing intestinal bacterial flora on thyroid function in the rat. Horm. Metab. Res..

[645] Chung S.J., Van M. (1964). Absorption of Thyroxine from the Small Intestine of Rats. Endocrinology.

[646] DiStefano J.J., de Luze A., Nguyen T.T. (1993). Binding and degradation of 3,5,3′-triiodothyronine and thyroxine by rat intestinal bacteria. Am. J. Physiol..

[647] Salvatore G., Covelli I., Roche J. (1963). The fixation of thyroid hormones by Escherichia coli and its mechanism. Gen. Comp. Endocrinol..

[648] Mehdi Y., Hornick J.L., Istasse L., Dufrasne I. (2013). Selenium in the environment, metabolism and involvement in body functions. Molecules.

[649] Drutel A., Archambeaud F., Caron P. (2013). Selenium and the thyroid gland: More good news for clinicians. Clin. Endocrinol..

[650] Lavu R.V., Van De Wiele T., Pratti V.L., Tack F., Du Laing G. (2016). Selenium bioaccessibility in stomach, small intestine and colon: Comparison between pure Se compounds, Se-enriched food crops and food supplements. Food Chem..

[651] Hrdina J., Banning A., Kipp A., Loh G., Blaut M., Brigelius-Flohe R. (2009). The gastrointestinal microbiota affects the selenium status and selenoprotein expression in mice. J. Nutr. Biochem..

[652] Kasaikina M.V., Kravtsova M.A., Lee B.C., Seravalli J., Peterson D.A., Walter J., Legge R., Benson A.K., Hatfield D.L., Gladyshev V.N. (2011). Dietary selenium affects host selenoproteome expression by influencing the gut microbiota. FASEB J..

[653] Nguyen T.T., DiStefano J.J., Huang L.M., Yamada H., Cahnmann H.J. (1993). 5′- and 5-deiodinase activities in adult rat cecum and large bowel contents inhibited by intestinal microflora. Am. J. Physiol..

[654] Kim J., Combs G.F. (1997). Effects of selenium on colonic fermentation in the rat. Biol. Trace Elem. Res..

[655] Kryukov G.V., Gladyshev V.N. (2004). The prokaryotic selenoproteome. EMBO Rep..

[656] Rother M., Resch A., Wilting R., Bock A. (2001). Selenoprotein synthesis in archaea. Biofactors.

[657] Gladyshev V.N., Khangulov S.V., Stadtman T.C. (1996). Properties of the selenium- and molybdenum-containing nicotinic acid hydroxylase from Clostridium barkeri. Biochemistry.

[658] Schrader T., Rienhofer A., Andreesen J.R. (1999). Selenium-containing xanthine dehydrogenase from Eubacterium barkeri. Eur. J. Biochem..

[659] Visser T.J. (1996). Pathways of thyroid hormone metabolism. Acta Med. Austriaca.

[660] Boelen A., Kwakkel J., Thijssen-Timmer D.C., Alkemade A., Fliers E., Wiersinga W.M. (2004). Simultaneous changes in central and peripheral components of the hypothalamus-pituitary-thyroid axis in lipopolysaccharide-induced acute illness in mice. J. Endocrinol..

[661] Boelen A., Kwakkel J., Platvoet-ter Schiphorst M., Mentrup B., Baur A., Koehrle J., Wiersinga W.M. (2004). Interleukin-18, a proinflammatory cytokine, contributes to the pathogenesis of non-thyroidal illness mainly via the central part of the hypothalamus-pituitary-thyroid axis. Eur. J. Endocrinol..

[662] de Herder W.W., Hazenberg M.P., Pennock-Schroder A.M., Hennemann G., Visser T.J. (1986). Rapid and bacteria-dependent in vitro hydrolysis of iodothyronine-conjugates by intestinal contents of humans and rats. Med. Biol..

[663] Hazenberg M.P., de Herder W.W., Visser T.J. (1988). Hydrolysis of iodothyronine conjugates by intestinal bacteria. FEMS Microbiol. Rev..

[664] Otten M.H., Herder W.W., Hazenberg M.P., Boom M., Hennemann G. (1983). Iodothyronine sulfatase activity of two anaerobic bacterial strains from rat intestinal microflora. FEMS Microbiol. Lett..

[665] Rutgers M., Heusdens F.A., Bonthuis F., de Herder W.W., Hazenberg M.P., Visser T.J. (1989). Enterohepatic circulation of triiodothyronine (T_3_) in rats: Importance of the microflora for the liberation and reabsorption of T_3_ from biliary T_3_ conjugates. Endocrinology.

[666] Beigneux A.P., Moser A.H., Shigenaga J.K., Grunfeld C., Feingold K.R. (2003). Sick euthyroid syndrome is associated with decreased TR expression and DNA binding in mouse liver. Am. J. Physiol. Endocrinol. Metab..

[667] Castro I., Quisenberry L., Calvo R.M., Obregon M.J., Lado-Abeal J. (2013). Septic shock non-thyroidal illness syndrome causes hypothyroidism and conditions for reduced sensitivity to thyroid hormone. J. Mol. Endocrinol..

[668] Virili C., Bassotti G., Santaguida M.G., Iuorio R., Del Duca S.C., Mercuri V., Picarelli A., Gargiulo P., Gargano L., Centanni M. (2012). Atypical celiac disease as cause of increased need for thyroxine: A systematic study. J. Clin. Endocrinol. Metab..

[669] Centanni M., Marignani M., Gargano L., Corleto V.D., Casini A., Delle Fave G., Andreoli M., Annibale B. (1999). Atrophic body gastritis in patients with autoimmune thyroid disease: An underdiagnosed association. Arch. Intern. Med..

[670] Cindoruk M., Tuncer C., Dursun A., Yetkin I., Karakan T., Cakir N., Soykan I. (2002). Increased colonic intraepithelial lymphocytes in patients with Hashimoto’s thyroiditis. J. Clin. Gastroenterol..

[671] Sasso F.C., Carbonara O., Torella R., Mezzogiorno A., Esposito V., Demagistris L., Secondulfo M., Carratu R., Iafusco D., Carteni M. (2004). Ultrastructural changes in enterocytes in subjects with Hashimoto’s thyroiditis. Gut.

[672] Adeniyi K.O., Olowookorun M.O. (1989). Gastric acid secretion and parietal cell mass: Effects of thyroidectomy and thyroxine. Am. J. Physiol..

[673] Tseng C.C., Johnson L.R. (1986). Role of thyroxine in functional gastric development. Am. J. Physiol..

[674] Jetten A.M. (2009). Retinoid-related orphan receptors (RORs): Critical roles in development, immunity, circadian rhythm, and cellular metabolism. Nucl. Recept. Signal..

[675] Yang X.O., Pappu B.P., Nurieva R., Akimzhanov A., Kang H.S., Chung Y., Ma L., Shah B., Panopoulos A.D., Schluns K.S. (2008). T helper 17 lineage differentiation is programmed by orphan nuclear receptors ROR α and ROR γ. Immunity.

[676] Yissachar N., Zhou Y., Ung L., Lai N.Y., Mohan J.F., Ehrlicher A., Weitz D.A., Kasper D.L., Chiu I.M., Mathis D. (2017). An Intestinal Organ Culture System Uncovers a Role for the Nervous System in Microbe-Immune Crosstalk. Cell.

